# Effects of Argentilactone on the Transcriptional Profile, Cell Wall and Oxidative Stress of *Paracoccidioides* spp.

**DOI:** 10.1371/journal.pntd.0004309

**Published:** 2016-01-06

**Authors:** Felipe Souto Araújo, Luciene Melo Coelho, Lívia do Carmo Silva, Benedito Rodrigues da Silva Neto, Juliana Alves Parente-Rocha, Alexandre Melo Bailão, Cecília Maria Alves de Oliveira, Gabriel da Rocha Fernandes, Orville Hernández, Juan Guillermo McEwen Ochoa, Célia Maria de Almeida Soares, Maristela Pereira

**Affiliations:** 1 Laboratório de Biologia Molecular, Instituto de Ciências Biológicas, Universidade Federal de Goiás, Goiânia, Goiás, Brazil; 2 Laboratório de Produtos Naturais, Instituto de Química, Universidade Federal de Goiás, Goiânia, Goiás, Brazil; 3 Laboratório de Biodados, Biologia Celular e Desenvolvimento, Universidade Católica de Brasília, Brasília, Distrito Federal, Brazil; 4 Unidad de Biología Celular y Molecular, Corporación para Investigaciones Biológicas (CIB) and Escuela de Microbiología Universidad de Antioquia, Medellín, Colombia; 5 Unidad de Biología Celular y Molecular, Corporación para Investigaciones Biológicas (CIB) and Facultad de Medicina Universidad de Antioquia, Medellín, Colombia; University of California San Diego School of Medicine, UNITED STATES

## Abstract

*Paracoccidioides* spp., a dimorphic pathogenic fungus, is the etiologic agent of paracoccidioidomycosis (PCM). PCM is an endemic disease that affects at least 10 million people in Latin America, causing severe public health problems. The drugs used against pathogenic fungi have various side effects and limited efficacy; therefore, there is an inevitable and urgent medical need for the development of new antifungal drugs. In the present study, we evaluated the transcriptional profile of *Paracoccidioides lutzii* exposed to argentilactone, a constituent of the essential oil of *Hyptis ovalifolia*. A total of 1,058 genes were identified, of which 208 were up-regulated and 850 were down-regulated. Cell rescue, defense and virulence, with a total of 26 genes, was a functional category with a large number of genes induced, including heat shock protein 90 (*hsp*90), cytochrome c peroxidase (*ccp*), the hemoglobin ligand RBT5 (*rbt5*) and superoxide dismutase (*sod*). Quantitative real-time PCR revealed an increase in the expression level of all of those genes. An enzymatic assay showed a significant increase in SOD activity. The reduced growth of *Pbhsp90*-aRNA, *Pbccp*-aRNA, *Pbsod*-aRNA and *Pbrbt5*-aRNA isolates in the presence of argentilactone indicates the importance of these genes in the response of *Paracoccidioides* spp. to argentilactone. The response of the P. *lutzii* cell wall to argentilactone treatment was also evaluated. The results showed that argentilactone caused a decrease in the levels of polymers in the cell wall. These results suggest that argentilactone is a potential candidate for antifungal therapy.

## Author Summary

Paracoccidioidomycosis (PCM) is a neglected human systemic mycosis caused by *Paracoccidioides* spp. fungus that invades the host’s lungs and can disseminate to many other organs. Treatment usually involves amphotericin B, sulfadiazine, trimethoprim-sulfamethoxazole, itraconazole, ketoconazole or fluconazole for six months to two years. In this way, many adverse effects are associated with treatment, and patients can have many co-morbidities and difficulties in complying with treatment. For those reasons, more effective and less toxic drugs are needed. The discovery of a potentially bioactive molecule and its correlation with a biological target is an important step in the research and development of drugs. One of the ways in which cells adjust to environmental change is by changing the pattern of gene expression. Thus, the transcriptome is potential experimental strategy to elucidate the mode of action of bioactive molecules. Here, *Paracoccidoides* spp. altered the expression of genes, leading to a further understanding of the action of the compound argentilactone in the fungal cells. Argentilactone seems to be able to modulate cellular targets, to induce oxidative stress and to interfere with the biosynthesis of the *P*. *lutzii* cell wall.

## Introduction

The genus Paracoccidioides, which comprises the species *lutzii* and *brasiliensis*, is the etiological agent of paracoccidioidomycosis (PCM), an important systemic mycosis in Latin America. The inhalation of mycelia fragments, infectious forms of the fungus, is the common route of infection that primarily affects the lungs [1]. PCM has been reported to affect individuals from northern Argentina to southern Mexico, with prevalence in Brazil, Colombia, Venezuela and Argentina. The most cases of PCM occur in men, rural workers and individuals between 30–50 years of age, although it affects individuals at any age [2]. In Brazil, PCM is responsible for over 50% of deaths caused by fungal infections [3].

Fungal infections are a serious threat to public health due to their association with high rates of morbidity and mortality [4]. Despite the existence of potent antifungal agents, the development of antifungal resistance by the fungal species, as well as cytotoxicity and collateral effects, has limited the use of current antifungals [5]. In addition, PCM treatment is a slow process that extends over months or years depending on the severity of the disease and the site of injury [6]. Given these facts, it is important to search for and identify novel antifungals.

New therapeutic approaches have been suggested for PCM [7]. In this way, our group has identified new antifungal targets, such as the enzymes 1,3-β-D-glucan synthase [8,9], malate synthase [10,11], isocitrate lyase [12] and (S)-adenosyl-L-methionine: C24 sterol methyl transferase [13], from *Paracoccidioides* spp. In addition, we have investigated new antifungal compounds, including thiosemicarbazide [14] and oenothein [15,16]. Here, as part of a continuing search for diverse chemicals from plants, we have examined argentilactone, a bioactive metabolite isolated from *Hyptis ovalifolia*, which is renowned for its wide range of anticancer, insecticidal and antimicrobial activities [17,18], including those against *P*. *lutzii*.

In our previous work, we have investigated the antifungal potential of argentilactone and its semi-synthetic derivatives on *P*. *lutzii* [19]. Argentilactone and the tetrahydro derivative inhibited native and recombinant isocitrate lyase from *P*. *lutzii* in the presence of glucose and acetate. Additionally, argentilactone and the tetrahydro derivative exhibited inhibitory activity against *P*. *lutzii* yeast cells and dose-dependently interfered with the dimorphic transition from the mycelium to the yeast phase. Argentilactone interfered with the viability of *Paracoccidioides* spp., but was not toxic to MRC5 cells at the IC_50_ concentration in the fungus. *In silico* studies showed that argentilactone and reduced argentilactone bound to the catalytic site of *Pb*ICL, and the amino acids involved in their binding were identified. The data obtained indicate that argentilactone is a potential candidate for antifungal therapy.

In this study, we investigated the transcriptional profile of *P*. *lutzii* yeast cells grown in the presence of argentilactone using the Illumina/Hiseq^™^2000 platform (Illumina, San Diego, CA, USA). A total of 1,058 genes were identified, of which 208 were up-regulated and 850 were down-regulated in response to argentilactone treatment. The genes identified were classified by the biological function of the encoded protein products. The main categories identified in the up-regulated genes were metabolism, cell rescue, defense and virulence, energy and cell cycle and DNA processing. The down-regulated gene categories were related to metabolism, transcription, protein fate and cell cycling and DNA processing.

## Materials and Methods

### Extraction of argentilactone (2H- Pyran- 2- one, 6- (1- heptenyl) - 5, 6- dihydro-, [R- (Z)])

The essential oil of *H*. *ovalifolia* was obtained as described previously, and the NMR data are consistent with the literature [18].

### Culture conditions of *Paracoccidioides* spp.

*P*. *lutzii* (ATCC-MYA-826) was used in the experiments described in this study, except for the silenced mutant experiments, which were performed with *P*. *brasiliensis* strains ATCC 60855 and *Pb*339. The yeast phase was maintained at 36°C in Fava Netto’s semi-solid medium [20] containing 1% (w/v) peptone, 0.5% (w/v) yeast extract, 0.3% (w/v) proteose peptone, 0.5% (w/v) beef extract, 0.5% (w/v) NaCl, 4% (w/v) glucose, and 1.4% (w/v) agar, pH 7.2. For experiments, cells were transferred to Fava Netto’s liquid medium, where they remained for 72 h at 36°C under agitation at 150 rpm. Afterwards, the fungus was transferred into McVeigh Morton (MMcM) chemically defined liquid medium [21] and incubated for 16 h with agitation at 150 rpm.

The viability of *P*. *lutzii* cells grown in the absence or presence of 9 μg/mL argentilactone was determined using the trypan blue method [22], in which viable and non-viable cells are counted in a Neubauer chamber. Except for the transcriptional profiling of *P*. *lutzii* yeast cells, all experiments were done using yeast cells from three different seedings from different days.

### Extraction and quantification of RNA

All procedures for extraction and manipulation of total RNA were performed in RNAse-free conditions. Total RNA from *Paracoccidioides* spp. yeast cells not treated or treated with 9 μg/mL argentilactone for 6 h at 36°C in MMcM liquid medium was extracted using Trizol (Invitrogen, Carlsbad, CA, USA) according to the supplier's instructions. Each experiment (not treated and treated) was performed in triplicate and pooled. The mRNA was purified using the GenElute mRNA kit (Sigma Aldrich, St. Louis, MO, USA). Total RNA was quantified on a NanoDrop 8000 Spectrophotometer and stored at -80°C. Total RNA integrity was visualized using an agarose gel.

### High-throughput mRNA sequencing (RNA-seq)

The cDNA libraries were prepared from poly(A)-fragment selected mRNA and processed on the Illumina HiSeq^™^2000 Sequencing System (http://www.illumina.com). The pipeline was performed as described previously [23]. Briefly, the sequencing reads were mapped to reference the *P*. *lutzii* genome (http://www.broadinstitute.org/annotation/genome/paracoccidioides_brasiliensis/Multiome.html) using the Bowtie 2 tool. Mapped read data were analyzed by the DEGseq package. Each read was allowed to align to just one site of the genome, and the reads were counted. The default parameters were used to perform the alignment. The number of mismatches allowed in seed alignment (-N) was 0, and the length of each seed (-L) was 20. The fold change selection method was used for differentially expressed gene selection using Fisher’s exact test, and a *p*-value of 0.001 was considered to select the genes. From the selected genes, a 1.5-fold change cut-off was considered. Genes with log_2_ (fold change) higher than 0.58 or less than -0.58 were selected and classified as up- and down-regulated genes, respectively. Genes’ identifications and annotations were determined from the *P*. *lutzii* genome database (http://www.broadinstitute.org/annotation/genome/paracoccidioides_brasiliensis/MultiHome.html). The biological processes were obtained using the Pedant on MIPS (http://pedant.helmholtz-muenchen.de/pedant3htmlview/pedant3view?Method=analysis&Db=p3_r48325_Par_brasi_*Pb*01), which provides a tool to browse and search the functional categories (FunCat) of proteins. Additionally, hypothetical proteins were annotated using the Blast program (https://blast.ncbi.nlm.nih.gov/Blast.cgi?PROGRAM=blastp&PAGE_TYPE=BlastSearch&LINK_LOC=blasthome).

### Determination of the susceptibility of *Paracoccidioides brasiliensis* and *Pbhsp90*-aRNA, *Pbccp*-aRNA, *Pbsod*-aRNA, and *Pbrbt5*-aRNA isolates to argentilactone

The argentilactone sensitivity assay was performed from three independent experiments using *P*. *brasiliensis*, *Pb*339, *Pb*60855, *Pb*60855EV, *Pb*339EV and the silenced mutants for superoxide dismutase (SOD) protein (*Pbsod*-aRNA) [14], heat shock protein HSP90 (*Pbhsp90*-aRNA) [24], cytochrome C peroxidase protein (*Pbccp*-aRNA) [25] and hemoglobin ligand protein RBT5 (*Pbrbt5*-aRNA) [26] were grown in liquid Fava-Netto for 72 h with agitation at 150 rpm at 36°C, and then transferred to MMcM liquid medium, where they remained overnight. The cells were then washed with phosphate buffered saline (PBS 1X) and diluted to concentrations of 10^4^, 10^5^ and 10^6^. The cells were plated in solid Fava-Netto medium supplemented with argentilactone at concentrations of 4.5 μg/mL, 9 μg/mL, 18 μg/mL and 36 μg/mL and incubated for 6 days at 36°C. The control was prepared in the absence of argentilactone.

### Gene expression analysis by quantitative real-time PCR (qRT-PCR)

Total RNA was extracted using Trizol reagent (Invitrogen, Carlsbad, CA, USA) following the manufacturer’s protocol. The RNA was reverse transcribed using the high-capacity RNA-to-cDNA kit (Applied Biosystems, Foster City, CA, USA). The cDNA was quantified by qRT-PCR using a SYBR green PCR master mix (Applied Biosystems Step One Plus PCR System). α-tubulin was used as endogenous control for data normalization, and its amplification was presented as relative expression in comparison to that of the experimental samples, whose value was set to 1. Data were expressed as the mean ± standard deviation of the biological triplicates of independent experiments. Standard curves were generated by diluting the cDNA solution 1:5. Relative expression levels of genes of interest were calculated using the standard curve method for relative quantification. Statistical comparisons were performed using Student’s t test and *p*-values < 0.05 were considered statistically significant. The specific sense and antisense primers are listed in S1 Table. Argentilactone-regulated transcripts were selected for qRT-PCR validation assays.

### Protein extraction

The protein extraction was performed with cells grown in the presence and absence of argentilactone. After incubation with 9 μg/mL argentilactone for 6 h in MMcM liquid medium, the cells were centrifuged at 10,000 x *g* for 15 min at 4°C and protein was extracted using extraction buffer (20 mM Tris-HCl pH 8.8; 2 mM CaCl_2_) containing a mixture of protease inhibitors (GE Healthcare). After the addition of glass beads (0.45 mm), the cells were lysed in a bead-beater, followed by centrifugation at 10,000 x *g* for 15 min at 4°C. The supernatant was collected, and the protein concentrations were determined using Bradford reagent (Sigma-Aldrich) [27].

### Assay of superoxide dismutase enzymatic activity

The superoxide dismutase activity was quantified using an SOD assay kit (Sigma-Aldrich) that determine to production of formazan dye upon reduction with O_2_^-^ by colorimetric detection at 440 nm. The levels of SOD activity were quantified using 1 μg of total protein extract. Enzyme activity data were plotted as the mean of three independent experiments. The statistical analysis was performed using Student’s t-test and samples with a *p*-value ˂ 0.05 were considered statistically significant.

### Fluorescence microscopy

Fluorescence microscopy assays were performed as previously described [28]. Calcofluor White (CFW) and Congo Red (CR) (Sigma-Aldrich) were used to label the cell wall of *P*. *lutzii* yeast cells. A total of 10^6^ cells/mL *P*. *lutzii* yeast cells were inoculated in Fava Netto’s liquid medium and grown for 3 days with agitation at 150 rpm. Afterwards, the cultures were incubated in MMcM liquid medium overnight at 36°C with shaking for 16 h. The cells were centrifuged at 5,000 x *g* for 5 min and then transferred to MMcM liquid medium containing 9 μg/mL argentilactone for 6 h. Control cells were incubated in MMcM liquid medium without argentilactone. Cells were fixed in 100% methanol at -80°C for 20 min, and then at -20°C for 20 min, and finally they were washed and centrifuged. The collected cells were stained with 100 μg/mL CR and CFW in PBS for 15 min and washed with 1x PBS. The samples were analyzed under a fluorescence microscope at 345 nm and 500 nm for CR and CFW, respectively (Zeiss Axiocam MRc—Scope A1, Carl Zeiss, Jena, Germany).

### Ethanol dosing

The level of ethanol was measured as previously described [23] using an enzymatic detection kit UV-test for ethanol (RBiopharm, Darmstadt, Germany). A total of 1 x 10^6^ cells were grown in the presence and absence of the argentilactone. Afterwards, the protein extract was obtained after cell lysis using glass beads and a bead beater apparatus (BioSpec, Oklahoma, USA) in 5 cycles of 30 sec. The cell lysate was subjected to centrifugation for 15 min, at 4°C, at 10,000 × *g*. The enzyme assay was performed in triplicate using the supernatant according to the manufacturer's instructions.

### Mitochondrial membrane potential measurement

The mitochondrial membrane was monitored using the rhodamine 123 fluorescent dye. A total of 10^6^ cells/mL were treated with 9 μg/mL argentilactone for 6 h. After treatment, the cells were centrifuged and incubated with 20 μM rhodamine 123 for 20 min at room temperature. Afterwards, the cells were washed 2 times with 1X PBS and resuspended in 1 mL PBS for analysis using a guava easyCyte flow cytometer with excitation and emission wavelengths of 488 and 530 nm, respectively.

## Results

### Transcriptional response pattern of *Paracoccidioides lutzii* to argentilactone

Next-generation sequencing was used to produce the transcriptional profile. Approximately 46 million reads of 100-bp single-end sequences were obtained. The data were submitted in the ncbi.nlm.nih.gov, generating access number SRP064389. The reads were mapped using the reference genome of the *P*. *lutzii* genome database (http://www.broadinstitute.org/annotation/genome/paracoccidioides_brasiliensis/MultiHome.html) and were analyzed using the DEGseq package. For the global analysis, plotting graphs were prepared. The number of reads counted for each transcript in the presence or absence of argentilactone was represented by scattered dots. The transcripts are represented by dots, which could represent a different number of reads in each condition (S1A Fig). The statistical test was applied to identify differentially expressed transcripts, represented by red dots (S1B Fig).

**Fig 1:**
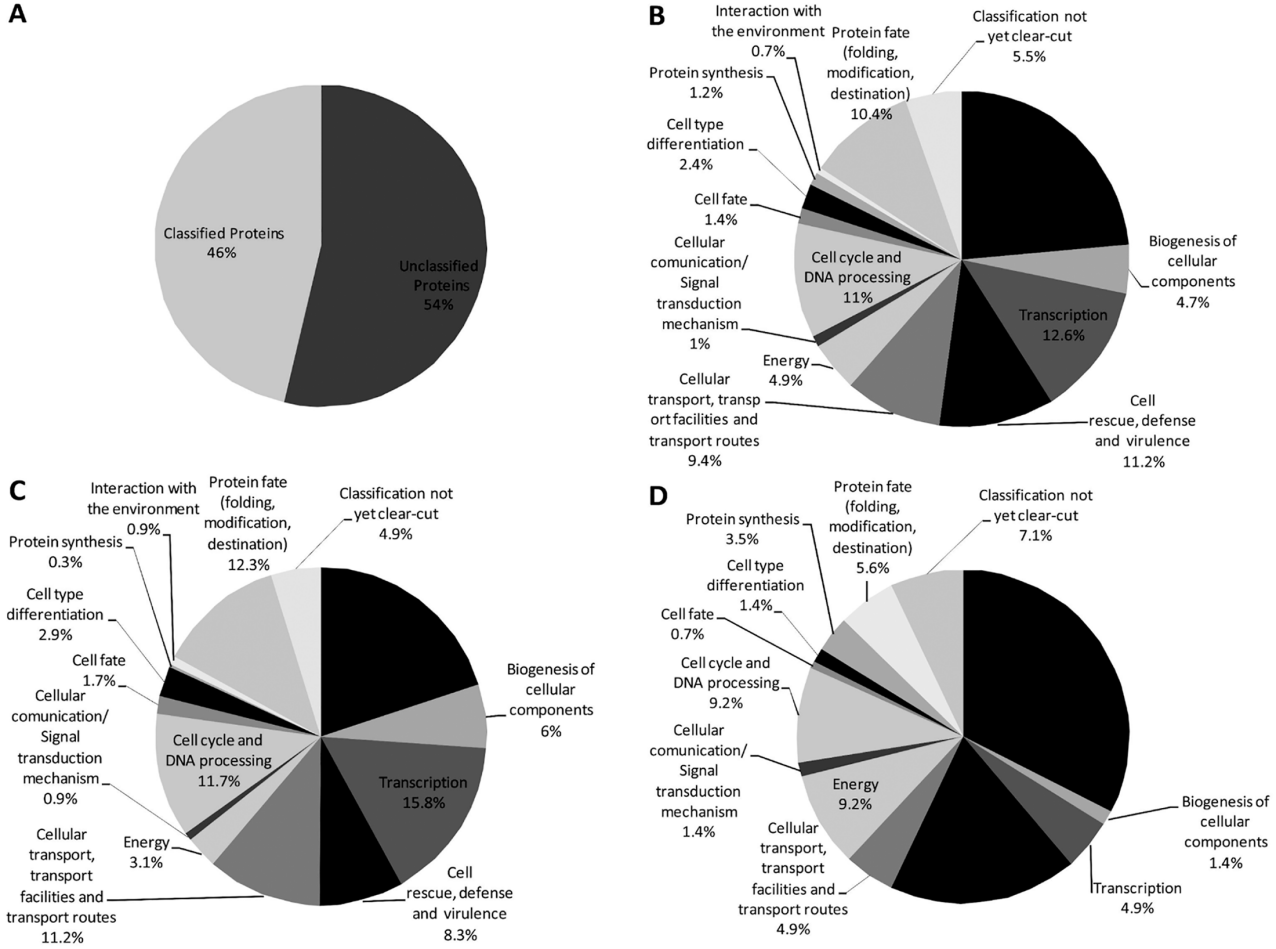
Functional classification and abundance levels of transcripts regulated in *Paracoccidioides* lutzii in the presence of argentilactone obtained by RNAseq. **(A)** Total transcripts represented by classified and unclassified categories; **(B)** Genes differentially expressed in the presence of argentilactone; **(C)** Down-regulated genes in the presence of argentilactone; **(D)** Up-regulated genes in the presence of argentilactone. Counting the number of reads for each gene in the genome annotated reference was applied to a statistical analysis using a Fisher's exact test for the identification of genes with differential expression.

To determine the up- and down-regulated transcripts (S2 and S3 Tables, respectively), a cut-off of 1.5-fold change was used, resulting in 1,058 differentially expressed transcripts in *P*. *lutzii* yeast cells. A biological process classification was performed to gain a general understanding of the functional categories affected by argentilactone. A total of 54% (567 transcripts) were represented by proteins of unknown function (Fig 1A). After 6 h of exposure to argentilactone, transcripts associated with metabolism (23.6%) were the most represented. Other groups were also regulated, such as transcription (12.6%), cell rescue, defense and virulence (11.2%) and cell cycle and DNA processing (11%) (Fig 1B). The genes related to metabolism (20%), transcription (15.8%), protein fate (12.3%), cell cycle and DNA processing (11.7%) and cellular transport, transport facilities and transport routes (11.2%) (Fig 1C) were down-regulated. The up-regulated genes were mainly related to metabolism (32.4%), cell rescue, defense and virulence (18.3%), cell cycle and DNA processing (9.2%), energy (9.2%) and protein fate (5.6%) (Fig 1D).

### Argentilactone seems interferes with urea synthesis and excretion in *Paracoccidioides lutzii*

From the transcriptional data obtained (S2 and S3 Tables), we could infer that, we could infer that *P*. *lutzii* down-regulated glutamine synthase, which catalyzes the condensation of glutamate and ammonia to glutamine, and glutamate dehydrogenase, which converts glutamate to α-ketoglutarate, and vice versa. In addition, two glutamate-1-semialdehyde 2,1-aminomutase, which converts glutamate 1-semialdehyde to 5-aminolevulinate, was down-regulated. Glutamate 1-semialdehyde is a molecule formed from glutamate and is a precursor to ornithine and proline. The cofactor of glutamate-1-semialdehyde 2,1-aminomutase is pyridoxal phosphate, which is produced from pyridoxal by pyridoxine kinase, was also down-regulated. On the other hand, in the presence of argentilactone, *P*. *lutzii* seems to use the amide group of xanthine, a purine base, in the synthesis of uric acid, which is excreted and metabolized into allantoic acid, allantoin and urea through an amphibian-like uricolytic pathway [29], as xanthine dehydrogenase, uricase and allantoinase were up-regulated. The production of putrescine and polyamines from ornithine is absent, as ornithine decarboxylase is down-regulated.

### Effect of argentilactone on the metabolism of *Paracoccidioides lutzii*

**Fig 2:**
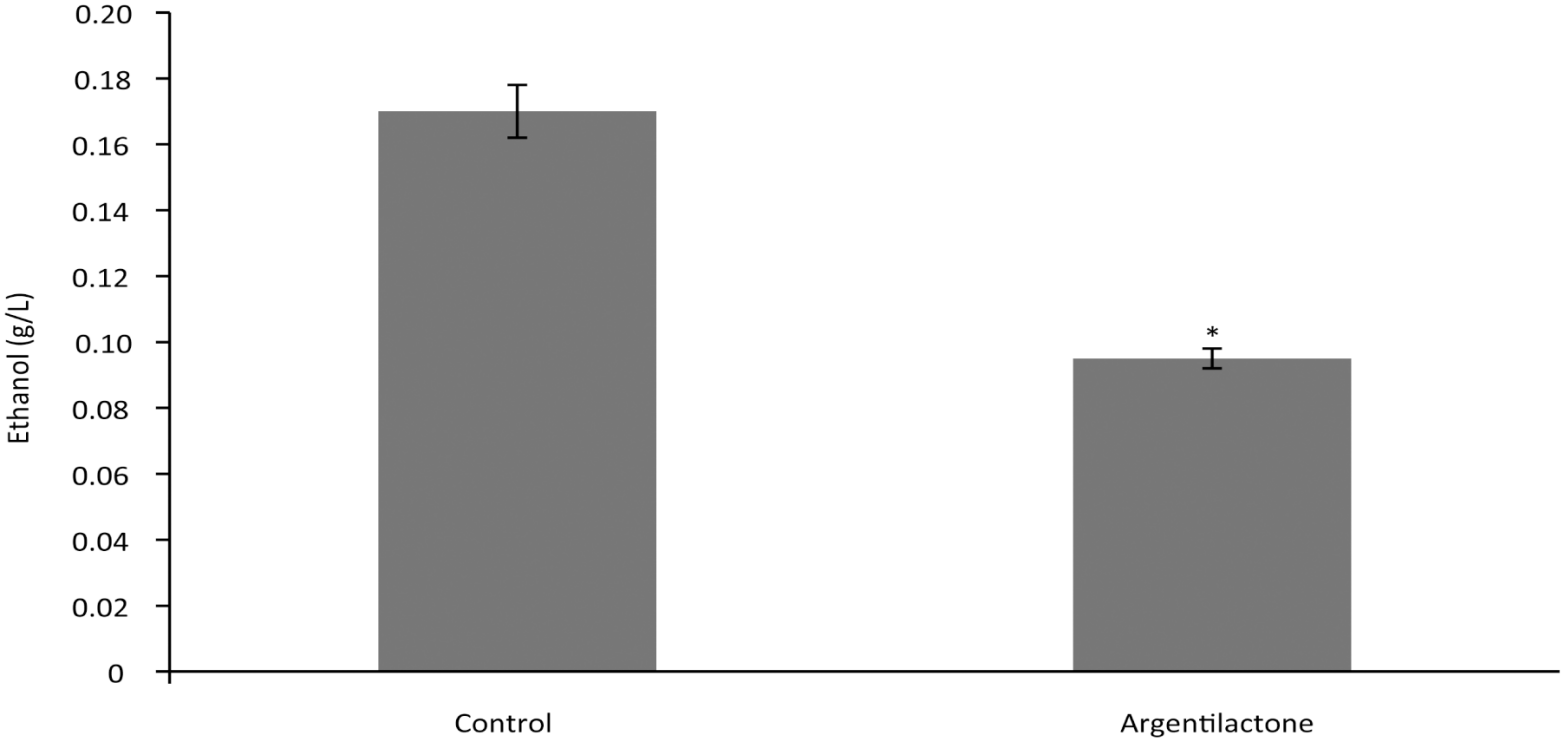
Ethanol dosage in *Paracoccidioides lutzii* under the action of argentilactone. A total of 10^6^ cells were used for each sample, and the ethanol levels in the presence and absence of argentilactone were quantified using enzymatic detection. The data are expressed as the mean ± standard deviation of biological triplicates of independent experiments. Student's *t*-test was used. *, significantly different from the carbon condition at a *p-*value of ≤ 0.05.

Glycolysis is induced in *P*. *lutzii* in the presence of argentilactone, as class II aldolase and glyceraldehyde-3-phosphate dehydrogenase are up-regulated. It is noteworthy that several transporters, including sugar and amino acid transporters, were down-regulated. Although alcoholic fermentation is up-regulated in *P*. *lutzii* yeast cells [30], the presence of argentilactone repressed it because two alcohol dehydrogenases are down-regulated. The ethanol dosage assay confirmed these data, as ethanol is reduced in the presence of argentilactone (Fig 2).

Homogentisate 1,2-dioxygenase catalyzes the conversion of homogentisate to 4-maleylacetoacetate. Hydroxymethylglutaryl-CoA lyase, which converts β-hydroxy-β-methylglutaryl-CoA to acetoacetate and acetyl-CoA, and acetyl-CoA hydrolase, which converts acetyl-CoA to acetate, were up-regulated. *P*. *lutzii* utilizes β-oxidation to obtain energy, as acyl-CoA dehydrogenase, which converts fatty acyl-CoA into trans-2-enoyl-CoA, four enoyl-CoA hydratases, which convert trans-2-enoyl-CoA to L-β-hydroxy-acyl-CoA, and 3-ketoacyl-CoA thiolase B, which converts β-ketoacyl-CoA to acyl-CoA-fatty acid and acetyl-CoA, were up-regulated. The methylcitrate cycle, one of the major pathways for propionyl-CoA metabolism, is an alternative source of carbon through pyruvate production [31]. Here, 2-methylcitrate dehydratase, which participates in the methylcitrate pathway, was up-regulated.

### Argentilactone reduces carbohydrate polymer levels in the *Paracoccidioides lutzii* cell wall

The *P*. *lutzii* cell wall seems to be affected by argentilactone, as several transcripts associated with the synthesis of chitin and glucan, the major components of the fungal cell wall, including that of *Paracoccidioides* [32,33], were down-regulated. Among the enzymes are endo-1,3(4)-β-glucanase, two glucan 1,3-β-glucosidases, β-glucan synthesis-associated protein and glucanase, which are related to the biosynthesis of glucan, and endochitinase, chitin deacetylase, UDP-N-acetylglucosamine pyrophosphorylase, UDP-N-acetylglucosamine transporter YEA4, chitin synthase B, chitin synthase regulator 3 and chitin synthase export chaperone, which are related to the biosynthesis of chitin.

**Fig 3:**
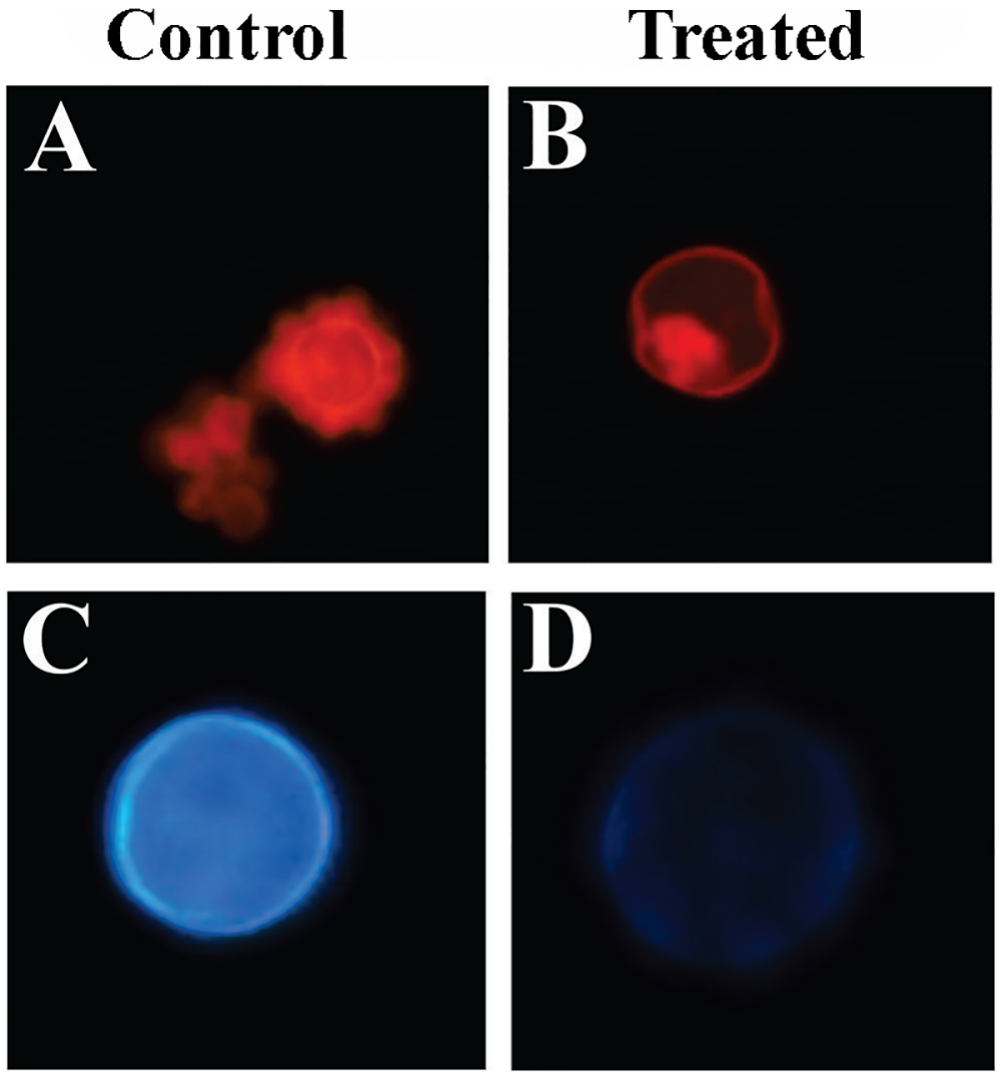
Effect of argentilactone on the polymer levels of the *Paracoccidioides lutzii* cell wall. Fluorescence microscopy showing yeast cells stained by CFW and CR: **(A)** *P*. *lutzii* control stained with CR; **(B)** *P*. *lutzii* after treatment with argentilactone, stained with CR; **(C)** *P*. *lutzii* control stained with CFW; **(D)** *P*. *lutzii* after treatment argentilactone, stained with CFW.

To investigate the chitin and glucan levels in the cell wall, the cells were stained with CFW and CR and visualized by fluorescence microscopy. The fluorochrome CR and CFW interact with polysaccharides of cell wall [34] exhibiting strong affinity for chains of chitin [35,36]. *P*. *lutzii* yeast cells after treatment with argentilactone showed a decrease in fluorescence, indicating a reduction in the levels of this polymer (Fig 3), corroborating the transcriptional data.

### Hypersensitivity of the *Pbsod*-aRNA, *Pbhsp90*-aRNA, *Pbccp*-aRNA and *Pbrbt5*-aRNA mutants to argentilactone

**Fig 4:**
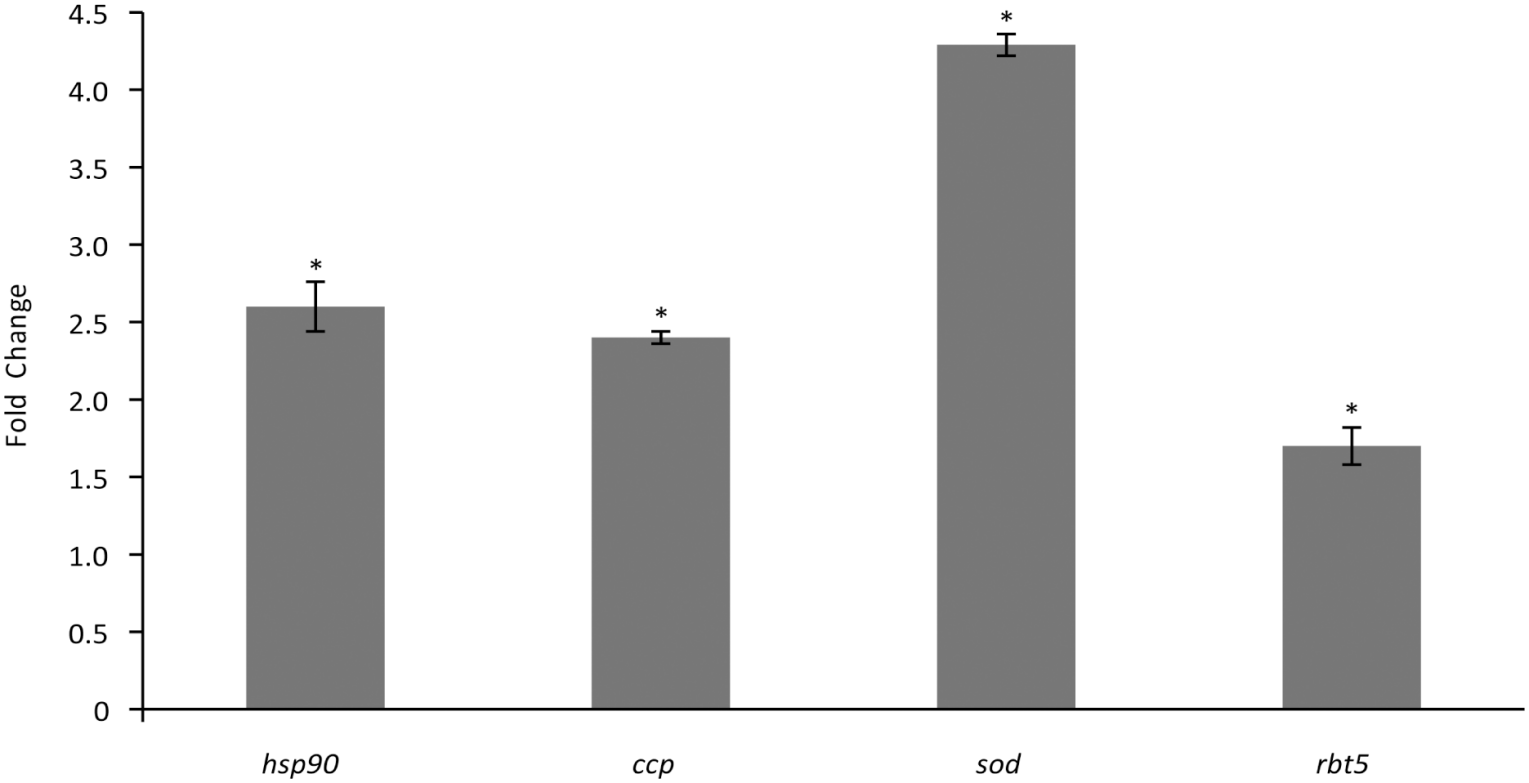
Effect of argentilactone on transcript expression levels in *Paracoccidioides lutzii* yeast cells. The expression levels of *hsp90*, *ccp*, *sod* and *rbt5* genes in *Paracoccidoides* spp. yeast cells grown in MMcM liquid medium with or without argentilactone were analyzed. The data were normalized using the constitutive gene encoding α-tubulin as the endogenous control and are presented as relative expression in comparison to the experimental control cells, whose value was set to 1. Data are expressed as the mean ± standard deviation of the triplicates of independent experiments. *, significantly different from the control at a *p-*value of ˂ 0.05.

Several genes responding to stress were up-regulated. Among these genes are *sod*, *rbt5*, *ccp*, *hsp90*, heat shock protein 10 (*hsp10*) and heat shock protein SSC1 (*hspssc1*). The increased expression levels of *hsp90*, *sod*, *rbt5* and *ccp* were confirmed by qRT-PCR in *P*. *lutzii* (Fig 4) and *P*. *brasiliensis* (S2 Fig), corroborating the transcriptional data.

**Fig 5:**
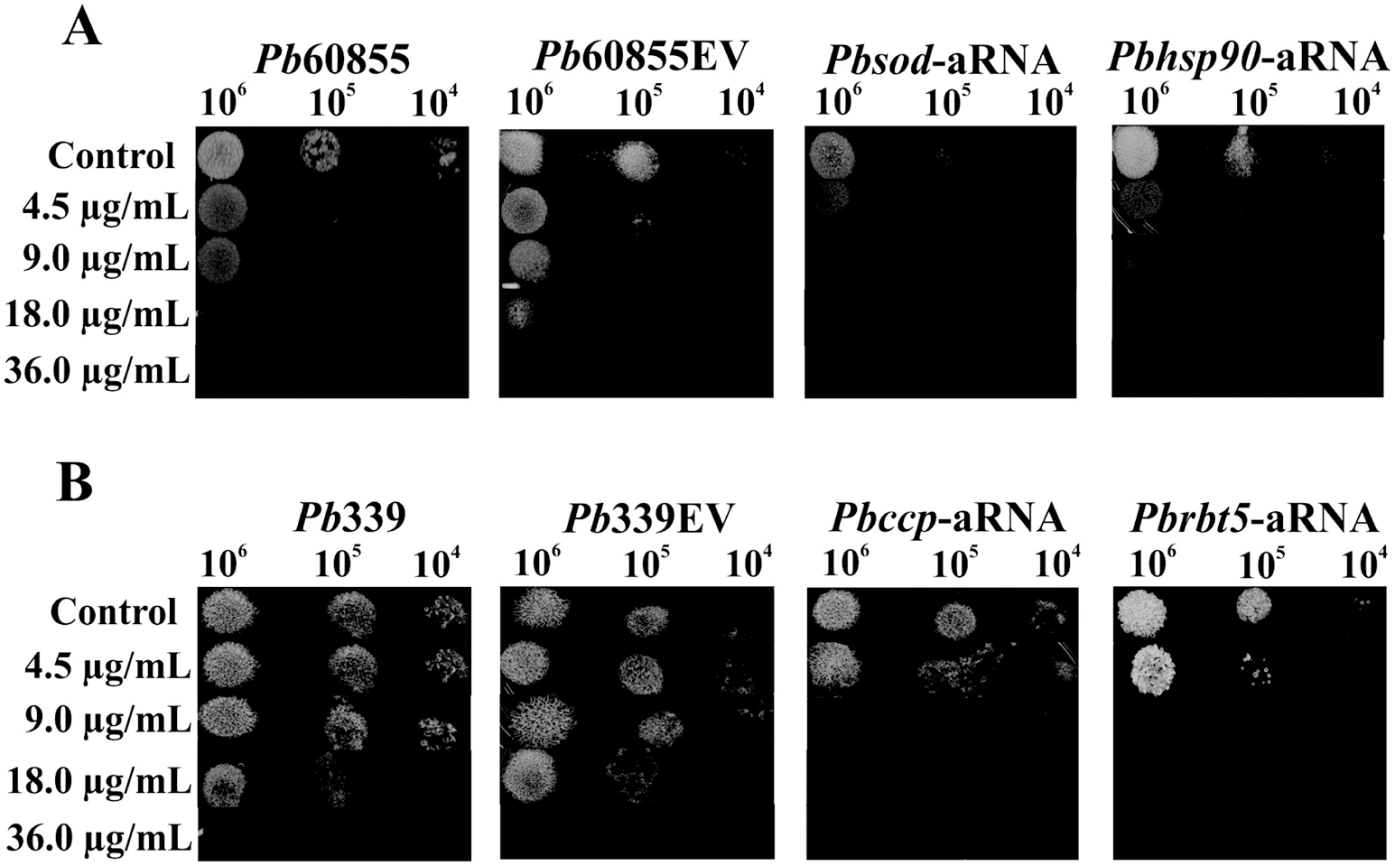
Susceptibility of *Paracoccidioides brasiliensis* yeast cells exposed to argentilactone. Samples containing 10^6^, 10^5^ and 10^4^ *Pbsod*-aRNA, *Pbhsp90*-aRNA **(A)**, *Pbccp*-aRNA and *Pbrbt5*-aRNA **(B)** yeast cells were spotted in solid Fava Netto’s supplemented with argentilactone at the concentrations of 4.5, 9, 18 and 36 μg/mL. Control cells, wild type (WT) and empty vector (EV) were assayed without argentilactone. The plates were incubated for 7 days at 36°C before photo documentation.

The growth of *hsp90*, *sod*, *ccp* and *rbt5* silenced mutants was evaluated in the presence of argentilactone (Fig 5). Due the difficulty in obtaining mutants in *P*. *lutzii*, the mutants used here were obtained to *P*. *brasiliensis* from studies performed previously (14,24,25,26). *Pb*339, *Pb*60855, *Pb*339EV, *Pb*60855EV, *Pbsod*-aRNA, *Pbhsp90*-aRNA, *Pbccp*-aRNA and *Pbrbt5*-aRNA were grown for 6 days in the presence of 4.5, 9, 18 and 36 μg/mL argentilactone. All mutants presented greater sensitivity to argentilactone compared to wild-type cells and cells containing the empty vector. Argentilactone inhibits *Pb*60855 and *Pb*60855EV cells in the presence of 18 μg/mL of the compound, as a dose of 36 μg/mL was necessary to affect the cell growth of *Pb*339 and *Pb*339EV. For the silenced mutants *Pbsod*-aRNA, *Pbhsp90*-aRNA, *Pbccp*-aRNA and *Pbrbt5*-aRNA, 9 μg/mL argentilactone was sufficient to inhibit the growth of the *Pbhsp90*-aRNA-silenced mutant. These results suggest that overexpression of the *hsp90*, *sod*, *ccp* and *rbt5* transcripts are important for the response of *Paracoccidioides* spp. to argentilactone and that the silencing of these genes directly influences the growth of the fungus in the presence of argentilactone.

### Argentilactone promotes the induction of oxidative stress and dysfunction of the mitochondrial membrane potential

**Fig 6:**
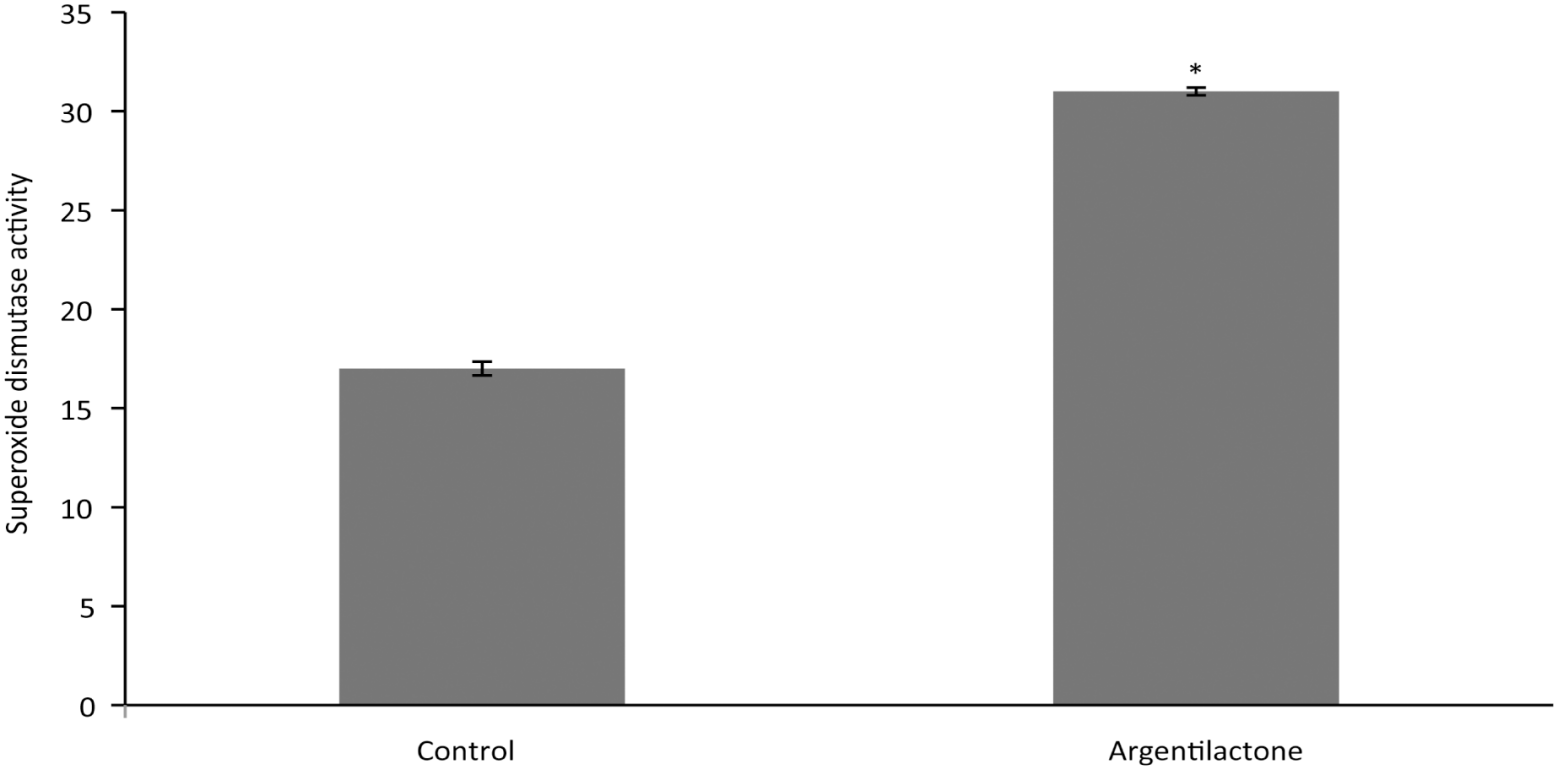
Superoxide dismutase activity. Yeast cells were grown in the presence of argentilactone for 6 h, and total proteins were extracted and used to measure superoxide dismutase activity. Student’s t test was used for statistical comparisons, and the observed differences were statistically significant (*p* ˂ 0.05). The error bars represent the standard deviation of three biological replicates.

Due to the induction of genes related to oxidative stress, SOD enzymatic activity was investigated. The enzymatic activity was measured after growing *P*. *lutzii* for 6 h in the presence or absence of argentilactone. A significant increase of enzymatic activity was observed in the presence of argentilactone (Fig 6).

**Fig 7:**
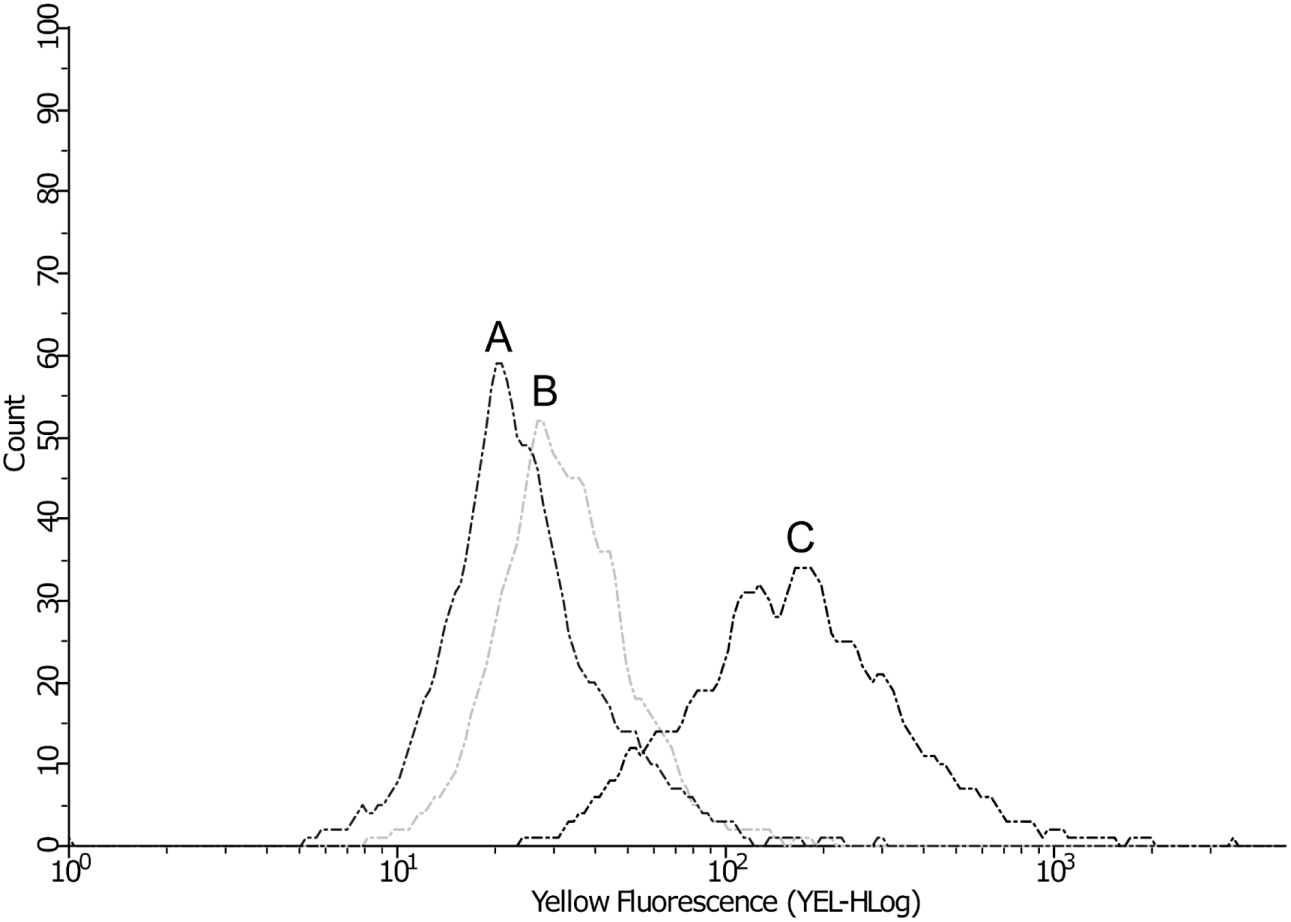
Effect of argentilactone on the mitochondrial membrane potential of *Paracoccidioides lutzii*. The mitochondrial membrane potential was determined by flow cytometry analysis of yeast cells treated with antimycin A (A) or argentilactone (B) for 6 h and stained with rhodamine 123. Control cells without treatment (C) were used in the test.

The ability of argentilactone to cause damage to the mitochondrial membrane potential was compared to antimycin A, a potent inhibitor of the electron transport chain. The mitochondrial membrane was monitored using rhodamine 123, which decreases its fluorescence when there is depolarization of the mitochondrial membrane potential. Flow cytometry data showed that argentilactone exerted a similar effect on cells as antimycin A. In the presence of argentilactone, the intracellular fluorescence due to rhodamine 123 was decreased (Fig 7).

## Discussion

The objective of this study was to analyze the transcriptional profile of *P*. *lutzii* in the presence of argentilactone. The regulation of transcripts and proteins from metabolic pathways by antifungals has been described for several microorganisms, including the antifungal itraconazole, which acts in a similar pathway as ergosterol [37,38,39]. The concentration of argentilactone utilized in the experiments affected global gene expression in *P*. *lutzii*. Here, argentilactone affected the metabolism of *P*. *lutzii*, as alcoholic fermentation was down-regulated, and glycolysis, β-oxidation and the methylcitrate cycle were up-regulated. In addition, argentilactone interfered with urea synthesis and excretion in *P*. *lutzii*. In treatment with argentilactone, the fungus seems to use the amphibian-like uricolytic pathway to excrete and metabolize amide groups. The utilization of different pathways to reach the destination of the amide groups has been reported in organisms. In *Aedes aegypti*, urea synthesis and excretion are regulated by a unique cross-talk mechanism, which is one of the biochemical mechanisms responsible for the success of the infection [40].

Chitin synthases are enzymes that play an important role in cellular integrity, growth and virulence of human fungal pathogens, including *Candida albicans* and *Cryptococcus neoformans* [41,42]. It has been observed that high ergosterol levels can inhibit chitin synthases, whereas *C*. *albicans* mutants with low ergosterol content showed increased levels of chitin synthesis [43]. Here, an ergosterol biosynthesis protein and sterol desaturase transcripts were up-regulated, and several transcripts related to the synthesis of chitin and glucan were down-regulated. In addition, the polymer levels of the cell wall of *P*. *lutzii* were decreased when treated with argentilactone. Studies have demonstrated the importance of genes related to cell wall biosynthesis in *Paracoccidioides* spp., including chitin synthase genes [9, 44–48].

We assumed that central carbon metabolism is inhibited by argentilactone, as we found a profile similar to the effects of pyrazole (an alcohol dehydrogenase inhibitor), which reduced the ethanol yield in *Saccharomyces cerevisiae* [49].

*hsp90*, a molecular chaperone that plays an important role in the assembly and regulation of several signaling systems in eukaryotes [50], is a highly conserved protein and represents about 2% of all cellular proteins in cells [51]. Furthermore, *hsp90* is required for viability under stress conditions in eukaryotes [52] and plays an important role in fungi, including the physiology of *P*. *lutzii* [23], indicating its utility as a potential antifungal target [53,54]. In *P*. *lutzii*, the heat shock response is associated with pathogenesis because the change in temperature is responsible for dimorphic transition, which is essential to establish infection. *hsp90* regulates the proliferation and adaptation of *P*. *lutzii* to different environmental conditions [23,55]. Here, transcriptional and qRT-PCR date showed that *hsp90* was up-regulated in the presence of argentilactone. The plate sensitivity test using the mutant *P*. *brasiliensis Pbhsp90*-aRNA showed that *hsp90* was recruited by *P*. *brasiliensis* growth in the presence of argentilactone.

Many organisms present a ROS defense system generated by aerobic respiration and substrate oxidation [56]. SOD is required for *P*. *lutzii* growth in the presence of argentilactone, as its activity was increased in this condition. Increased levels of SOD activity may represent a primary antioxidant defense against ROS in *P*. *lutzii* [57]. In addition, the *P*. *brasiliensis* mutant *Pbsod*-aRNA exhibited decreased growth in the presence of this compound, as found in *C*. *neoformans* [58]. Argentilactone seems to stimulate oxidative stress in *Paracoccidioides* spp. Therefore, the fungus seems to induce antioxidant enzymes, such as SOD, which act to reduce ROS, aiming to prevent cell damage. The induction of *hsp90* and *sod* suggests that the primary heat shock stress could induce subsequent oxidative stress due to oxygen availability. Similar data have been previously described in *S*. *cerevisiae* [59,60]. Argentilactone seems to be acting on one of the main sources of ROS, the electron transport chain, as several transcripts related to electron transport, such as NADPH-adrenodoxin oxidoreductase, NADH-cytochrome b5 reductase and the indirectly related *ccp*, were up-regulated.

Peroxidases are enzymes that use various electron donors to reduce H_2_O_2_ to H_2_O. In *C*. *neoformans*, the growth of a *ccp* mutant was affected by the presence of H_2_O_2_ [58]. CCP catalyzes the reduction of hydrogen peroxide using cytochrome c as an electron donor. In *S*. *cerevisiae*, the increased expression of *ccp* seems to be caused by an increase of ROS produced during aerobic growth, thus confirming the biological role of this enzyme in cellular detoxification and the elimination of hydrogen peroxide [60]. We suggest here that the cellular oxidative stress caused by argentilactone results in an overexpression of *sod*, which is responsible for catalyzing the dismutation of superoxide into H_2_O_2_. The presence of H_2_O_2_ would induce the increased expression of *ccp* in an attempt to reduce hydrogen peroxide. Here, the increase in the *ccp* transcript level demonstrated in the transcriptional and qRT-PCR data and the decreased growth of *Paracoccidioides Pbrbt5-*aRNA in the presence of argentilactone show that *ccp* is required for the fungus in this condition.

ROS are universal products of aerobic metabolism, which can also be produced under stressful conditions. In eukaryotic cells, mitochondria are the main source of ROS [61]. Changes in the proton gradient resulting from the inhibition of electron transport can result in the production of reactive oxygen species (ROS) [61,62]. Thus, we assumed that argentilactone could have been acting on mitochondria, and our results confirmed this hypothesis.

In *Coccidioides immitis* [63] and *S*. *cerevisiae* [64], RBT5 is an antigenic protein anchored to the cell wall. *rbt5* is important for the morphogenesis of the cell wall of *C*. *albicans* [65] and *Coccidioides* during infection [66]. In *P*. *lutzii*, *rbt5* is a major surface antigen [67]. Here, the increase in the *rbt5* transcript level exhibited in the transcriptional and qRT-PCR data and the decrease of *Pbrbt5*-aRNA growth in the presence of argentilactone suggest that *rbt5* is required for the fungus in this condition. *rbt5* could be increased in the presence of argentilactone in an attempt to maintain the morphogenesis of the cell wall, as several transcripts related to the biosynthesis of the cell wall, mainly to chitin biosynthesis, were down-regulated. Fluorescence microscopy data showing that the level of chitin and glucan polymers in the cell wall of *P*. *lutzii* yeast cells was lower in the presence than in the absence of argentilactone corroborate these results. Genes important to the maintenance and integrity of the cell wall were described to be down-regulated in fungi exposed to amphotericin B [68,69] or to investigatory antifungals, such as artemisinin [70] or oenothein B, an anti-*Paracoccidioides* [28]. In *Aspergillus niger* and *S*. *cerevisiae*, the transcription factor RlmA is required for the up-regulation of cell wall stress-induced genes [71] leading to increased chitin content. Here, RlmA is down-regulated, corroborating the down-regulation of genes related to chitin synthesis.

The considerable number of transcription factors regulated in the presence of argentilactone suggests a complex regulatory mechanism. Conversely, the high percentage of unclassified proteins indicates that additional studies must be performed to elucidate the mode of action of argentilactone on *Paracoccidioides* spp.

One of the limitations of the study was that all experiments were not performed on *P*. *lutzii* and *P*. *brasiliensis* due to the difficulty in obtaining mutants of *P*. *lutzii*. This analysis should be attempted in the future.

### Conclusion

Argentilactone seems to be able to penetrate into *Paracoccidioides* spp. yeast cells and modulate cellular targets. Argentilactone seems to induce oxidative stress and interfere with the biosynthesis of the *Paracoccidioides* spp. cell wall. Given the overall stress caused by argentilactone in *Paracoccidioides* spp., other studies must be performed to better elucidate the mode of action of argentilactone in *Paracoccidioides* spp.

## Supporting Information

S1 Fig Global analysis of RNAseq data. Mapped reads were analyzed using the DEGseq package and plotting graphs were obtained. The transcripts are represented by dots. **(A)** Scatter plot shows the number of reads (log_2_) counts for each transcript P-Al (Presence of Argentilactone) and A-Al (Absence of Argentilactone) conditions. **(B)** MA-plot of P-Al *versus* A-Al conditions shows the intensity of the expression of identified transcripts (log_2_ of fold change) in the *y* axis [M] and the read counts (log_2_) for each transcript in the *x* axis [A]. In addition, the graph shows the number of differentially expressed transcripts obtained from FET (Fisher’s Exact Test) using a *p*-value of 0.001, as indicated in red.
(TIF)

S2 Fig Effect of argentilactone on transcript expression levels in *Paracoccidioides brasiliensis* yeast cells. The expression levels of *hsp90*, *ccp*, *sod* and *rbt5* genes in *Paracoccidoides brasiliensis*. yeast cells grown in MMcM liquid medium with or without argentilactone were analyzed. The data were normalized using the constitutive gene encoding α-tubulin as the endogenous control and are presented as relative expression in comparison to the experimental control cells, whose value was set to 1. Data are expressed as the mean ± standard deviation of the triplicates of independent experiments. *, significantly different from the control at a *p-*value of ˂ 0.05.
(TIF)

S1 Table Gene-specific primers used for qRT-PCR assays. (DOCX)

S2 Table Functional classification of up-regulated genes from *Paracoccidioides lutzii* yeast cells in the presence argentilactone. (DOCX)

S3 Table Functional classification of down-regulated genes from *Paracoccidioides lutzii* yeast cells in the presence argentilactone. (DOCX)
