# Supplementary figures and images for: Effects of Argentilactone on the Transcriptional Profile, Cell Wall and Oxidative Stress of Paracoccidioides spp
Source: PLoS Negl Trop Dis. 2016 Jan 6;10(1):e0004309. doi: 10.1371/journal.pntd.0004309 (PMC4703379; doi:10.1371/journal.pntd.0004309)

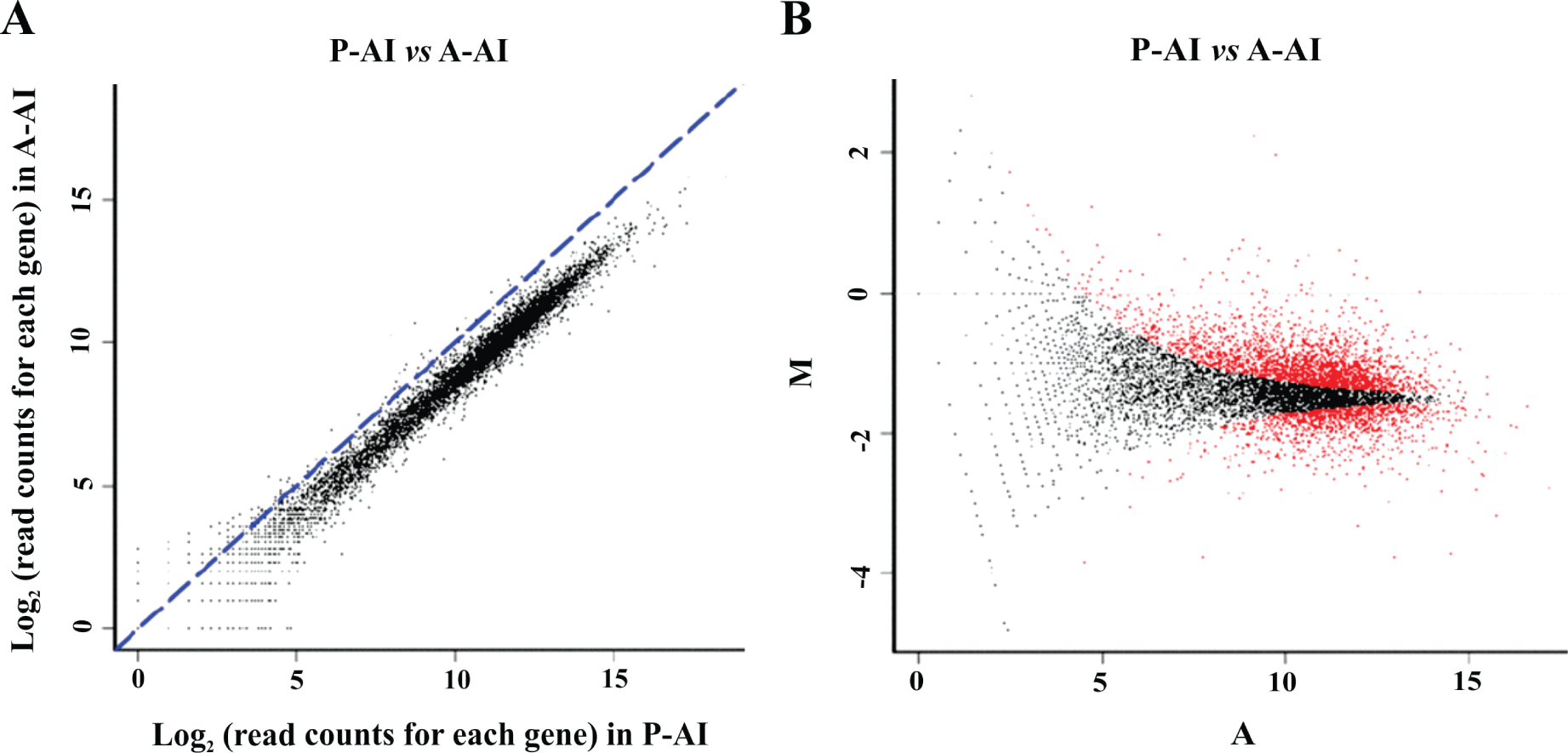

Supplement: S1 Fig — Mapped reads were analyzed using the DEGseq package and plotting graphs were obtained. The transcripts are represented by dots. (A) Scatter plot shows the number of reads (log2) counts for each transcript P-Al (Presence of Argentilactone) and A-Al (Absence of Argentilactone) conditions. (B) MA-plot of P-Al versus A-Al conditions shows the intensity of the expression of identified transcripts (log2 of fold change) in the y axis [M] and the read counts (log2) for each transcript in the x axis [A]. In addition, the graph shows the number of differentially expressed transcripts obtained from FET (Fisher’s Exact Test) using a p-value of 0.001, as indicated in red. (TIF) [file pntd.0004309.s001.tif]

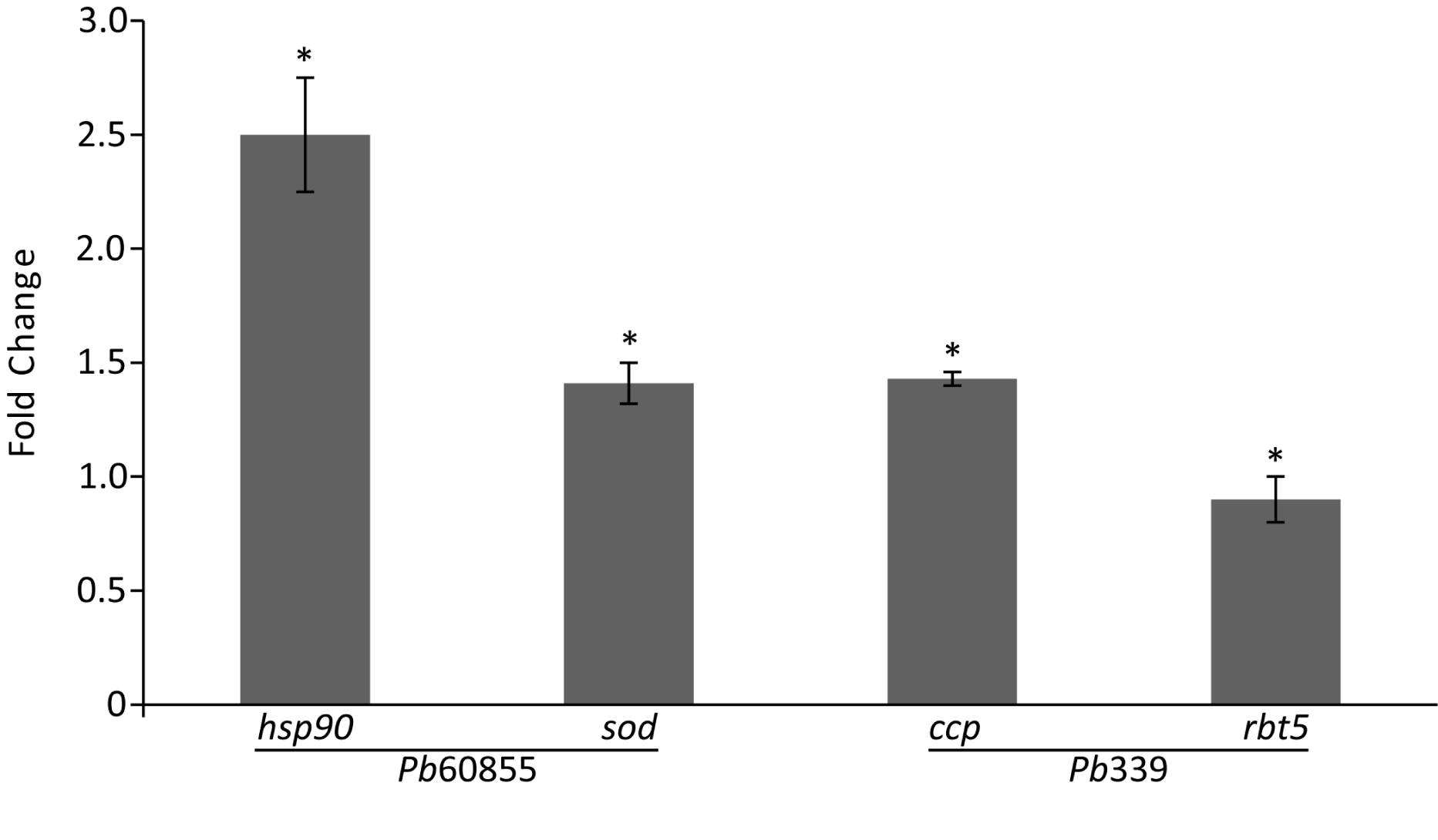

Supplement: S2 Fig — The expression levels of hsp90, ccp, sod and rbt5 genes in Paracoccidoides brasiliensis. yeast cells grown in MMcM liquid medium with or without argentilactone were analyzed. The data were normalized using the constitutive gene encoding α-tubulin as the endogenous control and are presented as relative expression in comparison to the experimental control cells, whose value was set to 1. Data are expressed as the mean ± standard deviation of the triplicates of independent experiments. *, significantly different from the control at a p-value of ˂ 0.05. (TIF) [file pntd.0004309.s002.tif]
