# Supplementary material for: Effects of Argentilactone on the Transcriptional Profile, Cell Wall and Oxidative Stress of Paracoccidioides spp
Source: PLoS Negl Trop Dis. 2016 Jan 6;10(1):e0004309. doi: 10.1371/journal.pntd.0004309 (PMC4703379; doi:10.1371/journal.pntd.0004309)
Supplement: S1 Table — (DOCX) [file pntd.0004309.s003.docx]

**Supplementary Table 1**– Gene-specific primers used for qRT-PCR assays.

| **Sequence Name** | **Forward primer (5’-3’)** | **Reverse primer (5’-3’)** | **Amplicon size (bp)** |
| --- | --- | --- | --- |
| Superoxide dismutase (*sod*) | ACTGCGCAAGTTATGATGGAA | CACGGGAAGGGTCCATTTTC | 141 |
| Heat Shock Protein 90 (*hsp 90*) | CAATTAGAGTATGAAGGTAAGAC | GTTCGGTAATCAAAGCAGCAC | 132 |
| Cytochrome c peroxidase (*ccp*) | GGTAGCTATGGACCGGGTTCT | CTCTCGCAGCTTTCAAACCA | 138 |
| Hemoglobin ligant RBT5 (*rbt5*) | ATATCCCACCTTGCGCTTTGA | GGGCAGCAACGTCGCAAGA | 130 |
