# Supplementary material for: Effects of Argentilactone on the Transcriptional Profile, Cell Wall and Oxidative Stress of Paracoccidioides spp
Source: PLoS Negl Trop Dis. 2016 Jan 6;10(1):e0004309. doi: 10.1371/journal.pntd.0004309 (PMC4703379; doi:10.1371/journal.pntd.0004309)
Supplement: S2 Table — (DOCX) [file pntd.0004309.s004.docx]

**Supplementary Table 2:** Functional classification of up-regulated genes from *Paracoccidioides* yeast cells in the presence argentilactone.

| **Accession number/ Functional classification** | **Gene product** | ***P-*value** | **Annotated function** | **EC number** | **Fold Change(Log_2_)** |
| --- | --- | --- | --- | --- | --- |
|  |  |  |  |  |  |
| ***METABOLISM*** |  |  |  |  |  |
|  |  |  |  |  |  |
| **Amino acid metabolism** |  |  |  |  |  |
|  |  |  |  |  |  |
| PAAG_08664.2 | Phenylacetate 2-hydroxylase | 9.09e-120 | Metabolism of phenylpropanoids - Phenylalanine metabolism | 1.14.13.-. | 0.648015 |
| PAAG_04555.2 | Sarcosine oxidase | 0 | Degradation of lysine | 1.5.3.1 | 1.82018 |
| PAAG_05776.2 | Dihydroxy-acid dehydratase | 1.65e-104 | Biosynthesis of valine | 4.2.1.9 | 0.656284 |
| PAAG_01527.2 | 3-isopropylmalate dehydrogenase | 2.74e-24 | Valine, leucine and isoleucine biosynthesis | 1.1.1.85 | 0.629139 |
| PAAG_06387.2 | Homoisocitrate dehydrogenase | 0 | Biosynthesis of isoleucine / tricarboxylic-acid pathway (citrate cycle, Krebs cycle, TCA cycle) | 1.1.1.87 | 1.31472 |
| PAAG_05405.2 | Histidinol dehydrogenase | 9.45e-16 | Histidine metabolism | 1.1.1.23 | 0.724833 |
| PAAG_03845.2 | Succinate-semialdehyde dehydrogenase | 4.84e-23 | Metabolism of secondary products derived from L-glutamic acid, L-proline and L-ornithine | 1.2.1.24 | 0.928845 |
| PAAG_01310.2 | 2-oxoisovalerate dehydrogenase subunit α, mitochondrial | 4.57e-66 | Degradation of leucine | 1.2.4.4 | 0.758558 |
| PAAG_02509.2 | 2-amino-3-carboxymuconate-6-semialdehyde decarboxylase | 6.61e-08 | Tryptophan metabolism | 4.1.1.45 | 0.838038 |
| PAAG_01437.2 | Uricase | 8.96e-238 | Purine catabolism, degradation of hypoxanthine and xanthine to urea via urate (alternate path, xanthine dehydrogenase) | 1.7.3.3 | 0.869608 |
| PAAG_02604.2 | L-tyrosine decarboxylase | 4.67e-313 | [Degradation of tyrosine](http://mips.gsf.de/cgi-bin/proj/funcatDB/search_advanced.pl?action=2&wert=01.01.09.05.02) | 4.1.1.25 | 1.24892 |
| PAAG_08164.2 | Homogentisate 1,2-dioxygenase | 9.04e-138 | Metabolism of tyrosine | 1.13.11.5 | 0.786151 |
| PAAG_05352.2 | Flavoprotein oxygenase | 9.11e-62 | Pyrimidine metabolism | 1.5.1.- | 0.851756 |
| PAAG_02603.2 | Aspartate aminotransferase | 1.16e-136 | Assimilation of ammonia, metabolism of the glutamate group | 2.6.1.1 | 0.603955 |
| PAAG_06404.2 | Aspartate aminotransferase | 3.35e-33 | Assimilation of ammonia, metabolism of the glutamate group | 2.6.1.1 | 0.597749 |
| PAAG_01419.2 | Kynureninase | 1.49e-65 | Metabolism of tryptophan | 3.7.1.3 | 0.608505 |
| PAAG_07428.2 | Allantoinase | 2.54e-39 | Metabolism of urea (urea cycle) | 3.5.2.5 | 0.588684 |
| PAAG_06215.2 | Hydroxymethylglutaryl-CoA lyase | 9.89e-35 | Metabolism of the pyruvate family (alanine, isoleucine, leucine, valine) and D-alanine | 4.1.3.4 | 0.628216 |
| PAAG_05355.2 | Acetamidase | 4.94e-16 | Degradation of L-phenylalanine to phenylacetate via 2-phenylacetamide | 3.5.1.4 | 0.993709 |
| PAAG_00869.2 | Fumarylacetoacetate hydrolase domain-containing protein | 2.24e-85 | Degradation of phenylalanine | 3.7.-.-. | 0.710955 |
|  |  |  |  |  |  |
| **Metabolism of vitamins** |  |  |  |  |  |
|  |  |  |  |  |  |
| PAAG_08856.2 | Nicotinate-nucleotide pyrophosphorylase | 3.40e-122 | Biosynthesis of vitamins, cofactors, and prosthetic groups-Nicotinate and nicotinamide metabolism | 2.4.2.19 | 0.766909 |
| PAAG_03629.2 | Xanthine dehydrogenase | 2.61e-119 | Biosynthesis of vitamins, cofactors, and prosthetic groups - Purine degradation, xanthine | 1.17.1.4 | 0.701249 |
| PAAG_03944.2 | Cytosolic Fe-S cluster assembling factor NBP35 | 3.63e-116 | Regulation of the metabolism of vitamins, cofactors, and prosthetic group | - | 0.965167 |
| PAAG_03857.2 | Phosphopantothenate-cysteine ligase | 9.12e-42 | Biosynthesis of vitamins, cofactors, and prosthetic groups | 6.3.2.5 | 0.624918 |
| PAAG_07489.2 | *P*-hydroxybenzoate-polyprenyltransferase | 9.39e-07 | Biosynthesis of vitamins, cofactors, and prosthetic groups - Ubiquinone and other terpenoid-quinone biosynthesis | 2.5.1.39 | 0.733221 |
| PAAG_05944.2 | Dihydrofolate reductase | 5.07e-07 | Metabolism of cofactors and vitamins - Folate biosynthesis | 1.5.1.3 | 0.751069 |
|  |  |  |  |  |  |
| **Lipid metabolism** |  |  |  |  |  |
|  |  |  |  |  |  |
| PAAG_03696.2 | Ergosterol biosynthesis protein | 2.59e-197 | Tetracyclic and pentacyclic triterpenes (cholesterin, steroids and hopanoids) metabolism | - | 0.712178 |
| PAAG_08859.2 | Peroxisomal multifunctional enzyme | 0 | oxidation of fatty acids | - | 0.84671 |
| PAAG_02984.2 | Endopolyphosphatase | 5.12e-209 | Lipid, fatty acid and isoprenoid metabolism | 3.6.1.10 | 0.901263 |
| PAAG_05416.2 | NADP-dependent leukotriene B4 12-hydroxydehydrogenase | 2.52e-151 | Lipid, fatty acid and isoprenoid metabolism | 1.3.1.74 | 1.16061 |
| PAAG_03631.2 | 12-oxophytodienoate reductase | 3.56e-73 | Lipid, fatty acid and isoprenoid metabolism | 1.3.1.42 | 0.721242 |
| PAAG_04963.2 | Oleate-induced peroxisomal protein | 4.06e-57 | Lipid, fatty acid and isoprenoid metabolism | - | 0.70642 |
| PAAG_04856.2 | Acetyl-CoA hydrolase | 1.99e-19 | Regulation of lipid, fatty acid and isoprenoid metabolism | 3.1.2.1 | 0.977092 |
| PAAG_03689.2 | 3-ketoacyl-CoA thiolase B | 3.38e-96 | Fatty acid beta-oxidation | 2.3.1.16 | 0.709643 |
| PAAG_06309.2 | Enoyl-CoA hydratase - b-oxidação | 1.99e-129 | fatty acid beta-oxidation | 4.2.1.17 | 0.777447 |
| PAAG_06392.2 | Enoyl-CoA hydratase/isomerase | 3.71e-26 | fatty acid beta-oxidation | 4.2.1.17 | 0.64653 |
| PAAG_07013.2 | Enoyl-CoA hydratase/carnithine racemase | 1.25e-25 | fatty acid beta-oxidation | 4.2.1.17 | 0.61983 |
| PAAG_07793.2 | Enoyl-CoA hydratase | 7.57e-15 | fatty acid beta-oxidation | 4.2.1.17 | 0.903836 |
| PAAG_00435.2 | Acyl-CoA dehydrogenase | 1.72e-72 | fatty acid beta-oxidation | 1.3.99.3 | 0.692312 |
| PAAG_03020.2 | Sterol desaturase | 4.01e-38 | Steroid biosynthesis | 1.14.21.6 | 1.02641 |
|  |  |  |  |  |  |
| **Carbohydrate metabolism** |  |  |  |  |  |
|  |  |  |  |  |  |
| PAAG_06817.2 | UTP-glucose-1-phosphate uridylyltransferase | 2.12e-195 | Sugar, glucoside, polyol and carboxylate catabolism - Galactose metabolism | 2.7.7.9 | 0.605377 |
| PAAG_06103.2 | Succinate dehydrogenase iron-sulfur subunit | 1.69e-106 | C-compound and carbohydrate metabolism - Citrate cycle, second carbon oxidation | 1.3.5.1 | 0.801342 |
| PAAG_02990.2 | β-glucosidase | 1.15e-72 | Polysaccharide metabolism - Starch and sucrose metabolism | 3.2.1.21 | 0.831532 |
| PAAG_00280.2 | Rhizobactin siderophore biosynthesis protein rhbD | 1.84e-27 | C-compound and carbohydrate metabolism | 2.3.1.82 | 0.61462 |
| PAAG_05254.2 | α-galactosidase | 0 | Aminosaccharide metabolismo | 3.2.1.22 | 0.641133 |
| PAAG_02874.2 | α/β hydrolase | 5.34e-35 | C-compound and carbohydrate metabolism | - | 1.05492 |
|  |  |  |  |  |  |
| ***BIOGENESIS OF CELLULAR COMPONENTS*** |  |  |  |  |  |
|  |  |  |  |  |  |
| **Fungal-type cell wall biogenesis** |  |  |  |  |  |
|  |  |  |  |  |  |
| PAAG_03887.2 | GTP-binding protein RHO4 | 2.83e-32 | cell wall organization | - | 0.877355 |
| PAAG_07670.2 | Cell wall protein ECM33 precursor | 7.82e-30 | Fungal-type cell wall organization | - | 0.859322 |
|  |  |  |  |  |  |
| ***TRANSCRIPTION*** |  |  |  |  |  |
|  |  |  |  |  |  |
| **RNA synthesis** |  |  |  |  |  |
|  |  |  |  |  |  |
| PAAG_02902.2 | Transcription factor fet5 | 2.19e-19 | transcriptional control | - | 0.642347 |
| PAAG_01359.2 | C6 transcription factor CTF1B | 0 | Transcription initiation | - | 2.27924 |
| PAAG_06537.2 | C2H2 transcription factor | 6.28e-66 | RNA synthesis | - | 0.826038 |
| PAAG_04637.2 | Pre-mRNA-splicing factor rse1 | 1.01e-228 | mRNA synthesis | - | 0.605479 |
| PAAG_04726.2 | Pirin | 0 | mRNA synthesis | 1.13.11.24 | 0.765106 |
| PAAG_05900.2 | CHCH domain-containing protein | 0 | Transcription repression | - | 0.697087 |
| PAAG_06766.2 | Protein cgi121 | 1.61e-39 | Positive regulation of transcription from RNA polymerase II promoter | - | 0.844316 |
|  |  |  |  |  |  |
| ***CELL RESCUE, DEFENSE AND VIRULENCE*** |  |  |  |  |  |
|  |  |  |  |  |  |
| **Stress Response** |  |  |  |  |  |
|  |  |  |  |  |  |
| PAAG_03334.2 | Peptidyl-prolyl cis-trans isomerase D | 0 | Heat shock response - Protein folding catalysts | 5.2.1.8 | 1.07501 |
| PAAG_02417.2 | ATP-dependent protease La 2 | 2.79e-138 | Temperature perception and response | 3.4.21.53 | 0.729878 |
| PAAG_07335.2 | leptomycin B resistance protein pmd1 | 3.04e-117 | Chemoperception and response - Membrane transport/ABC transporters | - | 0.713303 |
| PAAG_02725.2 | Superoxide dismutase | 6.58e-69 | Oxidative stress response - Acting on superoxide as acceptor | 1.15.1.1 | 0.659711 |
| PAAG_03502.2 | Cytochrome c peroxidase | 6.65e-22 | Oxidative stress response - Acting on a peroxide as acceptor | 1.11.1.5 | 0.921105 |
| PAAG_00549.2 | Acyl-Coenzyme A dehydrogenase | 9.65e-13 | Oxidative stress response - superoxide metabolism | 3.3.2.9 | 0.598807 |
| PAAG_07445.2 | Actin cortical patch protein Sur7 | 1.85e-36 | Response to drug | - | 0.760738 |
| PAAG_05158.2 | Hemoglobin ligant RBT5 | 3.89e-48 | Stress response | - | 2.26805 |
| PAAG_02130.2 | Hsp98 | 0 | Heat shock response | 3.4.21.- | 1.29705 |
| PAAG_05226.2 | Hsp90 binding co-chaperone SBA1 | 0 | Stress response | - | 0.861654 |
| PAAG_05679.2 | Hsp90 family chaperone HSP82 | 0 | Stress response: response to drug | - | 1.27378 |
| PAAG_02686.2 | Hsp90 co-chaperone AHA1 | 5.86e-236 | Stress response | - | 0.759609 |
| PAAG_07750.2 | Hsp88 | 0 | Temperature perception and response | - | 0.911373 |
| PAAG_08003.2 | Hsp70 | 0 | Stress response - Folding, sorting and degradation | - | 1.10419 |
| PAAG_03533.2 | Hsp70 nucleotide exchange factor fes1 | 6.09e-35 | ATP binding | - | 0.796155 |
| PAAG_00871.2 | Hsp30 | 0 | Oxidative stress response | - | 1.67511 |
| PAAG_05142.2 | Hsp10, mitochondrial | 0 | Heat shock response | - | 0.645147 |
| PAAG_06811.2 | Hsp STI1 | 0 | Unfolded protein response (e.g. ER quality control) | - | 0.760116 |
| PAAG_01339.2 | Hsp SSC1 | 7.20e-125 | Temperature perception and response | - | 0.657635 |
| PAAG_03106.2 | ThiJ/PfpI family protein | 8.45e-213 | Unfolded protein response (e.g. ER quality control) | - | 0.85776 |
| PAAG_07938.2 | ATP-binding cassette sub-family G member 5 | 1.80e-63 | Perception of nutrients and nutritional adaptation | - | 0.955309 |
| PAAG_00478.2 | DnaJ domain protein Psi | 3.31e-98 | Unfolded protein response (e.g. ER quality control) | - | 0.611722 |
|  |  |  |  |  |  |
| **Detoxification** |  |  |  |  |  |
|  |  |  |  |  |  |
| PAAG_00635.2 | ABC transporter CDR4 | 0 | Detoxification by export | - | 0.977797 |
| PAAG_06017.2 | Pleiotropic ABC efflux transporter of multiple drugs | 1.21e-197 | Drug/toxin transport | - | 0.995875 |
| PAAG_06133.2 | MFS multidrug transporter | 3.68e-118 | Detoxification by export | - | 0.699801 |
| PAAG_00178.2 | Monooxygenase | 1.54e-37 | Detoxification | 1.13.-.-. | 1.34872 |
|  |  |  |  |  |  |
| ***CELLULAR TRANSPORT, TRANSPORT FACILITIES AND TRANSPORT ROUTES*** |  |  |  |  |  |
|  |  |  |  |  |  |
| **Transported compounds (substrates)** |  |  |  |  |  |
|  |  |  |  |  |  |
| PAAG_07824.2 | Solute carrier family 35 member F2 | 1.53e-48 | Sugar transport | - | 0.715919 |
| PAAG_04313.2 | Fatty acid transporter protein | 5.49e-16 | Lipid/fatty acid transport | 6.2.1.-. | 0.597778 |
| PAAG_07154.2 | Copper-transporting P-type ATPase | 7.83e-316 | Heavy metal ion transport (Cu+, Fe3+, etc.) | 3.6.3.4 | 1.07045 |
| PAAG_06668.2 | Mitochondrial metallochaperone Sco1 | 0 | heavy metal ion transport (Cu+, Fe3+, etc.) | - | 1.02123 |
|  |  |  |  |  |  |
| **Transport routes** |  |  |  |  |  |
|  |  |  |  |  |  |
| PAAG_00797.2 | Mitochondrial protein import protein MAS5 | 1.49e-192 | mitochondrial transport | - | 0.616346 |
| PAAG_05225.2 | Vacuolar protein sorting protein | 2.30e-94 | Vacuolar/lysosomal transport | - | 1.08656 |
| PAAG_05960.2 | NIPSNAP family protein | 0 | Vesicular transport (Golgi network, etc.) | - | 0.66891 |
|  |  |  |  |  |  |
| ***ENERGY*** |  |  |  |  |  |
|  |  |  |  |  |  |
| **Glycolysis and Gluconeogenesis** |  |  |  |  |  |
|  |  |  |  |  |  |
| PAAG_02189.2 | Class II aldolase | 8.32e-132 | Glycolysis and gluconeogenesis | - | 0.747335 |
| PAAG_08468.2 | Glyceraldehyde-3-phosphate dehydrogenase | 0 | Glycolysis and gluconeogenesis | 1.2.1.12 | 0.791926 |
| PAAG_08203.2 | Phosphoenolpyruvate carboxykinase | 0 | Glycolysis and gluconeogenesis | 4.1.1.49 | 0.750147 |
|  |  |  |  |  |  |
| **Tricarboxylic-acid pathway (citrate cycle, Krebs cycle, TCA cycle)** |  |  |  |  |  |
|  |  |  |  |  |  |
| PAAG_02732.2 | 2-oxoglutarate dehydrogenase E1 | 0 | C-compound and carbohydrate metabolism / tricarboxylic-acid pathway (citrate cycle, Krebs cycle, TCA cycle) | 1.2.4.2 | 0.679196 |
|  |  |  |  |  |  |
| **Respiration** |  |  |  |  |  |
|  |  |  |  |  |  |
| PAAG_01902.2 | Electron transfer flavoprotein-ubiquinone oxidoreductase | 1.75e-74 | Aerobic respiration | 1.5.5.1 | 0.754759 |
|  |  |  |  |  |  |
| **Electron transport and membrane-associated energy conservation** |  |  |  |  |  |
|  |  |  |  |  |  |
| PAAG_08082.2 | Plasma membrane ATPase | 1.94e-250 | Electron transport and membrane-associated energy conservation - Ca2+ transporting ATPase | 3.6.3.8 | 0.790341 |
| PAAG_06230.2 | NADPH-adrenodoxin oxidoreductase | 6.46e-20 | Accessory proteins of electron transport and membrane-associated energy conservation | 1.18.1.2 | 0.602664 |
| PAAG_01378.2 | Cytochrome P450 52A3 | 4.50e-62 | Electron transport | 1.14.14.-. | 0.963983 |
| PAAG_01368.2 | Benzoate 4-monooxygenase cytochrome P450 | 4.00e-19 | Electron transport | 1.14.13.12 | 0.767319 |
| PAAG_07984.2 | Cytochrome b2 | 9.39e-24 | Electron transport | 1.1.2.3 | 0.721097 |
| PAAG_04232.2 | Succinate dehydrogenase cytochrome B subunit | 1.08e-93 | Accessory proteins of electron transport and membrane-associated energy conservation | 1.3.99.1 | 0.691527 |
| PAAG_00151.2 | NADH-cytochrome b5 reductase | 2.51e-176 | Electron transport and membrane-associated energy conservation | 1.6.2.2 | 0.739539 |
|  |  |  |  |  |  |
| **Methyl citrate cycle** |  |  |  |  |  |
|  |  |  |  |  |  |
| PAAG_04559.2 | 2-methylcitrate dehydratase | 0 | [C-3 compound catabolism](http://mips.gsf.de/cgi-bin/proj/funcatDB/search_advanced.pl?action=2&wert=01.05.07.07) | 4.2.1.79 | 0.704248 |
|  |  |  |  |  |  |
| ***CELLULAR COMUNICATION/***  ***SIGNAL TRANSDUCTION MECHANISM*** |  |  |  |  |  |
|  |  |  |  |  |  |
| **Cellular signalling** |  |  |  |  |  |
| PAAG_02973.2 | Diploid state maintenance protein chpA | 2.16e-199 | cellular signalling | - | 1.00442 |
| PAAG_00121.2 | Two-component system protein A | 4.22e-147 | cellular signalling | - | 0.821237 |
|  |  |  |  |  |  |
| ***CELL CYCLE AND DNA PROCESSING*** |  |  |  |  |  |
|  |  |  |  |  |  |
| **DNA processing** |  |  |  |  |  |
|  |  |  |  |  |  |
| PAAG_03242.2 | ATPase involved in DNA replication initiation | 4.23e-08 | DNA replication initiation | - | 0.796339 |
|  |  |  |  |  |  |
| **Cell cycle.2** |  |  |  |  |  |
|  |  |  |  |  |  |
| PAAG_02460.2 | Histone-lysine N-methyltransferase | 6.28e-109 | mitotic cell cycle and cell cycle control | 2.1.1.43 | 0.846034 |
| PAAG_04505.2 | G2-specific protein kinase nimA | 2.39e-26 | G2 phase of mitotic cell cycle | 2.7.11.-. | 0.79389 |
| PAAG_08991.2 | Serine/threonine-protein kinase | 1.31e-46 | Mitotic cell cycle - Centromeric chromatin formation proteins | 2.7.11.-. | 0.869402 |
| PAAG_04636.2 | Deubiquitination-protection protein dph1 | 0 | Spindle pole body/centrosome and microtubule cycle | - | 0.951419 |
| PAAG_00320.2 | Kinetochore protein spc25 | 6.27e-36 | Cell cycle - Centromeric chromatin formation proteins | - | 0.738648 |
| PAAG_00513.2 | Cell division control protein | 1.15e-19 | Cell division control | 2.7.11.-. | 0.746852 |
| PAAG_06632.2 | Microtubule associated protein | 9.04e-32 | Cytokinesis (cell division) /septum formation and hydrolysis | 2.7.11.24 | 0.735253 |
| PAAG_00921.2 | Calmodulin-binding protein Sha1 | 1.88e-115 | Mitotic cell cycle and cell cycle control | - | 1.08228 |
| PAAG_04306.2 | Cytokinesis protein Cyk3 | 7.65e-22 | Cytokinesis (cell division) /septum formation and hydrolysis | - | 0.812017 |
| PAAG_06774.2 | Kinesin heavy chain | 5.02e-21 | Spindle pole body/centrosome and microtubule cycle | - | 0.59833 |
| PAAG_06698.2 | Condensin complex component cnd2 | 1.50e-131 | Organization of chromosome structure | - | 0.773326 |
| PAAG_01897.2 | HEC/Ndc80p family protein | 1.19e-134 | Cytokinesis (cell division) /septum formation and hydrolysis | - | 0.754414 |
|  |  |  |  |  |  |
| ***CELL FATE*** |  |  |  |  |  |
|  |  |  |  |  |  |
| **Cell growth/ Morphogenesis** |  |  |  |  |  |
|  |  |  |  |  |  |
| PAAG_01891.2 | SH3 domain-containing protein | 1.21e-31 | Regulation of directional cell growth | - | 0.586314 |
|  |  |  |  |  |  |
| ***CELL TYPE DIFFERENTIATION*** |  |  |  |  |  |
|  |  |  |  |  |  |
| **Fungal/microorganismic cell type differentiation** |  |  |  |  |  |
|  |  |  |  |  |  |
| PAAG_04089.2 | Woronin body major protein | 0 | Fungal and other eukaryotic cell type differentiation | - | 1.00516 |
| PAAG_07514.2 | GTP binding protein Bud4 | 9.72e-111 | Budding, cell polarity and filament formation | - | 0.741454 |
|  |  |  |  |  |  |
| ***PROTEIN SYNTHESIS*** |  |  |  |  |  |
|  |  |  |  |  |  |
| **Ribosome biogenesis** |  |  |  |  |  |
|  |  |  |  |  |  |
| PAAG_03805.2 | Constituent of 66S pre-ribosomal particles | 1.43e-24 | Ribosome biogenesis | - | 0.645115 |
| PAAG_02825.2 | 37S ribosomal protein S11 | 6.59e-199 | Ribosomal proteins | - | 0.837352 |
| PAAG_04886.2 | Ribosome assembly protein SQT1 | 1.51e-95 | ribosomal proteins | - | 0.616549 |
|  |  |  |  |  |  |
| **Translation** |  |  |  |  |  |
|  |  |  |  |  |  |
| PAAG_05882.2 | Translation factor SUI1 | 0 | Translation initiation | - | 1.04363 |
|  |  |  |  |  |  |
| **Aminoacyl-tRNA-synthetases** |  |  |  |  |  |
|  |  |  |  |  |  |
| PAAG_05664.2 | Aspartyl-tRNA synthetase | 0 | Aminoacyl-tRNA-synthetases | 6.1.1.12 | 1.49568 |
|  |  |  |  |  |  |
| ***PROTEIN FATE (folding, modification, destination)*** |  |  |  |  |  |
|  |  |  |  |  |  |
| **Protein modification** |  |  |  |  |  |
|  |  |  |  |  |  |
| PAAG_01189.2 | Sulphydryl oxidase Sox | 6.26e-91 | Oxidoreductase activity | - | 0.710391 |
| PAAG_04420.2 | Tyrosine-protein phosphatase CDC14 | 1.04e-84 | Modification by phosphorylation, dephosphorylation, autophosphorylation | 3.1.3.48 | 1.02155 |
| PAAG_06207.2 | Maintenance of ploidy protein mob1 | 1.13e-38 | Modification by phosphorylation, dephosphorylation, autophosphorylation | - | 0.751513 |
| PAAG_02613.2 | Geranylgeranyl transferase type-2 subunit α | 1.29e-20 | Modification with fatty acids (e.g. myristylation, palmitylation, farnesylation) | 2.5.1.60 | 0.587621 |
| PAAG_04977.2 | Ubiquitin-conjugating enzyme | 1.50e-15 | Modification by ubiquitination, deubiquitination | 6.3.2.19 | 0.58847 |
| PAAG_07745.2 | Ubiquitin/metalloprotease fusion protein | 6.61e-20 | Peptidase activity | - | 0.754075 |
| PAAG_05067.2 | SprT family metallopeptidase | 1.86e-22 | hydrolysis of peptide | - | 0.634038 |
|  |  |  |  |  |  |
| **Protein/peptide degradation** |  |  |  |  |  |
|  |  |  |  |  |  |
| PAAG_01475.2 | Ubiquitin fusion degradation protein | 1.47e-216 | Proteasomal degradation (ubiquitin/proteasomal pathway) | 6.3.2.19 | 1.07806 |
|  |  |  |  |  |  |
| ***CLASSIFICATION NOT YET CLEAR-CUT*** |  |  |  |  |  |
|  |  |  |  |  |  |
| PAAG_05026.2 | Integral membrane protein | 1.27e-33 | - | - | 0.735744 |
| PAAG_06837.2 | Molybdopterin synthase small subunit CnxG | 1.76e-14 | - | - | 0.594081 |
| PAAG_06856.2 | GTP-binding protein EsdC | 1.94e-70 | - | - | 1.29802 |
| PAAG_06765.2 | Bax Inhibitor family protein | 0 | - | - | 1.07599 |
| PAAG_06153.2 | Ser/Thr protein phosphatase family protein | 7.29e-105 | - | - | 0.981347 |
| PAAG_00436.2 | Spo12 family protein | 1.42e-28 | - | - | 1.10696 |
| PAAG_06341.2 | Thioesterase family protein | 4.56e-09 | - | - | 1.12103 |
| PAAG_02974.2 | Glutaredoxin domain-containing protein | 1.16e-25 | - | - | 0.674465 |
| PAAG_01423.2 | HHE domain-containing protein | 4.20e-70 | - | - | 0.770069 |
| PAAG_01360.2 | FAD binding domain-containing protein | 3.01e-08 | - | - | 1.17989 |
|  |  |  |  |  |  |
| ***UNCLASSIFIED PROTEINS*** |  |  |  |  |  |
|  |  |  |  |  |  |
| PAAG_07459.2 | Conserved hypothetical protein | 4.67e-17 | - | - | 0.746592 |
| PAAG_08265.2 | Conserved hypothetical protein | 1.20e-50 | - | - | 0.970154 |
| PAAG_00658.2 | Conserved hypothetical protein | 8.25e-31 | - | - | 0.858255 |
| PAAG_01356.2 | Conserved hypothetical protein | 3.96e-12 | - | - | 0.68545 |
| PAAG_08618.2 | Conserved hypothetical protein | 1.27e-88 | - | - | 0.89793 |
| PAAG_05356.2 | Conserved hypothetical protein | 0 | - | - | 2.22597 |
| PAAG_05856.2 | Conserved hypothetical protein | 0 | - | - | 1.14463 |
| PAAG_03183.2 | Conserved hypothetical protein | 5.59e-285 | - | - | 0.749374 |
| PAAG_02616.2 | Conserved hypothetical protein | 7.14e-229 | - | - | 0.950659 |
| PAAG_04059.2 | Conserved hypothetical protein | 6.87e-183 | - | - | 0.728531 |
| PAAG_02096.2 | Conserved hypothetical protein | 2.25e-140 | - | - | 0.846682 |
| PAAG_06851.2 | Conserved hypothetical protein | 8.60e-125 | - | - | 0.885911 |
| PAAG_01354.2 | Conserved hypothetical protein | 6.00e-113 | - | - | 0.90444 |
| PAAG_03236.2 | Conserved hypothetical protein | 2.02e-93 | - | - | 0.76157 |
| PAAG_03635.2 | Conserved hypothetical protein | 5.99e-83 | - | - | 0.815244 |
| PAAG_02903.2 | Conserved hypothetical protein | 3.98e-78 | - | - | 0.673438 |
| PAAG_06231.2 | Conserved hypothetical protein | 2.99e-69 | - | - | 0.860229 |
| PAAG_04131.2 | Conserved hypothetical protein | 6.86e-65 | - | - | 0.903889 |
| PAAG_04869.2 | Conserved hypothetical protein | 3.37e-57 | - | - | 0.761035 |
| PAAG_05607.2 | Conserved hypothetical protein | 9.33e-54 | - | - | 0.743156 |
| PAAG_09115.2 | Conserved hypothetical protein | 5.28e-51 | - | - | 0.836439 |
| PAAG_03980.2 | Conserved hypothetical protein | 1.12e-50 | - | - | 0.624689 |
| PAAG_05650.2 | Conserved hypothetical protein | 8.57e-50 | - | - | 0.682022 |
| PAAG_02124.2 | Conserved hypothetical protein | 7.38e-41 | - | - | 0.869325 |
| PAAG_08079.2 | Conserved hypothetical protein | 1.88e-40 | - | - | 1.44223 |
| PAAG_06964.2 | Conserved hypothetical protein | 3.22e-32 | - | - | 0.833938 |
| PAAG_05657.2 | Conserved hypothetical protein | 2.10e-31 | - | - | 0.63342 |
| PAAG_03069.2 | Conserved hypothetical protein | 2.01e-29 | - | - | 0.630719 |
| PAAG_07325.2 | Conserved hypothetical protein | 5.30e-29 | - | - | 0.621291 |
| PAAG_01792.2 | Conserved hypothetical protein | 6.61e-28 | - | - | 0.819304 |
| PAAG_04802.2 | Conserved hypothetical protein | 7.38e-13 | - | - | 0.665474 |
| PAAG_04394.2 | Conserved hypothetical protein | 1.08e-11 | - | - | 0.706413 |
| PAAG_00112.2 | Conserved hypothetical protein | 3.54e-11 | - | - | 0.653287 |
| PAAG_08996.2 | Conserved hypothetical protein | 7.40e-11 | - | - | 0.658521 |
| PAAG_04658.2 | Conserved hypothetical protein | 0 | - | - | 0.605861 |
| PAAG_07361.2 | Conserved hypothetical protein | 0 | - | - | 1.08082 |
| PAAG_07623.2 | Hypothetical protein | 2.00e-05 | - | - | 0.901895 |
| PAAG_06531.2 | Hypothetical protein | 5.07e-07 | - | - | 1.56269 |
| PAAG_05606.2 | Hypothetical protein | 5.08e-12 | - | - | 0.867397 |
| PAAG_02986.2 | Hypothetical protein | 1.13e-12 | - | - | 0.847022 |
| PAAG_08626.2 | Hypothetical protein | 9.90e-17 | - | - | 0.602944 |
| PAAG_03021.2 | Hypothetical protein | 1.56e-22 | - | - | 0.798295 |
| PAAG_03508.2 | Hypothetical protein | 2.77e-162 | - | - | 1.07374 |
| PAAG_02699.2 | Hypothetical protein | 1.54e-169 | - | - | 0.636171 |
| PAAG_07199.2 | Hypothetical protein | 0 | - | - | 0.719559 |
| PAAG_06908.2 | Hypothetical protein | 8.05e-37 | - | - | 0.772538 |
| PAAG_08177.2 | Hypothetical protein | 2.26e-34 | - | - | 0.682141 |
| PAAG_01358.2 | Hypothetical protein | 2.26e-29 | - | - | 1.36637 |
| PAAG_01793.2 | Hypothetical protein | 3.30e-26 | - | - | 0.870758 |
| PAAG_04638.2 | Hypothetical protein | 1.09e-18 | - | - | 0.774577 |
| PAAG_02097.2 | Hypothetical protein | 1.99e-18 | - | - | 0.787852 |
| PAAG_08995.2 | Hypothetical protein | 8.90e-18 | - | - | 0.789171 |
| PAAG_08077.2 | Hypothetical protein | 4.39e-16 | - | - | 0.990817 |
| PAAG_02731.2 | Hypothetical protein | 5.54e-14 | - | - | 0.78104 |
| PAAG_03248.2 | Hypothetical protein | 1.36e-13 | - | - | 0.819269 |
| PAAG_03213.2 | Hypothetical protein | 7.86e-10 | - | - | 0.676757 |
| PAAG_04421.2 | Hypothetical protein | 4.39e-09 | - | - | 1.09179 |
| PAAG_03884.2 | Hypothetical protein | 3.18e-07 | - | - | 0.693073 |
| PAAG_08132.2 | Hypothetical protein | 8.21e-06 | - | - | 0.804168 |
| PAAG_04870.2 | Hypothetical protein | 3.37e-05 | - | - | 0.712381 |
| PAAG_02948.2 | Hypothetical protein | 5.48e-05 | - | - | 1.13565 |
| PAAG_07662.2 | Hypothetical protein | 0 | - | - | 0.664684 |
| PAAG_07334.2 | Hypothetical protein | 0 | - | - | 0.921869 |
| PAAG_08078.2 | Hypothetical protein | 0 | - | - | 0.916223 |
| PAAG_06832.2 | Hypothetical protein | 1.67e-05 | - | - | 0.647455 |
| PAAG_05678.2 | Hypothetical protein | 1.03e-133 | - | - | 1.35917 |
