# Supplementary material for: Effects of Argentilactone on the Transcriptional Profile, Cell Wall and Oxidative Stress of Paracoccidioides spp
Source: PLoS Negl Trop Dis. 2016 Jan 6;10(1):e0004309. doi: 10.1371/journal.pntd.0004309 (PMC4703379; doi:10.1371/journal.pntd.0004309)
Supplement: S3 Table — (DOCX) [file pntd.0004309.s005.docx]

**Supplementary Table 3:** Functional classification of down-regulated genes from *Paracoccidioides* yeast cellsin the presence argentilactone.

| **Accession number/ Functional classification** | **Gene product** | ***P-*value** | **Annotated function** | **EC number** | **Fold Change(Log_2_)** |
| --- | --- | --- | --- | --- | --- |
|  |  |  |  |  |  |
| ***METABOLISM*** |  |  |  |  |  |
|  |  |  |  |  |  |
| **Amino acid metabolism** |  |  |  |  |  |
|  |  |  |  |  |  |
| PAAG_04619.2 | Cystathionine gamma-synthase | 1.09e-12 | Cysteine and methionine metabolism | 2.5.1.48 | -0.813123 |
| PAAG_04464.2 | Glutamine-dependent NAD(+) synthetase | 2.43e-110 | Metabolism of glutamate | 6.3.5.1 | -0.994432 |
| PAAG_06008.2 | Cysteine synthase K/M/Cysteine synthase B | 6.18e-22 | Biosynthesis of cysteine | 2.5.1.47 | -0.5893 |
| PAAG_00966.2 | L-threonine 3-dehydrogenase | 1.57e-49 | Glycine, serine and threonine metabolism | 1.1.1.103 | -0.744757 |
| PAAG_05227.2 | Aryl-alcohol dehydrogenase | 1.90e-14 | Tyrosine metabolism | 1.1.1.90 | -0.730747 |
| PAAG_08910.2 | D-3-phosphoglycerate dehydrogenase | 1.85e-34 | Amino acid metabolism - biosynthesis of serine | 1.1.1.95 | -0.642961 |
| PAAG_07689.2 | NADP-specific glutamate dehydrogenase | 1.53e-71 | Degradation of glutamate - degradation of glutamine | 1.4.1.4 | -1.03874 |
| PAAG_02548.2 | Hydroxyacylglutathione hydrolase | 1.16e-23 | Metabolism of thioredoxin, glutaredoxin, glutathione | 3.1.2.6 | -0.608974 |
| PAAG_05932.2 | Phosphoadenosine phosphosulfate reductase | 5.53e-20 | Amino acid metabolism - nitrogen, sulfur and selenium metabolism | 1.8.4.8 | -1.07943 |
| PAAG_08641.2 | Galactonate dehydratase | 9.15e-19 | Metabolism of the cysteine - aromatic group | 4.2.1.6 | -0.748568 |
| PAAG_05754.2 | Averantin oxidoreductase | 1.65e-13 | Degradation of phenylalanine | 1.14.-.- | -0.855738 |
| [PAAG_11653.2](http://www.broadinstitute.org/annotation/genome/paracoccidioides_brasiliensis/FeatureRedirect.html?sp=S7000010310412637) | Hypothetical protein (Pyridoxal-5'-phosphate-dependent enzyme) | 0 | Cysteine and methionine metabolism | 2.5.1.47 | -0.729868 |
| PAAG_01563.2 | Aromatic-L-amino-acid decarboxylase | 0 | Degradation of phenylalanine | 4.1.1.28 | -1.75175 |
| PAAG_03153.2 | Ornithine decarboxylase | 1.96e-74 | Metabolism of secondary products derived from L-lysine, L-arginine and L-histidine | 4.1.1.17 | -0.745919 |
| PAAG_05743.2 | Aromatic amino acid aminotransferase | 4.10e-54 | Metabolism of the cysteine - aromatic group | 2.6.1.57 | -0.841952 |
| PAAG_08820.2 | Hypothetical protein (Tyrosinase) | 5.27e-93 | Metabolism of tyrosine | 1.14.18.1 | -1.72144 |
| PAAG_05853.2 | Indoleamine 2,3-dioxygenase | 7.24e-300 | Metabolism of tryptophan | 1.13.11.52 | -1.08579 |
|  |  |  |  |  |  |
| **Metabolism of vitamins** |  |  |  |  |  |
|  |  |  |  |  |  |
| PAAG_05612.2 | Pyridoxine Kinase | 5.79e-33 | Metabolism of cofactors and vitamins - Vitamin B6 metabolism | 2.7.1.35 | -0.881851 |
| PAAG_06102.2 | Riboflavin aldehyde-forming enzyme | 1.25e-19 | Catabolism of vitamins, cofactors, and prosthetic groups | - | -0.718607 |
| PAAG_08792.2 | Carotenoid cleavage dioxygenase | 2.58e-13 | Metabolism of vitamins, cofactors, and prosthetic groups | - | -0.989844 |
| PAAG_06924.2 | Glutamate-1-semialdehyde 2,1-aminomutase | 0 | Biosynthesis of vitamins, cofactors, and prosthetic groups | 5.4.3.8 | -0.994362 |
| PAAG_06925.2 | Hypothetical protein (Glutamate-1-semialdehyde 2,1-aminomutase) | 5.48e-06 | [Biosynthesis of vitamins, cofactors, and prosthetic groups](http://mips.gsf.de/cgi-bin/proj/funcatDB/search_advanced.pl?action=2&wert=01.07.01) | 5.4.3.8 | -0.823317 |
|  |  |  |  |  |  |
| **Lipid metabolism** |  |  |  |  |  |
|  |  |  |  |  |  |
| PAAG_08731.2 | Acetyl-CoA carboxylase | 0 | Lipid, fatty acid and isoprenoid metabolism | 6.4.1.2 | -0.853228 |
| PAAG_06005.2 | Phospholipase C 2 | 1.331e-11 | Glycerophospholipid metabolism | 3.1.-.-. | -1.00298 |
| PAAG_03235.2 | Lysophospholipase | 4.86e-35 | Phospholipid metabolism - Glycerophospholipid metabolism | 3.1.1.5 | -0.61494 |
| PAAG_06877.2 | Hypothetical protein (Triacylglycerol lipase) | 8.83e-11 | Lipid, fatty acid and isoprenoid metabolism - Acylglycerol degradation | 3.1.1.3 | -0.612797 |
| PAAG_04679.2 | Lipase | 1.41e-129 | Lipid, fatty acid and isoprenoid metabolism | 3.1.1.-. | -2.02346 |
| PAAG_05133.2 | Hypothetical protein (Lipase/esterase) | 6.04e-183 | Lipid, fatty acid and isoprenoid metabolism | 3.1.1.-. | -1.15343 |
| PAAG_04006.2 | Phosphatidylserine decarboxylase proenzyme | 0 | Lipid, fatty acid and isoprenoidmetabolism - Glycerophospholipid metabolism | 4.1.1.65 | -1.51455 |
| PAAG_01524.2 | Fatty acid synthase subunit β dehydratase | 0 | Lipid, fatty acid and isoprenoid metabolism - biosynthesis of palmitoyl-CoA | 2.3.1.86 | -0.771856 |
| PAAG_05912.2 | Palmitoyltransferase erf2 | 4.39e-31 | Modification with fatty acids | 2.3.1 | -0.631619 |
| PAAG_05072.2 | Serine palmitoyltransferase | 1.51e-273 | Sphingolipid metabolism | 2.3.1.50 | -0.92803 |
| PAAG_03525.2 | Mannosyl phosphorylinositol ceramide synthase SUR1 | 1.35e-28 | Lipid, fatty acid and isoprenoid metabolism | - | -0.64937 |
| PAAG_02016.2 | Hydroxymethylglutaryl-CoA synthase | 5.73e-25 | Lipid, fatty acid and isoprenoid metabolism - Synthesis and degradation of ketone bodies | 2.3.3.10 | -0.602908 |
| PAAG_03792.2 | Hypothetical protein (Dihydrodipicolinate synthase) | 1.53e-67 | Lipid, fatty acid and isoprenoid metabolism | 4.1.3.3 | -0.672877 |
| PAAG_03986.2 | Hypothetical protein (Linoleate diol synthase) | 5.28e-35 | Metabolism of eicosanoids | 1.13.11.44 | -0.74226 |
| PAAG_11463.2 | Phosphomevalonate kinase | 4.39e-27 | Lipid, fatty acid and isoprenoid metabolism | 2.7.4.2 | -0.9328 |
| PAAG_05999.2 | Phosphatidylinositol-4-phosphate 5-kinase its3 | 1.06e-57 | Lipid, fatty acid and isoprenoid metabolism - Inositol phosphate metabolism | 2.7.1.68 | -0.617027 |
| PAAG_02972.2 | Hypothetical protein (Ethanolamine kinase | 2.67e-35 | Lipid, fatty acid and isoprenoid metabolism - Glycerophospholipid metabolism | 2.7.1.82 | -0.779985 |
| PAAG_03123.2 | 3-oxoacyl-(acyl-carrier-protein) reductase | 1.43e-05 | Fatty acid metabolism | 1.1.1.100 | -0.756202 |
| PAAG_05791.2 | 3-hydroxy-3-methylglutaryl-coenzyme A reductase | 1.77e-100 | Lipid, fatty acid and isoprenoid metabolism | 1.1.1.34 | -0.664405 |
| PAAG_05210.2 | Formyl-coenzyme A transferase | 0 | Fatty acid metabolism | 2.8.3.16 | -0.651866 |
| PAAG_05285.2 | Hypothetical protein (UDP-glucose, sterol transferase) | 7.41e-05 | Glycolipid metabolism | 2.4.1.173 | -0.656667 |
| PAAG_03722.2 | Acyl-CoA desaturase | 0 | Lipid, fatty acid and isoprenoid metabolism | 1.14.19.1 | -0.698966 |
| PAAG_04266.2 | α - methylacyl-CoA racemase | 3.35e-94 | Lipid, fatty acid and isoprenoid metabolism | 5.1.99.4 | -0.931573 |
| PAAG_06816.2 | Oxidoreductase, short chain dehydrogenase | 9.93e-05 | Metabolism of eicosanoids | 1.-.-.-. | -0.614847 |
| PAAG_01506.2 | C-4 methylsterol oxidase | 2.53e-174 | Etracyclic and pentacyclic triterpenes (cholesterin, steroids and hopanoids) metabolism | 1.14.13.72 | -0.996529 |
| PAAG_02804.2 | PAP2 domain-containing protein | 1.01e-40 | Phospholipid metabolism | - | -0.976333 |
| PAAG_06636.2 | FADbinding domain-containing protein | 1.50e-14 | Lipid, fatty acid and isoprenoid metabolism | - | -0.618699 |
|  |  |  |  |  |  |
| **Carbohydrate metabolismo** |  |  |  |  |  |
|  |  |  |  |  |  |
| PAAG_05150.2 | ATP-citrate synthase subunit 1 | 8.66e-297 | C-compound and carbohydrate metabolism | 2.3.3.8 | -0.623643 |
| PAAG_06764.2 | α-amylase | 3.90e-79 | Starch catabolism to α-D-glucose (standard path) | 3.2.1.1 | -1.29003 |
| PAAG_07616.2 | Neutral trehalase | 8.18e-26 | C-compound and carbohydrate metabolism | 3.2.1.28 | -0.733081 |
| PAAG_01554.2 | Acid trehalase | 3.17e-08 | C-compound and carbohydrate metabolism | 3.2.1.28 | -0.703254 |
| PAAG_01286.2 | Oxidoreductase ucpA | 9.82e-07 | Sugar, glucoside, polyol and carboxylate anabolism | 1.-.-.-. | -0.642744 |
| PAAG_11512.2 | Hypotetical protein α-N-arabinofuranosidase A | 1.63e-06 | Polysaccharide metabolism | 3.2.1.55 | -0.797036 |
| PAAG_06828.2 | Hypothetical protein (NAD dependent epimerase/dehydratase | 6.96e-05 | C-compound and carbohydrate metabolism | 3.13.1.1 | -0.642744 |
| PAAG_01405.2 | Inositol oxygenase | 0 | Sugar, glucoside, polyol and carboxylate metabolism | 1.13.99.1 | -3.73602 |
| PAAG_07150.2 | UDP-galactopyranose mutase | 0 | [C-compound and carbohydrate metabolism](http://mips.gsf.de/cgi-bin/proj/funcatDB/search_advanced.pl?action=2&wert=01.05) | 5.4.99.9 | -1.71062 |
| PAAG_03331.2 | Hypotetical protein (Phosphoglucomutase-2 | 7.32e-51 | Sugar, glucoside, polyol and carboxylate metabolism | 5.4.2.8 | -0.687449 |
| PAAG_06304.2 | Hypothetical protein (Sterigmatocystin 8-O-methyltransferase | 1.87e-10 | C-compound and carbohydrate metabolism | 2.1.1.110 | -0.86043 |
| PAAG_11872.2 | Hypotetical protein (Acetate regulatory DNA binding protein FacB | 7.98e-89 | Regulation of C-compound and carbohydrate metabolism | - | -0.829729 |
| PAAG_08983.2 | Hypothetical protein (PHO85 cyclin-7 | 2.97e-06 | Regulation of C-compound and carbohydrate metabolism | - | -0.689757 |

|  |  |  |  |  |  |
| --- | --- | --- | --- | --- | --- |
| **Nulceotide/Nucleoside/**  **Nucleobase metabolism** |  |  |  |  |  |
|  |  |  |  |  |  |
| PAAG_06107.2 | Pyrimidine precursor biosynthesis enzyme THI5 | 2.74e-13 | pyrimidine nucleotide/nucleoside/nucleobase metabolism | - | -0.96059 |
| PAAG_04324.2 | Hypothetical protein (Adenosine deaminase | 4.03e-27 | [purin nucleotide/nucleoside/nucleobase metabolism](http://mips.gsf.de/cgi-bin/proj/funcatDB/search_advanced.pl?action=2&wert=01.03.01) | 3.5.4.4 | -1.13512 |
| \| PAAG_07529.2 \| \| --- \| \|  \| | Orotidine 5'-phosphate decarboxylase | 2.62e-08 | Nucleotide/nucleoside/nucleobase metabolismo | 4.1.1.23 | -0.587887 |
|  |  |  |  |  |  |
| **Phosphate Metabolism** |  |  |  |  |  |
|  |  |  |  |  |  |
| PAAG_01768.2 | AMP deaminase | 1.17e-120 | Phosphate metabolismo | 3.5.4.6 | -0.680029 |
| PAAG_02496.2 | Hypothethcal protein (Alkaline phosphatase | 3.12e-19 | modification by phosphorylation, dephosphorylation, autophosphorylation | 3.1.3.1 | -0.709909 |
| PAAG_06450.2 | Hypothetical protein (Tyrosyl-DNA phosphodiesterase | 4.67e-26 | Phosphate metabolism | 3.1.4.1 | -0.723186 |
| PAAG_08226.2 | Homeobox domain-containing protein | 0 | Regulation of phosphate metabolismo | - | -0.849889 |
| PAAG_04693.2 | YjeF domain-containing protein | 1.28e-30 | Phosphate metabolismo | - | -0.623489 |
|  |  |  |  |  |  |
| ***BIOGENESIS OF CELLULAR COMPONENTS*** |  |  |  |  |  |
|  |  |  |  |  |  |
| **Fungal-type cell wall biogenesis** |  |  |  |  |  |
|  |  |  |  |  |  |
| PAAG_06535.2 | Endochitinase | 3.05e-134 | Polysaccharide metabolismo | 3.2.1.14 | -1.46509 |
| PAAG_05178.2 | Chitin Deacetylase | 3.36e-36 | Polysaccharide metabolismo | 3.5.1.41 | -0.894305 |
| PAAG_00249.2 | Endo-1,3(4)–β–glucanase | 0 | Polysaccharide metabolismo | 3.2.1.6 | -1.24382 |
| PAAG_06885.2 | UDP-N-acetylglucosaminepyrophosphorylase | 4.10e-149 | Biosynthesis of chitin from D-fructose-6-phosphate | 2.7.7.23 | -0.717009 |
| PAAG_01760.2 | UDP-N-acetylglucosamine transporter YEA4 | 9.89e-43 | Chitin anabolismo | - | -0.707715 |
| PAAG_03796.2 | Glucan 1,3-β-glucosidase | 2.72e-13 | Biogenesisof cellular components: cell wall/ polysaccharide metabolism | 3.2.1.58 | -0.634195 |
| PAAG_05770.2 | Glucan 1,3-β-glucosidase | 3.11e-44 | Biogenesisof cellular components: cell wall/ polysaccharide metabolism | 3.2.1.58 | -1.05214 |
| PAAG_04235.2 | Hydrophobin | 2.29e-05 | Cell wall organization | - | -0.896524 |
| PAAG_00091.2 | β-glucan synthesis-associated protein KRE6 | 1.26e-268 | Biogenesisof cellular components: cell wall/ polysaccharide metabolism | - | -1.13551 |
| PAAG_03391.2 | Chitin synthase B | 5.20e-256 | Cell wall: polysaccharide metabolism - Amino sugar and nucleotide sugar metabolism | 2.4.1.16 | -0.91402 |
| PAAG_04665.2 | TOS1 | 2.60e-234 | Fungal-type cell wall | - | -1.27665 |
| PAAG_08845.2 | Chitin synthase regulator 3 | 2.09e-61 | Chitin anabolismo | - | -0.771053 |
| PAAG_07975.2 | Chitin synthase export chaperone | 1.08e-49 | Aminosaccharide anabolismo | - | -1.02959 |
| PAAG_02542.2 | Cell wall glucanase | 8.74e-35 | Fungal-type cell wall organization | - | -1.10774 |

| PAAG_04461.2 | Acetylxylan esterase Axe2 | 2.06e-06 | Polysaccharide metabolism | 3.1.1.72 | -0.842242 |
| --- | --- | --- | --- | --- | --- |
| PAAG_00064.2 | D-xylose-proton symporter | 1.53e-33 | C-compound and carbohydrate metabolism - Starch and sucrose metabolism | - | -0.766152 |
| PAAG_01581.2 | Mannan endo-1,6-α-mannosidase DCW1 | 5.93e-299 | cell wall | 3.2.1.101 | -1.37924 |
| PAAG_05407.2 | Mannosyl-oligosaccharide 1,2-α-mannosidase | 1.11e-15 | Cell wall/polysaccharide metabolism | 3.2.1.113 | -1.00092 |
| PAAG_08326.2 | Polysaccharide synthase Cps1 | 5.38e-143 | Cell Wall/Polysaccharide metabolismo | - | -1.94856 |
| PAAG_04123.2 | ER membrane protein Wsc4 | 1.55e-32 | Cel Wall | - | -0.97677 |
| PAAG_08971.2 | Rho GTPase activator | 8.87e-105 | Cel Wall | - | -0.676759 |
|  |  |  |  |  |  |
| ***TRANSCRIPTION*** |  |  |  |  |  |
|  |  |  |  |  |  |
| **RNA synthesis** |  |  |  |  |  |
|  |  |  |  |  |  |
| PAAG_05064.2 | Transcription factor prr1 | 2.64e-49 | Regulator of transcription fator | - | -0.712178 |
| PAAG_05640.2 | Transcription initiation factor TFIID subunit 5 | 1.66e-35 | Transcription activation | - | -0.61378 |
| PAAG_00645.2 | SRF-type transcription factor RlmA | 3.71e-105 | Regulation of transcription | - | -0.934536 |
| PAAG_07224.2 | CP2 transcription factor | 8.63e-60 | Transcriptional control | - | -0.644069 |
| PAAG_06572.2 | Transcription factor Snf5p | 3.49e-18 | Transcriptional control | - | -0.598675 |
| PAAG_08499.2 | Pre-mRNA-splicing factor ATP-dependent RNA helicase P | 1.60e-38 | RNA synthesis | - | -0.659151 |
| PAAG_08242.2 | Homeobox transcription factor | 9.34e-15 | DNA binding | - | -0.658135 |
| PAAG_04481.2 | Specific RNA polymerase II transcription factor | 2.25e-124 | Transcriptional control | - | -0.657043 |
| PAAG_03347.2 | RNA polymerase II transcription elongation factor Rtf1p | 5.14e-17 | General transcription activities | - | -0.59171 |
| PAAG_01533.2 | RNA interference and silencing protein | 9.41e-08 | Transcriptional control | - | -1.6948 |
| PAAG_07510.2 | DNA directed RNA polymerase II 15 kDa subunit | 1.73e-10 | mRNA synthesis | - | -0.659726 |
| PAAG_00191.2 | Replication factor C subunit 1 | 1.27e-43 | Specific transcriptional repressor activity | - | -0.729797 |
| PAAG_05322.2 | RNA-binding protein | 1.52e-77 | Transcription activation | - | -0.812602 |
| PAAG_04622.2 | mRNA binding protein Pumilio 2 | 2.88e-67 | Transcription repression | - | -0.643029 |
| PAAG_04815.2 | Nucleic acid-binding protein | 7.48e-29 | Transcription activation | - | -0.708842 |
| PAAG_07993.2 | Minichromosome loss protein | 1.39e-47 | Transcriptional control | - | -0.655904 |
| PAAG_08854.2 | GATA-factor | 3.17e-15 | Transcription activation | - | -0.867118 |
| PAAG_04790.2 | Small nuclear ribonucleoprotein SmG | 1.01e-14 | mRNA synthesis | - | -0.845876 |
| PAAG_03855.2 | ATP-dependent RNA helicase DBP2 | 3.20e-06 | mRNA synthesis | 3.6.4.13 | -1.19509 |
| PAAG_00962.2 | Ada histone acetyltransferase complex component | 9.48e-30 | transcriptional control | - | -0.832257 |
| PAAG_04185.2 | RNA-directed RNA polymerase | 5.85e-13 | Control of mRNA stability | 2.7.7.49 | -0.587999 |
| PAAG_08016.2 | mRNA cleavage factor complex component Pcf11 | 3.25e-27 | Transcription termination | - | -0.789067 |
| PAAG_05598.2 | MYB family conidiophore development protein FIbD | 5.44e-29 | Transcription activation | - | -1.07639 |
| PAAG_05007.2 | WD repeat-containing protein | 6.85e-21 | Transcription activation | - | -0.63418 |
| PAAG_07748.2 | WD40 repeat protein | 1.57e-19 | Transcription activation | - | -0.735046 |
| PAAG_03953.2 | WD repeat-containing protein | 1.13e-39 | Transcription activation | 1.11.1.5 | -0.627057 |
| PAAG_04865.2 | WD domain-containing protein | 4.96e-86 | Transcription activation | - | -0.810719 |
| PAAG_03287.2 | C2H2 transcription factor | 5.06e-270 | Transcriptional control | - | -0.983494 |
| PAAG_01393.2 | C2H2 transcription factor | 3.15e-133 | Transcription repression | - | -0.686891 |
| PAAG_07929.2 | C2H2 transcription factor | 1.77e-73 | Transcriptional control | - | -0.660052 |
| PAAG_02847.2 | C2H2 transcription factor | 1.46e-11 | Transcription repression | - | -1.44784 |
| PAAG_06455.2 | C2H2 transcription factor RfeC | 1.08e-110 | Transcriptional control | - | -0.794824 |
| PAAG_06637.2 | C2H2 finger domain-containing protein | 2.00e-71 | Transcriptional control | - | -0.613298 |
| PAAG_05759.2 | C2H2 finger domain-containing protein | 1.96e-68 | Transcriptional control | - | -0.757745 |
| PAAG_04497.2 | C2H2 finger domain-containing protein | 9.41e-21 | Transcriptional control | - | -0.781564 |
| PAAG_05724.2 | C2H2 finger domain-containing protein FlbC | 7.33e-07 | Transcriptional control | - | -0.675821 |
| PAAG_07831.2 | C2H2 type zinc finger domain-containing protein | 3.71e-65 | Transcriptional control | - | -0.649692 |
| PAAG_05609.2 | C2H2 type zinc finger domain-containing protein | 4.51e-58 | Transcriptional control | - | -1.00138 |
| PAAG_05105.2 | Fungal specific transcription factor domain-containing protein | 4.89e-182 | Transcription activation | - | -0.80011 |
| PAAG_08069.2 | Pumilio domain-containing protein | 6.52e-48 | Transcriptional control | - | -0.675983 |
| PAAG_02239.2 | Pumilio domain-containing protein | 2.01e-299 | Control of mRNA stability | - | -0.968558 |
| PAAG_03777.2 | LIM domain-containing protein | 2.68e-17 | Regulator of transcription factor | - | -0.759515 |
| PAAG_05362.2 | DUF455 domain-containing protein | 3.48e-101 | Control of mRNA stability | - | -0.79744 |
| PAAG_04914.2 | Zinc finger protein gcs 1 | 2.99e-32 | Transcriptional control | - | -0.892536 |
| PAAG_00893.2 | C6 transcription factor Ctf1B | 3.91e-106 | Transcriptional control | - | -0.920267 |
| PAAG_09082.2 | C6 transcription factor | 5.05e-68 | Regulation of transcription | - | -0.836891 |
| PAAG_05375.2 | C6 zinc finger domain-containing protein | 3.83e-71 | Transcriptional control | - | -0.761445 |
| PAAG_02331.2 | C6 zinc finger domain-containing protein | 1.69e-185 | transcription activation | - | -0.87487 |
| PAAG_01011.2 | JmjC domain-containing histone demethylation protein | 3.21e-33 | Transcriptional control | - | -0.604526 |
|  |  |  |  |  |  |
| **RNA processing** |  |  |  |  |  |
|  |  |  |  |  |  |
| PAAG_07966.2 | Pre-mRNA-splicing factor cwc15 | 1.37e-38 | Splicing | - | -0.673289 |
| PAAG_02507.2 | Pre-mRNA-splicing factor CWC23 | 1.26e-10 | Splicing | - | -0.671445 |
| PAAG_01344.2 | Antiviral helicase SKI2 | 1.17e-38 | rRNAprocessing | 3.6.4.- | -0.620734 |
| PAAG_05354.2 | Poly A polymerase | 4.39e-88 | mRNA processing (splicing, 5'-, 3'-end processing) | 2.7.7.19 | -0.678436 |
| PAAG_03637.2 | HLA class III protein Dom3z | 9.76e-08 | rRNA processing | - | -0.652892 |
|  |  |  |  |  |  |
| **RNA modification** |  |  |  |  |  |
|  |  |  |  |  |  |
| PAAG_03639.2 | tRNA isopentenyltransferase | 9.38e-08 | tRNA modification | 2.5.1.8 | -0.940627 |
|  |  |  |  |  |  |
| ***CELL RESCUE, DEFENSE AND VIRULENCE*** |  |  |  |  |  |
|  |  |  |  |  |  |
| **Stress Response** |  |  |  |  |  |
|  |  |  |  |  |  |
| PAAG_01106.2 | Short-chain dehydrogenase | 2.06e-09 | cellular sensing and response to external stimulus | 1.1.-.- | -0.589598 |
| PAAG_02357.2 | Short-chain dehydrogenase/reductase | 4.63e-18 | cellular sensing and response to external stimulus | 1.1.-.-. | -0.75433 |
| PAAG_05763.2 | Acid phosphatase | 3.52e-26 | heat shock response | 3.1.3.2 | -0.852418 |
| PAAG_07822.2 | Retinol dehydrogenase | 8.46e-17 | Cellular sensing and response to external stimulus | 1.1.1.105 | -0.657192 |
| PAAG_07528.2 | Glutathione S-transferase | 7.59e-53 | Stress response | 2.5.1.18 | -0.75381 |
| PAAG_04338.2 | Interferon-induced GTP-binding protein Mx | 9.87e-08 | Response to biotic stimulus | - | -0.635812 |
| PAAG_02169.2 | DnaJ domain-containing protein | 1.08e-53 | Stress response/heat shock response | - | -0.651989 |
| PAAG_03837.2 | Annexin ANXC4 | 8.50e-45 | Cellular export and secretion | - | -0.771338 |
|  |  |  |  |  |  |
| **Disease, virulence and defense** |  |  |  |  |  |
|  |  |  |  |  |  |
| PAAG_08253.2 | Acyltransferase family protein | 2.93e-46 | Virulence, disease factors | - | -0.731272 |
| PAAG_07947.2 | Integral membrane protein | 6.18e-20 | Disease, virulence and defense | - | -0.82623 |
| PAAG_05840.2 | Toxin-insensitive protein | 2.83e-20 | resistance proteins | - | -0.604199 |
|  |  |  |  |  |  |
| **Detoxification** |  |  |  |  |  |
|  |  |  |  |  |  |
| PAAG_02232.2 | Benzoate 4-monooxygenase cytochrome P450 | 1.43e-293 | Detoxification involving cytochrome P450 | 1.14.13.12 | -1.27157 |
| PAAG_07263.2 | Isotrichodermin C-15 hydroxylase | 4.42e-81 | Detoxification involving cytochrome P450 | 1.14.-.- | -0.969736 |
| PAAG_02166.2 | MFS multidrug transporter | 8.02e-90 | Drug/toxin transport: cellular export and secretion | - | -2.1123 |
| PAAG_06022.2 | MFS multidrug transporter | 3.20e-09 | Detoxification by export | - | -0.786668 |
| PAAG_07559.2 | MFS transporter | 2.33e-05 | Drug/toxin transport: cellular export and secretion | - | -0.678086 |
| PAAG_07544.2 | MFS transporter | 2.12e-14 | Drug/toxin transport | - | -0.939828 |
| PAAG_02191.2 | MFS transporter | 3.57e-51 | Response to drug/allantoin and allantoate transport | - | -1.78031 |
| PAAG_08227.2 | MFS transporter | 1.38e-07 | Response to drug/allantoin and allantoate transport | - | -0.85649 |
| PAAG_02484.2 | MFS transporter | 1.24e-05 | Transport facilities | - | -0.590465 |
| PAAG_06957.2 | MFS multidrug resistance transporter | 1.65e-10 | Drug/toxin transport | - | -1.01318 |
| PAAG_07694.2 | MFS drug efflux transporter | 9.39e-64 | Type I protein secretion system (ABC-type transport systems) | - | -1.26734 |
| PAAG_03217.2 | Multidrug transporter | 1.34e-59 | Drug/toxin transport | - | -1.28789 |
| PAAG_00061.2 | MFS sugar transporter | 0 | C-compound and carbohydrate transport | - | -0.760272 |
| PAAG_06432.2 | ABC multidrug transporter | 0 | Drug/toxin transport | - | -1.49317 |
| PAAG_03754.2 | Lipid A export ATP-binding/permease protein msbA | 2.06e-47 | Detoxification by export/ drug/toxin transport | 3.6.3.-. | -0.619198 |
| PAAG_07892.2 | Caffeine resistance protein | 1.47e-13 | Detoxification by export/ drug/toxin transport | - | -0.81756 |
| PAAG_04872.2 | Parasitic phase-specific protein PSP-1 | 3.02e-12 | Detoxification | - | -0.860509 |
| PAAG_01242.2 | Trichothecene efflux pump | 1.71e-06 | Drug/toxin transport: cellular export and secretion | - | -0.643458 |
|  |  |  |  |  |  |
| ***CELLULAR TRANSPORT, TRANSPORT FACILITIES AND TRANSPORT ROUTES*** |  |  |  |  |  |
|  |  |  |  |  |  |
| **Transported compounds (substrates)** |  |  |  |  |  |
|  |  |  |  |  |  |
| PAAG_03365.2 | Sulfate transporter | 1.54e-06 | Sulfate transport | 4.2.1.1 | -0.691997 |
| PAAG_05283.2 | Oligopeptide transporter | 1.07e-16 | Peptide transport | 3.6.3.23 | -0.669807 |
| PAAG_01453.2 | High-affinity glucose transporter | 1.47e-09 | C-compound and carbohydrate transport | - | -0.726577 |
| PAAG_01421.2 | High-affinity nicotinic acid transporter | 3.65e-05 | Vitamine/cofactor transport | - | -0.96424 |
| PAAG_08406.2 | High-affinity nicotinic acid transporter | 4.42e-08 | Vitamine/cofactor transport | - | -1.98621 |
| PAAG_06533.2 | Peptide transporter PTR2 | 3.02e-219 | Peptide transport | - | -1.21765 |
| PAAG_03863.2 | Acetyl-coenzyme A transporter 1 | 4.41e-64 | [Lipid/fatty acid transport](http://mips.gsf.de/cgi-bin/proj/funcatDB/search_advanced.pl?action=2&wert=20.01.13) | 2.3.1.-. | -0.954302 |
| PAAG_03300.2 | Sugar transporter | 8.03e-05 | C-compound and carbohydrate transport | - | -2.32324 |
| PAAG_08473.2 | Phthalate transporter | 3.35e-83 | Vitamine/cofactor transport | - | -1.20443 |
| PAAG_07726.2 | Nitrate transporter | 8.41e-08 | Nitrate transport | 3.6.3.26 | -0.608185 |
| PAAG_03034.2 | Plasma membrane calcium-transporting ATPase | 1.04e-111 | Cation transport (H+, Na+, K+, Ca2+, NH4+, etc.) | 3.6.3.8 | -0.771331 |
| PAAG_01267.2 | Potassium transporter hak-1 | 3.03e-61 | Cation transport (H+, Na+, K+, Ca2+ , NH4+, etc.) | - | -0.890336 |
| PAAG_03791.2 | Sodium transport ATPase | 2.22e-15 | cation transport (H+, Na+, K+, Ca2+ , NH4+, etc.) | 3.6.3.9 | -0.701755 |
| PAAG_00552.2 | Lysine-specific permease | 2.65e-96 | Amino acid/amino acid derivatives transport | - | -0.979191 |
| PAAG_07749.2 | Proline-specific permease | 2.85e-30 | Amino acid/amino acid derivatives transport | - | -1.02899 |
| PAAG_03721.2 | Purine permease | 6.12e-15 | Nucleotide/nucleoside/nucleobase transport | - | -1.12717 |
| PAAG_00426.2 | N amino acid transport system protein | 1.00e-21 | Amino acid/amino acid derivatives transport | - | -0.924039 |
| PAAG_07291.2 | Integral membrane protein | 5.05e-22 | C-compound and carbohydrate transport | - | -0.689166 |
| PAAG_06634.2 | Integral membrane protein | 2.17e-05 | C-compound and carbohydrate transport | - | -0.947734 |
| PAAG_00010.2 | Arabinose-proton symporter | 1.28e-17 | C-compound and carbohydrate transport | - | -0.667923 |
| PAAG_01176.2 | Solute carrier family 35 member C2 | 1.56e-30 | C-compound and carbohydrate transport | - | -0.931538 |
| PAAG_06677.2 | RTA1 domain-containing protein | 5.01e-10 | Lipid/fatty acid transport | - | -0.897317 |
| PAAG_03419 .2 | Zinc-regulated transporter 2 | 0 | Heavy metal ion transport (Cu+, Fe3+, etc.) | - | -1.4074 |
| PAAG_00255.2 | Vacuolar calcium ion transporter | 2.08e-94 | Vacuolar/lysosomal transport/homeostasis of metal ions (Na, K, Ca etc.) | - | -0.779501 |
| PAAG_00326.2 | Heavy metal ion transporter | 0 | Heavy metal ion transport (Cu+, Fe3+, etc.) | - | -1.05089 |
| PAAG_07885.2 | Zinc transporter 1 | 1.26e-104 | Heavy metal ion transport (Cu+, Fe3+, etc.) | - | -0.833476 |
| PAAG_09040.2 | Ferric-chelate reductase | 0 | Homeostasis of metal ions (Na, K, Ca etc.) | 1.16.1.7 | -2.11884 |
| PAAG_03081.2 | PHD finger domain-containing protein | 2.00e-43 | Metal ion binding | - | -0.654051 |
| PAAG_05543.2 | SET domain-containing protein 5 | 6.53e-05 | Metal ion binding | - | -0.612381 |
| PAAG_08789.2 | PHD finger domain-containing protein | 7.00e-105 | Metal ion binding | - | -0.590369 |
|  |  |  |  |  |  |
| **Transport routes** |  |  |  |  |  |
|  |  |  |  |  |  |
| PAAG_03834.2 | Vacuolar sorting-associated protein | 2.02e-15 | Vacuolar/lysosomal transport | - | -1.53 |
| PAAG_00236.2 | GTPase-activating protein gyp10 | 2.45e-29 | Vacuolar/lysosomal transport/GTPase activator (GAP) | - | -0.601789 |
| PAAG_00804.2 | Endoplasmic reticulum-Golgi intermediate compartment protein | 7.29e-17 | ER to Golgi transport | - | -0.59147 |
| PAAG_07712.2 | Surfeit locus protein | 1.72e-72 | ER to Golgi transport | - | -0.710549 |
| PAAG_03801.2 | Mitochondrial carrier protein | 9.63e-07 | Mitochondrial transport | - | -0.615652 |
| PAAG_09109.2 | Translin associated factor X | 6.94e-05 | Nuclear transport | - | -0.658052 |
| PAAG_03294.2 | Nuclear pore protein SEH1 | 1.02e-23 | Intracellular transport vesicles | - | -0.832092 |
|  |  |  |  |  |  |
| **Transport facilities** |  |  |  |  |  |
|  |  |  |  |  |  |
| PAAG_06228.2 | Translocation protein SEC62 | 1.12e-46 | Transportfacilities | - | -0.650994 |
| PAAG_07575.2 | Vacuolar amino acid transporter 2 | 3.12e-37 | Transport facilities | - | -0.59161 |
|  |  |  |  |  |  |
| ***ENERGY*** |  |  |  |  |  |
|  |  |  |  |  |  |
| **Fermentation** |  |  |  |  |  |
|  |  |  |  |  |  |
| PAAG_05367.2 | Alcohol dehydrogenase zinc-binding domain-containing protein | 8.91e-73 | [Alcohol fermentation](http://mips.gsf.de/cgi-bin/proj/funcatDB/search_advanced.pl?action=2&wert=02.16.01) | - | -0.838573 |
| PAAG_04541.2 | Alcohol dehydrogenase | 0 | [Alcohol fermentation](http://mips.gsf.de/cgi-bin/proj/funcatDB/search_advanced.pl?action=2&wert=02.16.01) | 1.1.1.1 | -0.961675 |
|  |  |  |  |  |  |
| **Respiration** |  |  |  |  |  |
|  |  |  |  |  |  |
| PAAG_01488.2 | Cytochrome c oxidase-assembly factor COX16 | 3.68e-69 | Anaerobic respiration | 1.9.3.1 | -0.838933 |
|  |  |  |  |  |  |
| **Energy conversion and regeneration** |  |  |  |  |  |
|  |  |  |  |  |  |
| PAAG_00031.2 | NADH-ubiquinone oxidoreductase 20 kDa subunit | 0 | Accessory proteins of electron transport and membrane-associated energy conservation | 1.6.5.3 | -3.49317 |
|  |  |  |  |  |  |
| **Electron transport and membrane-associated energy conservation** |  |  |  |  |  |
|  |  |  |  |  |  |
| PAAG_02515.2 | Cytochrome P450 55A1 | 1.25e-46 | Electron transport | 1.14.-.-. | -0.892483 |
| PAAG_05966.2 | Cytochrome b-245 heavychain subunit β | 2.00e-22 | Electron transport /oxidoreductase activity | - | -1.02649 |
| PAAG_06092.2 | Cytochrome c1 | 1.06e-20 | Electron transport | - | -1.19398 |
| PAAG_07727.2 | Nitrate reductase | 1.30e-22 | Electron transport /NAD/NADP binding | 1.7.1.2 | -0.818636 |
| PAAG_02960.2 | External NADH-ubiquinone oxidoreductase | 1.37e-160 | Electron transport and membrane-associated energy conservation | 1.6.5.3 | -0.863318 |
| PAAG_07044.2 | NADH-ubiquinone oxidoreductase complex 1/LYR family protein | 3.77e-05 | E[lectron transport and membrane-associated energy conservation](http://mips.gsf.de/cgi-bin/proj/funcatDB/search_advanced.pl?action=2&wert=02.11) | - | -0.604386 |
| PAAG_06984.2 | NADPH-dehydrogenase | 7.03e-07 | Electron transport and membrane-associated energy conservation | 1.6.99.1 | -0.705114 |
|  |  |  |  |  |  |
| ***CELLULAR COMMUNICATION/SIGNAL TRANSDUCTION MECHANISM*** |  |  |  |  |  |
|  |  |  |  |  |  |
| **Cellular signalling** |  |  |  |  |  |
|  |  |  |  |  |  |
| PAAG_01187.2 | Calpain-9 | 6.23e-20 | Cellular signalling | 3.4.22.- | -0.745817 |
| PAAG_02468.2 | Phosducin | 9.22e-206 | Regulator of G-protein signalling | - | -1.55699 |
| PAAG_07295.2 | A-pheromone receptor PreA | 6.23e-08 | G-protein coupled receptor signalling pathway | - | -1.18171 |
|  |  |  |  |  |  |
| ***CELL CYCLE AND DNA PROCESSING*** |  |  |  |  |  |
|  |  |  |  |  |  |
| **DNA processing** |  |  |  |  |  |
|  |  |  |  |  |  |
| PAAG_06722.2 | DNA replication licensing factor MCM7 | 5.03e-62 | DNA synthesis and replication | 3.6.4.12 | -0.604279 |
| PAAG_00227.2 | DNA replication licensing factor MCM3 | 1.53e-60 | DNA synthesis and replication | 3.6.4.12 | -0.770702 |
| PAAG_04269.2 | Chromatin assembly factor 1 subunit A | 2.40e-46 | DNA synthesis and replication | - | -0.744174 |
| PAAG_08112.2 | ARS binding protein ABP2 | 3.72e-26 | DNA synthesis and replication | - | -1.02627 |
| PAAG_01162.2 | GINS DNA replication complex subunit SID5 | 7.46e-39 | DNA synthesis and replication | - | -1.06931 |
| PAAG_04676.2 | Origin recognition complex subunit | 2.52e-27 | DNA synthesis and replication | - | -0.60557 |
| PAAG_04408.2 | UV DNA damage endonuclease | 3.11e-18 | DNA repair | 3.-.-.-. | -0.807277 |
| PAAG_00193.2 | Replication factor C subunit 3 | 2.20e-85 | DNA repair | - | -1.13528 |
| PAAG_01173.2 | DNA-(apurinic or apyrimidinic site) lyase | 8.20e-47 | DNA repair | 4.2.99.18 | -0.639934 |
| PAAG_03026.2 | DNA mismatch repair protein MSH5 | 3.83e-16 | DNA repair | - | -0.965149 |
| PAAG_07058.2 | DNA repair protein RAD50 | 3.41e-91 | DNA recombination and DNA repair | 3.6.-.- | -0.768399 |
| PAAG_04987.2 | DEAD/DEAH box DNA helicase Mer3 | 2.62e-06 | DNA recombination | - | -0.678383 |
| PAAG_08932.2 | DNA repair protein RAD18 | 4.63e-117 | DNA repair | - | -0.776455 |
| PAAG_01043.2 | DNA repair protein RAD8 | 4.07e-53 | DNA repair | - | -0.935501 |
| PAAG_06575.2 | Nucleus protein | 1.25e-22 | DNA conformation modification | - | -0.670906 |
| PAAG_05267.2 | SWR1-complex protein | 6.17e-102 | DNA conformation modification (e.g. chromatin) | - | -1.37867 |
| PAAG_03904.2 | DNA-directed RNA polymerase III complex subunit Rpc37 | 1.33e-21 | DNA-directed RNA polymerase activity | - | -0.615787 |
|  |  |  |  |  |  |
| **Cell cycle** |  |  |  |  |  |
|  |  |  |  |  |  |
| PAAG_00581.2 | G1/S-specific cyclin Pcl5 | 9.37e-133 | G1/S transition of mitotic cell cycle | - | -0.613923 |
| PAAG_02260.2 | RNA exonuclease | 7.90e-08 | Mitotic cell cycle and cell cycle control | 3.1.-.-. | -0.857932 |
| PAAG_07512.2 | Histone-lysine N-methyltransferase SET9 | 1.95e-34 | Organization of chromosome structure | 2.1.1.43 | -0.700145 |
| PAAG_02310.2 | DNA replication licensing factor MCM7 component | 3.06e-80 | Mitotic cell cycle and cell cycle control | 3.6.4.12 | -0.705736 |
| PAAG_08525.2 | Mitosis inhibitor protein kinase SWE1 | 1.19e-77 | Cell cycle checkpoints (checkpoints of morphogenesis, DNA-damage,-replication, mitotic phase and spindle) | 2.7.11.1 | -0.598113 |
| PAAG_03835.2 | Sep4b | 4.47e-48 | Mitotic cell cycle and cell cycle control | 3.1.4.1 | -0.69383 |
| PAAG_00317.2 | Septin 4 | 1.13e-137 | Cytokinesis (cell division) /septum formation and hydrolysis | - | -0.828039 |
| PAAG_00980.2 | cyclin | 3.74e-66 | Mitotic cell cycle and cell cycle control | - | -0.608041 |
| PAAG_07899.2 | GTPase-activating protein | 1.13e-106 | Cell cycle dependent actin filament reorganization | - | -0.876662 |
| PAAG_06853.2 | DNA-directed RNA polymerase II 138 kDa polypeptide | 1.55e-41 | Induction of apoptosis | 2.7.7.6 | -0.759062 |
| PAAG_06330.2 | Topoisomerase 1-associated factor 1 | 1.33e-37 | Cell cycle checkpoints (checkpoints of morphogenesis, DNA-damage, replication, mitotic phase and spindle) | 5.99.1.2 | -0.725679 |
| PAAG_02954.2 | Mucin | 1.24e-129 | Mitotic cell cycle and cell cycle control | - | -0.859363 |
| PAAG_02230.2 | Cell division control protein | 6.37e-53 | Cytokinesis (cell division) /septum formation and hydrolysis | 2.7.-.-. | -0.693303 |
| PAAG_01035.2 | Cell division control protein | 2.38e-70 | Mitotic cell cycle and cell cycle control | 2.7.-.-. | -0.712983 |
| PAAG_07675.2 | Cell division control protein | 8.39e-87 | Cytokinesis (cell division) /septum formation and hydrolysis | 2.7.-.-. | -0.805367 |
| PAAG_03113.2 | Mitotic control protein dis3 | 8.03e-191 | Mitotic cell cycle and cell cycle control | - | -1.08293 |
| PAAG_08460.2 | RNA-binding protein CIP2 | 1.20e-85 | Cell cycle arrest | - | -0.620488 |
| PAAG_01493.2 | Chromosome segregation in meiosis protein 3 | 1.85e-79 | Chromosome segregation/division | - | -0.940495 |
| PAAG_00153.2 | Chromosome segregation protein sudA | 2.10e-122 | Chromosome condensation | - | -0.660021 |
| PAAG_03128.2 | Target of rapamycin complex subunit LST8 | 1.66e-41 | Cytokinesis (cell division) /septum formation and hydrolysis | - | -0.732956 |
| PAAG_04521.2 | Smc5-6 complex SMC subunit SMC5 | 7.90e-137 | Organization of chromosome structure | - | -0.991815 |
| PAAG_01318.2 | Chromatin remodeling complex subunit | 7.06e-25 | Organization of chromosome structure | - | -0.685283 |
| PAAG_08921.2 | Meiotically up-regulated gene 185 protein | 2.34e-30 | Mitotic cell cycle and cell cycle control | - | -0.732716 |
| PAAG_04527.2 | Rad21/Rec8 N terminal domain-containing protein | 3.82e-09 | Chromosome segregation/division | - | -0.927571 |
|  |  |  |  |  |  |
| ***CELL FATE*** |  |  |  |  |  |
|  |  |  |  |  |  |
| **Cell growth/Morphogenesis** |  |  |  |  |  |
|  |  |  |  |  |  |
| PAAG_06503.2 | NADPH oxidase regulator NoxR | 3.32e-76 | [cell growth / morphogenesis](http://mips.gsf.de/cgi-bin/proj/funcatDB/search_advanced.pl?action=2&wert=40.01) | - | -0.797156 |
| PAAG_07740.2 | β-chimaerin | 1.11e-275 | Cell growth / morphogenesis | - | -1.2037 |
| PAAG_05789.2 | Cell polarity protein | 2.45e-59 | Directional cell growth (morphogenesis) | - | -0.736457 |
| PAAG_00990.2 | DUF907 domain-containing protein | 2.26e-16 | Cell growth / morphogenesis | - | -0.658846 |
| PAAG_00473.2 | DUF89 domain-containing protein | 1.71e-72 | Cell growth / morphogenesis | - | -0.994452 |
|  |  |  |  |  |  |
| **Cell death** |  |  |  |  |  |
|  |  |  |  |  |  |
| PAAG_01989.2 | Autophagy-related protein 11 | 8.41e-265 | Apoptosis (type I programmed cell death) | - | -0.839391 |
|  |  |  |  |  |  |
| ***CELL TYPE DIFFERENTIATION*** |  |  |  |  |  |
|  |  |  |  |  |  |
| **Fungal/microorganismic cell type differentiation** |  |  |  |  |  |
|  |  |  |  |  |  |
| PAAG_02244.2 | Differentiation regulator | 4.22e-51 | Fungal/microorganismic development | - | -1.05691 |
| PAAG_03579.2 | cAMP-independent regulatory protein PAC2 | 1.77e-138 | Fungal and other eukaryotic cell type differentiation | - | -0.790023 |
| PAAG_03070.2 | Sexual development activator VeA | 2.87e-55 | Fungal and other eukaryotic cell type differentiation | - | -1.24954 |
| PAAG_03921.2 | HEP-C domain-containing protein PH085 | 5.46e-46 | Fungal and other eukaryotic cell type differentiation | - | -0.638075 |
| PAAG_00641.2 | Morphogenesis protein | 5.10e-143 | Budding, cell polarity and filament formation | - | -0.893375 |
| PAAG_05714.2 | Cellular morphogenesis protein | 1.95e-132 | Budding, cell polarity and filament formation | - | -0.762906 |
| PAAG_08522.2 | Nuclear migration protein | 2.53e-129 | Budding, cell polarity and filament formation | - | -0.875322 |
| PAAG_00387.2 | Pyoverdine biosynthesis protein PvcA | 1.49e-13 | Development of asco- basidio- or zygospore | - | -0.695915 |
| PAAG_01034.2 | DUF221 domain-containing protein | 2.72e-116 | Development of asco- basidio- or zygospore | - | -1.03423 |
| PAAG_06086.2 | DUF221 domain-containing protein | 3.53e-71 | Development of asco- basidio- or zygospore | - | -1.41607 |
|  |  |  |  |  |  |
| ***PROTEIN SYNTHESIS*** |  |  |  |  |  |
|  |  |  |  |  |  |
| **Ribosome biogenesis** |  |  |  |  |  |
|  |  |  |  |  |  |
| PAAG_08621.2 | Ribosome biogenesis protein Kri1 | 2.70e-91 | Ribosome biogenesis | - | -0.729116 |
|  |  |  |  |  |  |
| ***INTERACTION WITH THE ENVIRONMENT*** |  |  |  |  |  |
|  |  |  |  |  |  |
| **Cell Motility** |  |  |  |  |  |
|  |  |  |  |  |  |
| PAAG_00058.2 | Ankyrin repeat domain-containing protein | 4.46e-14 | Cell migration | - | -0.684827 |
| PAAG_08035.2 | Ankyrin repeat and protein kinase domain-containing protein | 3.16e-52 | Cell migration | - | -0.833122 |
| PAAG_00785.2 | Leucine Rich Repeat domain-containing protein | 4.69e-44 | Cell migration | - | -0.787235 |
|  |  |  |  |  |  |
| ***PROTEIN FATE (folding, modification, destination)*** |  |  |  |  |  |
|  |  |  |  |  |  |
| **Protein folding and stabilization** |  |  |  |  |  |
|  |  |  |  |  |  |
| PAAG_01838.2 | DnaJ domain-containing protein | 1.28e-153 | Protein folding and stabilization | - | -0.816545 |
|  |  |  |  |  |  |
| **Protein targeting, sorting and translocation** |  |  |  |  |  |
|  |  |  |  |  |  |
| PAAG_04722.2 | Cytoplasmic dynein 1 intermediate chain 2 | 8.48e-23 | Protein targeting, sorting and translocation | - | -0.659643 |
| PAAG_02523.2 | GTP-binding protein | 6.11e-51 | Translation | - | -0.678724 |
|  |  |  |  |  |  |
| **Protein modification** |  |  |  |  |  |
|  |  |  |  |  |  |
| PAAG_00826.2 | Calcium/calmodulin-dependent protein kinase type IV | 3.38e-50 | Regulation of protein activity | 2.7.11.11 | -0.732045 |
| PAAG_06271.2 | Cyclin-dependent protein kinase PH085 | 8.06e-46 | Enzymatic activity regulation / enzyme regulator | - | -0.806326 |
| PAAG_00767.2 | Histidine Kinase M7 | 3.76e-37 | Modification by phosphorylation, dephosphorylation | - | -0.801902 |
| PAAG_06070.2 | Serine/threonine-protein kinase ppk4 | 1.08e-148 | Kinase activity | 2.7.1.-. | -0.595561 |
| PAAG_06510.2 | Serine/threonine protein kinase | 1.56e-261 | Modification by phosphorylation, dephosphorylation, autophosphorylation | - | -1.07049 |
| PAAG_04047.2 | Serine/threonine protein kinase SGK2 | 0 | Modification by phosphorylation, dephosphorylation, autophosphorylation | - | -0.939232 |
| PAAG_02962.2 | Serine/threonine-protein kinase DCLK1 | 6.08e-95 | Modification by phosphorylation, dephosphorylation, autophosphorylation | 2.7.11.1 | -0.855201 |
| PAAG_01869.2 | Serine/threonine-protein kinase SAPK3 | 9.21e-31 | Modification by phosphorylation, dephosphorylation, autophosphorylation | 2.7.11.1 | -1.15445 |
| PAAG_04978.2 | Serine/threonine-protein kinase CHK1 | 3.19e-05 | Modification by phosphorylation, dephosphorylation, autophosphorylation | 2.7.11.1 | -0.687437 |
| PAAG_02376.2 | Serine/threonine-protein phosphatase PP-X isozyme | 2.17e-16 | Modification by phosphorylation, dephosphorylation, autophosphorylation | 3.1.3.16 | -0.799829 |
| PAAG_05740.2 | Serine/threonine-protein kinase ppk11 | 5.31e-26 | Modification by phosphorylation, dephosphorylation, autophosphorylation | - | -0.630064 |
| PAAG_00583.2 | Kinase domain-containing protein | 1.53e-05 | Modification by phosphorylation, dephosphorylation, autophosphorylation | - | -0.756202 |
| PAAG_01090.2 | Kinase domain-containing protein | 5.90e-10 | Modification by phosphorylation, dephosphorylation, autophosphorylation | - | -0.753759 |
| PAAG_01485.2 | Protein tyrosine phosphatase | 0 | Modification by phosphorylation, dephosphorylation, autophosphorylation | 3.1.3.48 | -0.637742 |
| PAAG_01674.2 | Ubiquitin carboxyl-terminal hydrolase | 9.99e-40 | Modification by ubiquitination, deubiquitination | 3.4.19.12 | -1.19453 |
| PAAG_02254.2 | Ubiquitin carboxyl-terminal hydrolase | 3.83e-32 | modification by ubiquitination, deubiquitination | 3.4.19.12 | -0.674842 |
| PAAG_05092.2 | Ubiquitin thiolesterase | 1.47e-177 | Modification by ubiquitination, deubiquitination | 3.1.2.15 | -0.929415 |
| PAAG_03454.2 | Ubiquitin-conjugating enzyme | 1.08e-22 | Modification by ubiquitination, deubiquitination | 6.3.2.19 | -1.06597 |
| PAAG_04901.2 | Ubiquitin-conjugating enzyme | 3.74e-82 | Modification by ubiquitination, deubiquitination | 6.3.2.19 | -0.70041 |
| PAAG_06425.2 | PHD finger and SET domain-containing protein | 1.99e-191 | Modification by acetylation, deacetylation | - | -0.720289 |
| PAAG_00542.2 | Guanine nucleotide-binding protein α-3 subunit | 3.20e-98 | Trimeric G-protein mediated signal transduction | 3.6.1.46 | -1.08185 |
| PAAG_00351.2 | Hydrolase | 2.31e-27 | Hydrolase activity | - | -0.800237 |
| PAAG_01058.2 | α/β hydrolase | 3.12e-06 | Transferase activity | - | -0.707673 |
| PAAG_04919.2 | Nucleoside-diphosphate-sugar epimerase | 1.77e-14 | Lyase activity | - | -0.601713 |
| PAAG_02916.2 | GNAT family N-acetyltransferase | 3.17e-30 | Transferase activity | 2.3.1.57 | -1.00774 |
| PAAG_07695.2 | *O*-methyltransferase | 2.97e-16 | Transferase activity | 2.1.1.110 | -1.13217 |
| PAAG_01406.2 | Methyltransferase type 11 | 2.36e-14 | Transferase activity | - | -1.44547 |
| PAAG_07934.2 | Methyltransferase | 1.22e-09 | Transferase activity | - | -0.695212 |
| PAAG_02662.2 | Phosphonoacetate hydrolase | 8.08e-10 | Hydrolase activity | 3.11.1.2 | -0.673572 |
| PAAG_06812.2 | Arylsulfatase | 3.19e-08 | Hydrolase activity | 3.1.6.1 | -0.80541 |
| PAAG_02708.2 | Rho guanyl nucleotide exchange factor | 2.63e-50 | Guanyl-nucleotide exchange factor (GEF) | - | -0.645613 |
| PAAG_00348.2 | Glutaredoxin domain-containing protein | 5.57e-10 | Detoxification by modification | - | -1.01801 |
| PAAG_07981.2 | TAM domain methyltransferase | 1.60e-13 | Transferase activity | - | -1.13839 |
| PAAG_08732.2 | HET-s/LopB domain-containing protein | 8.86e-130 | Kinase activity | - | -0.591786 |
| PAAG_08275.2 | Phosphotransferase enzyme family protein | 1.98e-06 | Transferase activity | - | -0.594882 |
| PAAG_06541.2 | SPFH domain / Band 7 family | 2.03e-40 | Protein binding | - | -0.59663 |
| PAAG_01300.2 | Coiled-coil domain-containing protein | 2.48e-22 | Hydrolase activity | - | -0.817232 |
| PAAG_04890.2 | CNH domain-containing protein | 9.09e-36 | Regulator of G-protein signaling | - | -0.664138 |
|  |  |  |  |  |  |
| **Protein/peptide degradation** |  |  |  |  |  |
|  |  |  |  |  |  |
| PAAG_02915.2 | Dipeptidase | 8.22e-09 | Protein/peptide degradation | 3.4.13 | -0.88959 |
| PAAG_00888.2 | 26S proteasome non-ATPase regulatory subunit 10 | 2.34e-09 | [Proteasomal degradation (ubiquitin/proteasomal pathway)](http://mips.gsf.de/cgi-bin/proj/funcatDB/search_advanced.pl?action=2&wert=14.13.01.01) | - | -0.664396 |
|  |  |  |  |  |  |
| ***CLASSIFICATION NOT YET CLEAR-CUT*** |  |  |  |  |  |
|  |  |  |  |  |  |
| PAAG_04490.2 | Expression library immunization antigen 1 | 2.22e-23 | - | - | -0.821983 |
| PAAG_02968.2 | PQ loop repeat protein | 5.99e-07 | - | - | -0.594009 |
| PAAG_03568.2 | Mitochondrial outer membrane protein MMM1 | 7.08e-53 | - | - | -0.890896 |
| PAAG_03202.2 | Mannose-P-dolichol utilization defect 1 protein | 2.07e-18 | - | - | -0.734308 |
| PAAG_01244.2 | β - lactamase family protein | 1.99e-131 | - | - | -1.24237 |
| PAAG_05483.2 | Ser/Thr protein phosphatase family protein | 7.89e-43 | - | - | -1.15929 |
| PAAG_08332.2 | FAD dependent oxidoreductase superfamily | 3.46e-31 | - | - | -0.5948 |
| PAAG_05552.2 | DUF614 domain-containing protein | 1.45e-05 | - | - | -0.600083 |
| PAAG_00532.2 | Cupin domain-containing protein | 0 | - | - | -1.2587 |
| PAAG_08419.2 | Cupin domain-containing protein | 6.27e-55 | - | - | -0.699522 |
| PAAG_08388.2 | SAC3/GANP domain-containing protein | 1.32e-71 | - | - | -0.958381 |
| PAAG_06408.2 | Sulfatase domain-containing protein | 5.64e-05 | - | - | -0.663093 |
| PAAG_06448.2 | SAM and PH domain-containing protein | 9.66e-08 | - | - | -1.02368 |
| PAAG_05037.2 | HHE domain-containing protein | 4.63e-11 | - | - | -0.644721 |
| PAAG_00912.2 | Prp 4 CRoW domain-containing protein | 6.10e-11 | - | - | -1.31096 |
| PAAG_08511.2 | MOSC domain-containing protein | 4.97e-24 | - | - | -0.87858 |
| PAAG_00879.2 | WW domain-containing protein | 1.27e-19 | - | - | -0.607203 |
|  |  |  |  |  |  |
| ***UNCLASSIFIED PROTEINS*** |  |  |  |  |  |
|  |  |  |  |  |  |
| PAAG_08256.2 | Conserved hypothetical protein | 2.81e-52 | - | - | -0.758655 |
| PAAG_00913.2 | Conserved hypothetical protein | 2.23e-08 | - | - | -0.864579 |
| PAAG_03870.2 | Conserved hypothetical protein | 4.21e-06 | - | - | -1.08329 |
| PAAG_06938.2 | Conserved hypothetical protein | 0 | - | - | -0.592704 |
| PAAG_06712.2 | Conserved hypothetical protein | 0 | - | - | -0.690614 |
| PAAG_04105.2 | Conserved hypothetical protein | 1.12e-06 | - | - | -0.725486 |
| PAAG_03425.2 | Conserved hypothetical protein | 1.00e-91 | - | - | -0.744826 |
| PAAG_04929.2 | Conserved hypothetical protein | 1.93e-14 | - | - | -0.754609 |
| PAAG_04369.2 | Conserved hypothetical protein | 2.69e-12 | - | - | -0.650074 |
| PAAG_05796.2 | Conserved hypothetical protein | 6.81e-11 | - | - | -0.686476 |
| PAAG_05469.2 | Conserved hypothetical protein | 4.72e-18 | - | - | -0.656862 |
| PAAG_02529.2 | Conserved hypothetical protein | 4.44e-13 | - | - | -0.623586 |
| PAAG_06858.2 | Conserved hypothetical protein | 2.37e-25 | - | - | -0.627185 |
| PAAG_06315.2 | Conserved hypothetical protein | 5.93e-52 | - | - | -0.698258 |
| PAAG_02661.2 | Conserved hypothetical protein | 2.83e-13 | - | - | -0.631147 |
| PAAG_04177.2 | Conserved hypothetical protein | 5.84e-05 | - | - | -1.00774 |
| PAAG_06768.2 | Conserved hypothetical protein | 3.19e-16 | - | - | -0.704022 |
| PAAG_07451.2 | Conserved hypothetical protein | 7.39e-262 | - | - | -1.48218 |
| PAAG_07257.2 | Conserved hypothetical protein | 1.67e-221 | - | - | -1.87313 |
| PAAG_03912.2 | Conserved hypothetical protein | 1.61e-189 | - | - | -1.4707 |
| PAAG_07269.2 | Conserved hypothetical protein | 5.31e-183 | - | - | -0.980911 |
| PAAG_01925.2 | Conserved hypothetical protein | 4.35e-147 | - | - | -2.13081 |
| PAAG_01749.2 | Conserved hypothetical protein | 5.27e-139 | - | - | -0.678032 |
| PAAG_12282.2 | Conserved hypothetical protein | 5.90e-121 | - | - | -0.939798 |
| PAAG_03359.2 | Conserved hypothetical protein | 1.48e-116 | - | - | -1.45583 |
| PAAG_06454.2 | Conserved hypothetical protein | 1.81e-113 | - | - | -0.871033 |
| PAAG_04626.2 | Conserved hypothetical protein | 5.99e-100 | - | - | -0.877145 |
| PAAG_05277.2 | Conserved hypothetical protein | 6.68e-98 | - | - | -0.902162 |
| PAAG_02133.2 | Conserved hypothetical protein | 4.68e-93 | - | - | -0.741858 |
| PAAG_02031.2 | Conserved hypothetical protein | 0 | - | - | -1.02699 |
| PAAG_06301.2 | Conserved hypothetical protein | 0 | - | - | -0.683507 |
| PAAG_07152.2 | Conserved hypothetical protein | 0 | - | - | -1.47227 |
| PAAG_03559.2 | Conserved hypothetical protein | 1.20e-319 | - | - | -1.27167 |
| PAAG_01196.2 | Conserved hypothetical protein | 2.36e-177 | - | - | -1.60412 |
| PAAG_00032.2 | Conserved hypothetical protein | 3.75e-165 | - | - | -1.53611 |
| PAAG_00128.2 | Conserved hypothetical protein | 2.76e-137 | - | - | -0.953735 |
| PAAG_01813.2 | Conserved hypothetical protein | 0 | - | - | -1.3862 |
| PAAG_08743.2 | Conserved hypothetical protein | 1.67e-132 | - | - | -0.75302 |
| PAAG_03243.2 | Conserved hypothetical protein | 4.49e-110 | - | - | -0.647119 |
| PAAG_06227.2 | Conserved hypothetical protein | 8.16e-107 | - | - | -1.02725 |
| PAAG_12593.2 | Conserved hypothetical protein | 8.41e-105 | - | - | -0.920178 |
| PAAG_03831.2 | Conserved hypothetical protein | 3.99e-92 | - | - | -1.06283 |
| PAAG_05813.2 | Conserved hypothetical protein | 2.92e-84 | - | - | -1.52808 |
| PAAG_07060.2 | Conserved hypothetical protein | 4.53e-84 | - | - | -0.74393 |
| PAAG_01637.2 | Conserved hypothetical protein | 9.53e-82 | - | - | -0.778929 |
| PAAG_01501.2 | Conserved hypothetical protein | 1.41e-80 | - | - | -1.55071 |
| PAAG_07021.2 | Conserved hypothetical protein | 1.91e-77 | - | - | -0.60373 |
| PAAG_06266.2 | Conserved hypothetical protein | 2.91e-76 | - | - | -0.877819 |
| PAAG_01204.2 | Conserved hypothetical protein | 8.94e-74 | - | - | -0.792907 |
| PAAG_01312.2 | Conserved hypothetical protein | 8.46e-73 | - | - | -0.676559 |
| PAAG_02546.2 | Conserved hypothetical protein | 0 | - | - | -2.0412 |
| PAAG_04383.2 | Conserved hypothetical protein | 3.56e-67 | - | - | -1.18734 |
| PAAG_05387.2 | Conserved hypothetical protein | 3.55e-67 | - | - | -1.04737 |
| PAAG_03393.2 | Conserved hypothetical protein | 6.99e-67 | - | - | -1.66825 |
| PAAG_06649.2 | Conserved hypothetical protein | 1.01e-65 | - | - | -1.04281 |
| PAAG_02778.2 | Conserved hypothetical protein | 1.39e-65 | - | - | -1.22402 |
| PAAG_08553.2 | Conserved hypothetical protein | 7.49e-70 | - | - | -0.679699 |
| PAAG_02153.2 | Conserved hypothetical protein | 1.48e-63 | - | - | -0.797219 |
| PAAG_00312.2 | Conserved hypothetical protein | 8.18e-62 | - | - | -1.49049 |
| PAAG_06713.2 | Conserved hypothetical protein | 9.25e-62 | - | - | -0.901242 |
| PAAG_06154.2 | Conserved hypothetical protein | 2.25e-59 | - | - | -0.64532 |
| PAAG_06620.2 | Conserved hypothetical protein | 7.41e-59 | - | - | -0.909865 |
| PAAG_01228.2 | Conserved hypothetical protein | 9.37e-59 | - | - | -1.35655 |
| PAAG_02245.2 | Conserved hypothetical protein | 7.31e-58 | - | - | -0.703827 |
| PAAG_03453.2 | Conserved hypothetical protein | 2.79e-57 | - | - | -0.661234 |
| PAAG_02093.2 | Conserved hypothetical protein | 5.71e-56 | - | - | -1.15597 |
| PAAG_11249.2 | Conserved hypothetical protein | 1.68e-55 | - | - | -0.738044 |
| PAAG_01285.2 | Conserved hypothetical protein | 3.50e-54 | - | - | -0.812599 |
| PAAG_05554.2 | Conserved hypothetical protein | 2.11e-53 | - | - | -0.632905 |
| PAAG_07703.2 | Conserved hypothetical protein | 5.68e-53 | - | - | -0.730123 |
| PAAG_06260.2 | Conserved hypothetical protein | 6.04e-51 | - | - | -0.661761 |
| PAAG_00307.2 | Conserved hypothetical protein | 3.65e-50 | - | - | -1.0525 |
| PAAG_04959.2 | Conserved hypothetical protein | 4.03e-50 | - | - | -0.679084 |
| PAAG_03100.2 | Conserved hypothetical protein | 1.54e-49 | - | - | -1.04508 |
| PAAG_07711.2 | Conserved hypothetical protein | 6.50e-49 | - | - | -0.878776 |
| PAAG_06730.2 | Conserved hypothetical protein | 2.37e-48 | - | - | -0.702441 |
| PAAG_05163.2 | Conserved hypothetical protein | 2.78e-48 | - | - | -0.831989 |
| PAAG_07217.2 | Conserved hypothetical protein | 3.71e-48 | - | - | -0.819074 |
| PAAG_05096.2 | Conserved hypothetical protein | 5.32e-47 | - | - | -0.880305 |
| PAAG_08187.2 | Conserved hypothetical protein | 1.34e-46 | - | - | -0.586953 |
| PAAG_07376.2 | Conserved hypothetical protein | 2.36e-46 | - | - | -1.2744 |
| PAAG_08808.2 | Conserved hypothetical protein | 3.94e-46 | - | - | -0.643347 |
| PAAG_00189.2 | Conserved hypothetical protein | 1.37e-45 | - | - | -0.79361 |
| [PAAG_11990.2](http://www.broadinstitute.org/annotation/genome/paracoccidioides_brasiliensis/FeatureRedirect.html?sp=S7000010310430228) | Conserved hypothetical protein | 2.62e-45 | - | - | -1.01766 |
| PAAG_01530.2 | Conserved hypothetical protein | 2.80e-45 | - | - | -1.18144 |
| PAAG_07808.2 | Conserved hypothetical protein | 3.33e-45 | - | - | -0.619073 |
| PAAG_00013.2 | Conserved hypothetical protein | 6.83e-45 | - | - | -0.613162 |
| PAAG_01862.2 | Conserved hypothetical protein | 1.61e-44 | - | - | -0.755485 |
| PAAG_05467.2 | Conserved hypothetical protein | 3.82e-44 | - | - | -0.58506 |
| PAAG_12642.2 | Conserved hypothetical protein | 2.63e-43 | - | - | -0.612875 |
| PAAG_01364.2 | Conserved hypothetical protein | 2.56e-41 | - | - | -1.77442 |
| PAAG_08312.2 | Conserved hypothetical protein | 8.05e-41 | - | - | -0.915566 |
| PAAG_03833.2 | Conserved hypothetical protein | 5.69e-40 | - | - | -0.944325 |
| PAAG_04300.2 | Conserved hypothetical protein | 6.53e-38 | - | - | -1.04554 |
| PAAG_08111.2 | Conserved hypothetical protein | 2.38e-37 | - | - | -0.660834 |
| PAAG_00854.2 | Conserved hypothetical protein | 1.58e-35 | - | - | -0.77435 |
| PAAG_07443.2 | Conserved hypothetical protein | 6.59e-35 | - | - | -0.696136 |
| PAAG_01211.2 | Conserved hypothetical protein | 1.25e-34 | - | - | -1.25703 |
| PAAG_07479.2 | Conserved hypothetical protein | 6.76e-34 | - | - | -0.728688 |
| PAAG_04114.2 | Conserved hypothetical protein | 3.57e-33 | - | - | -0.815562 |
| PAAG_01996.2 | Conserved hypothetical protein | 6.08e-33 | - | - | -0.817931 |
| PAAG_04093.2 | Conserved hypothetical protein | 1.32e-32 | - | - | -1.67167 |
| PAAG_03195.2 | Conserved hypothetical protein | 2.08e-32 | - | - | -1.31513 |
| PAAG_01190.2 | Conserved hypothetical protein | 2.15e-32 | - | - | -0.737747 |
| PAAG_08428.2 | Conserved hypothetical protein | 2.30e-31 | - | - | -0.645027 |
| PAAG_04968.2 | Conserved hypothetical protein | 4.04e-30 | - | - | -1.15057 |
| PAAG_05494.2 | Conserved hypothetical protein | 1.34e-27 | - | - | -0.797766 |
| PAAG_01170.2 | Conserved hypothetical protein | 5.58e-27 | - | - | -0.620897 |
| PAAG_03050.2 | Conserved hypothetical protein | 2.35e-26 | - | - | -0.94568 |
| PAAG_08844.2 | Conserved hypothetical protein | 6.23e-26 | - | - | -0.883307 |
| PAAG_02579.2 | Conserved hypothetical protein | 3.85e-25 | - | - | -0.64085 |
| PAAG_04732.2 | Conserved hypothetical protein | 1.76e-24 | - | - | -1.1311 |
| PAAG_03798.2 | Conserved hypothetical protein | 3.12e-23 | - | - | -0.818173 |
| PAAG_08094.2 | Conserved hypothetical protein | 6.33e-23 | - | - | -0.783247 |
| PAAG_07915.2 | Conserved hypothetical protein | 2.65e-65 | - | - | -1.08959 |
| PAAG_02807.2 | Conserved hypothetical protein | 1.11e-21 | - | - | -0.944083 |
| PAAG_00361.2 | Conserved hypothetical protein | 1.53e-21 | - | - | -0.81399 |
| PAAG_06414.2 | Conserved hypothetical protein | 1.73e-21 | - | - | -0.750576 |
| PAAG_07033.2 | Conserved hypothetical protein | 1.11e-20 | - | - | -0.80129 |
| PAAG_08073.2 | Conserved hypothetical protein | 1.30e-20 | - | - | -1.15125 |
| PAAG_03715.2 | Conserved hypothetical protein | 3.06e-20 | - | - | -0.744451 |
| PAAG_07052.2 | Conserved hypothetical protein | 1.31e-19 | - | - | -1.02234 |
| PAAG_02256.2 | Conserved hypothetical protein | 1.42e-19 | - | - | -0.802808 |
| PAAG_04593.2 | Conserved hypothetical protein | 2.27e-18 | - | - | -0.659646 |
| PAAG_07827.2 | Conserved hypothetical protein | 4.32e-18 | - | - | -0.915488 |
| PAAG_05343.2 | Conserved hypothetical protein | 7.40e-18 | - | - | -0.620309 |
| PAAG_01083.2 | Conserved hypothetical protein | 9.51e-18 | - | - | -0.662308 |
| PAAG_08330.2 | Conserved hypothetical protein | 2.08e-17 | - | - | -0.619321 |
| PAAG_08101.2 | Conserved hypothetical protein | 1.15e-16 | - | - | -0.634414 |
| PAAG_08777.2 | Conserved hypothetical protein | 1.56e-16 | - | - | -1.01854 |
| PAAG_01059 Não tem mais | Conserved hypothetical protein | 1.67e-16 | - | - | -0.775861 |
| PAAG_07219.2 | Conserved hypothetical protein | 1.84e-16 | - | - | -0.766929 |
| PAAG_04948.2 | Conserved hypothetical protein | 3.77e-16 | - | - | -0.684544 |
| PAAG_04911.2 | Conserved hypothetical protein | 6.18e-16 | - | - | -0.684523 |
| PAAG_00943.2 | Conserved hypothetical protein | 8.81e-16 | - | - | -0.78182 |
| PAAG_05173.2 | Conserved hypothetical protein | 1.98e-15 | - | - | -0.855409 |
| PAAG_01737.2 | Conserved hypothetical protein | 2.47e-15 | - | - | -0.591247 |
| PAAG_02696.2 | Conserved hypothetical protein | 2.95e-15 | - | - | -0.595944 |
| PAAG_07911.2 | Conserved hypothetical protein | 5.67e-15 | - | - | -1.15381 |
| PAAG_05624.2 | Conserved hypothetical protein | 6.83e-15 | - | - | -0.761701 |
| PAAG_00750.2 | Conserved hypothetical protein | 2.73e-16 | - | - | -0.673645 |
| [PAAG_12004.2](http://www.broadinstitute.org/annotation/genome/paracoccidioides_brasiliensis/FeatureRedirect.html?sp=S7000010310350487) | Conserved hypothetical protein | 8.19e-15 | - | - | -1.0133 |
| PAAG_08139.2 | Conserved hypothetical protein | 8.20e-15 | - | - | -0.632367 |
| PAAG_00761.2 | Conserved hypothetical protein | 9.11e-15 | - | - | -0.68256 |
| PAAG_03552.2 | Conserved hypothetical protein | 9.50e-15 | - | - | -1.02003 |
| PAAG_06778.2 | Conserved hypothetical protein | 1.03e-14 | - | - | -0.96543 |
| PAAG_05603.2 | Conserved hypothetical protein | 6.25e-14 | - | - | -0.653727 |
| PAAG_00391.2 | Conserved hypothetical protein | 6.38e-14 | - | - | -0.632484 |
| PAAG_07701.2 | Conserved hypothetical protein | 1.00e-13 | - | - | -0.853108 |
| PAAG_01775.2 | Conserved hypothetical protein | 1.23e-13 | - | - | -0.637144 |
| PAAG_02834.2 | Conserved hypothetical protein | 2.03e-13 | - | - | -0.658846 |
| PAAG_06942.2 | Conserved hypothetical protein | 1.47e-12 | - | - | -0.713109 |
| [PAAG_12107.2](http://www.broadinstitute.org/annotation/genome/paracoccidioides_brasiliensis/FeatureRedirect.html?sp=S7000010310407533) | Conserved hypothetical protein | 1.49e-12 | - | - | -0.741802 |
| PAAG_03430.2 | Conserved hypothetical protein | 1.36e-11 | - | - | -0.692072 |
| PAAG_00600.2 | Conserved hypothetical protein | 1.68e-11 | - | - | -0.731955 |
| PAAG_00585.2 | Conserved hypothetical protein | 3.35e-11 | - | - | -1.21806 |
| PAAG_00278.2 | Conserved hypothetical protein | 7.25e-11 | - | - | -0.621176 |
| PAAG_04635.2 | Conserved hypothetical protein | 7.44e-11 | - | - | -1.06318 |
| PAAG_05713.2 | Conserved hypothetical protein | 2.06e-10 | - | - | -0.594043 |
| PAAG_05408.2 | Conserved hypothetical protein | 2.96e-10 | - | - | -1.79085 |
| PAAG_03584.2 | Conserved hypothetical protein | 4.53e-10 | - | - | -0.786273 |
| PAAG_04736.2 | Conserved hypothetical protein | 4.77e-10 | - | - | -0.697017 |
| PAAG_08154.2 | Conserved hypothetical protein | 5.58e-10 | - | - | -0.68508 |
| PAAG_07692.2 | Conserved hypothetical protein | 1.53e-09 | - | - | -0.920278 |
| PAAG_03200.2 | Conserved hypothetical protein | 1.97e-09 | - | - | -0.982753 |
| PAAG_05567.2 | Conserved hypothetical protein | 2.43e-09 | - | - | -1.17124 |
| PAAG_00683.2 | Conserved hypothetical protein | 2.76e-09 | - | - | -1.45417 |
| PAAG_02356.2 | Conserved hypothetical protein | 3.89e-09 | - | - | -1.11161 |
| PAAG_05965.2 | Conserved hypothetical protein | 4.91e-09 | - | - | -0.594939 |
| PAAG_04643.2 | Conserved hypothetical protein | 5.18e-09 | - | - | -1.25216 |
| PAAG_02142.2 | Conserved hypothetical protein | 5.42e-09 | - | - | -0.754836 |
| PAAG_06420.2 | Conserved hypothetical protein | 7.85e-09 | - | - | -1.03596 |
| PAAG_03638.2 | Conserved hypothetical protein | 8.35e-09 | - | - | -0.939684 |
| PAAG_08339.2 | Conserved hypothetical protein | 1.80e-08 | - | - | -0.623917 |
| PAAG_06204.2 | Conserved hypothetical protein | 3.19e-08 | - | - | -1.01873 |
| PAAG_03492.2 | Conserved hypothetical protein | 5.56e-08 | - | - | -0.826778 |
| PAAG_00728.2 | Conserved hypothetical protein | 7.87e-08 | - | - | -0.705428 |
| PAAG_07402.2 | Conserved hypothetical protein | 1.07e-07 | - | - | -0.663503 |
| PAAG_05804.2 | Conserved hypothetical protein | 1.09e-07 | - | - | -0.621356 |
| PAAG_00571.2 | Conserved hypothetical protein | 1.14e-07 | - | - | -0.842914 |
| PAAG_01240.2 | Conserved hypothetical protein | 1.24e-08 | - | - | -0.715017 |
| PAAG_06433.2 | Conserved hypothetical protein | 3.14e-13 | - | - | -0.812585 |
| PAAG_05514.2 | Conserved hypothetical protein | 1.38e-07 | - | - | -0.615504 |
| PAAG_05473.2 | Conserved hypothetical protein | 1.39e-07 | - | - | -1.00974 |
| PAAG_08169.2 | Conserved hypothetical protein | 1.95e-07 | - | - | -0.651866 |
| PAAG_02322.2 | Conserved hypothetical protein | 1.95e-07 | - | - | -0.817603 |
| PAAG_11514.2 | Hypothetical protein Conserved hypothetical protein | 3.99e-07 | - | - | -0.602872 |
| PAAG_05923.2 | Conserved hypothetical protein | 4.89e-07 | - | - | -0.647496 |
| PAAG_01772.2 | Conserved hypothetical protein | 6.82e-07 | - | - | -0.941264 |
| PAAG_06849.2 | Conserved hypothetical protein | 8.74e-07 | - | - | -0.815096 |
| PAAG_06605.2 | Conserved hypothetical protein | 1.18e-06 | - | - | -0.781514 |
| PAAG_06366.2 | Conserved hypothetical protein | 1.41e-06 | - | - | -0.653862 |
| PAAG_06920.2 | Conserved hypothetical protein | 1.64e-06 | - | - | -0.590616 |
| PAAG_00446.2 | Conserved hypothetical protein | 1.72e-06 | - | - | -0.608645 |
| PAAG_08124.2 | Conserved hypothetical protein | 1.94e-06 | - | - | -0.881239 |
| PAAG_05122.2 | Conserved hypothetical protein | 4.03e-06 | - | - | -0.803023 |
| PAAG_06543.2 | Conserved hypothetical protein | 4.37e-06 | - | - | -1.19061 |
| PAAG_06928.2 | Conserved hypothetical protein | 4.63e-06 | - | - | -0.898221 |
| PAAG_07754.2 | Conserved hypothetical protein | 7.08e-06 | - | - | -1.01612 |
| PAAG_05854.2 | Conserved hypothetical protein | 8.31e-06 | - | - | -0.877832 |
| PAAG_02988.2 | Conserved hypothetical protein | 8.42e-06 | - | - | -0.964059 |
| PAAG_00208.2 | Conserved hypothetical protein | 1.08e-05 | - | - | -0.669198 |
| PAAG_05307.2 | Conserved hypothetical protein | 1.09e-05 | - | - | -0.606296 |
| PAAG_01560.2 | Conserved hypothetical protein | 1.35e-05 | - | - | -0.684352 |
| PAAG_03371.2 | Conserved hypothetical protein | 2.00e-05 | - | - | -1.26184 |
| PAAG_09026.2 | Conserved hypothetical protein | 2.41e-05 | - | - | -0.596102 |
| PAAG_03818.2 | Conserved hypothetical protein | 2.70e-05 | - | - | -1.2571 |
| PAAG_01762.2 | Conserved hypothetical protein | 3.19e-05 | - | - | -0.687437 |
| PAAG_07939.2 | Conserved hypothetical protein | 3.43e-05 | - | - | -1.45253 |
| PAAG_01590.2 | Conserved hypothetical protein | 8.15e-05 | - | - | -0.619657 |
| PAAG_02146.2 | Conserved hypothetical protein | 8.92e-05 | - | - | -1.00413 |
| PAAG_08768.2 | Conserved hypothetical protein | 8.94e-05 | - | - | -0.938579 |
| PAAG_01504.2 | Conserved hypothetical protein | 9.11e-05 | - | - | -0.756202 |
| PAAG_02716.2 | Conserved hypothetical protein | 9.29e-05 | - | - | -1.05116 |
| PAAG_11055.2 | Conserved hypothetical protein | 0 | - | - | -0.709792 |
| PAAG_02247.2 | Conserved hypothetical protein | 0 | - | - | -0.957115 |
| PAAG_11336.2 | Conserved hypothetical protein | 0 | - | - | -0.724174 |
| PAAG_08752.2 | Conserved hypothetical protein | 0 | - | - | -0.84464 |
| PAAG_04072.2 | Conserved hypothetical protein | 0 | - | - | -0.590465 |
| PAAG_01912.2 | Conserved hypothetical protein | 0 | - | - | -0.841091 |
| PAAG_00963.2 | Conserved hypothetical protein | 0 | - | - | -0.663969 |
| PAAG_06553.2 | Conserved hypothetical protein | 0 | - | - | -1.02042 |
| PAAG_08822.2 | Conserved hypothetical protein | 0 | - | - | -1.55457 |
| PAAG_01107.2 | Conserved hypothetical protein | 2.90e-06 | - | - | -0.94568 |
| PAAG_00881.2 | Conserved hypothetical protein | 0 | - | - | -0.899793 |
| PAAG_06339.2 | Conserved hypothetical protein | 0 | - | - | -0.85405 |
| PAAG_09101.2 | Conserved hypothetical protein | 0 | - | - | -0.994362 |
| PAAG_08495.2 | Conserved hypothetical protein | 0 | - | - | -0.612583 |
| PAAG_05947.2 | Conserved hypothetical protein | 0 | - | - | -0.908206 |
| PAAG_08894.2 | Conserved hypothetical protein | 0 | - | - | -1.34116 |
| PAAG_04520.2 | Conserved hypothetical protein | 0 | - | - | -0.815096 |
| PAAG_01721.2 | Hypothetical protein | 5.86e-125 | - | - | -2.25678 |
| PAAG_04334.2 | Hypothetical protein | 7.69e-122 | - | - | -0.602766 |
| PAAG_01391 | Hypothetical protein | 1.29e-227 | - | - | -0.648267 |
| PAAG_04741.2 | Hypothetical protein | 1.52e-103 | - | - | -2.1229 |
| PAAG_02434.2 | Hypothetical protein | 0 | - | - | -1.05363 |
| PAAG_00687 | Hypothetical protein | 0 | - | - | -0.885432 |
| PAAG_01199.2 | Hypothetical protein | 0 | - | - | -0.816259 |
| PAAG_12435.2 | Hypothetical protein | 0 | - | - | -2.02846 |
| PAAG_12435.2 | Hypothetical protein | 0 | - | - | -1.94012 |
| PAAG_07685.2 | Hypothetical protein | 2.49e-248 | - | - | -1.94399 |
| PAAG_01391 | Hypothetical protein | 1.29e-227 | - | - | -0.648267 |
| PAAG_01814 | Hypothetical protein | 7.07e-167 | - | - | -1.23085 |
| PAAG_07684 | Hypothetical protein | 1.35e-83 | - | - | -1.7801 |
| PAAG_02586.2 | Hypothetical protein | 2.49e-81 | - | - | -0.984431 |
| PAAG_06528.2 | Hypothetical protein | 4.52e-70 | - | - | -1.97715 |
| PAAG_04299.2 | Hypothetical protein | 1.26e-54 | - | - | -0.985695 |
| PAAG_08461.2 | Hypothetical protein | 2.02e-52 | - | - | -1.17821 |
| PAAG_04798.2 | Hypothetical protein | 4.70e-49 | - | - | -1.69232 |
| PAAG_07976.2 | Hypothetical protein | 1.20e-46 | - | - | -1.16357 |
| PAAG_04582.2 | Hypothetical protein | 8.04e-44 | - | - | -0.864139 |
| PAAG_00213.2 | Hypothetical protein | 3.98e-43 | - | - | -1.08924 |
| PAAG_00306.2 | Hypothetical protein | 3.16e-42 | - | - | -0.714703 |
| PAAG_03227.2 | Hypothetical protein | 4.55e-38 | - | - | -0.744969 |
| PAAG_07576.2 | Hypothetical protein | 2.68e-34 | - | - | -1.34579 |
| PAAG_08559.2 | Hypothetical protein | 1.68e-33 | - | - | -0.823799 |
| PAAG_11479.2 | Hypothetical protein | 6.24e-33 | - | - | -0.85183 |
| PAAG_01198 | Hypothetical protein | 1.85e-32 | - | - | -0.888397 |
| PAAG_04625.2 | Predicted protein (Hypothetical protein | 4.38e-32 | - | - | -0.874411 |
| PAAG_08814.2 | Hypothetical protein | 1.23e-31 | - | - | -1.96077 |
| PAAG_04333.2 | Hypothetical protein | 1.63e-31 | - | - | -0.606237 |
| PAAG_03597 | Hypothetical protein | 2.48e-30 | - | - | -0.856375 |
| PAAG_02240.2 | Hypothetical protein | 3.59e-30 | - | - | -1.1541 |
| PAAG_01486 | Hypothetical protein | 1.33e-29 | - | - | -0.775542 |
| PAAG_04342.2 | Hypothetical protein | 1.13e-28 | - | - | -0.842139 |
| PAAG_12064.2 | Hypothetical protein | 1.68e-28 | - | - | -2.32967 |
| PAAG_07453.2 | Predicted proteinHypothetical protein | 5.16e-28 | - | - | -1.58315 |
| PAAG_11333.2 | Hypothetical protein | 8.08e-28 | - | - | -1.192 |
| PAAG_11333.2 | Hypothetical protein | 8.29e-28 | - | - | -1.34803 |
| [PAAG_12531.2](http://www.broadinstitute.org/annotation/genome/paracoccidioides_brasiliensis/FeatureRedirect.html?sp=S7000010310473279) | Hypothetical protein | 2.10e-26 | - | - | -1.12876 |
| PAAG_01212.2 | Hypothetical protein | 2.13e-26 | - | - | -1.04401 |
| PAAG_05974.2 | Hypothetical protein | 6.12e-26 | - | - | -0.926429 |
| PAAG_02707.2 | Hypothetical protein | 1.90e-23 | - | - | -0.824418 |
| PAAG_00078.2 | Hypothetical protein | 8.66e-23 | - | - | -0.877876 |
| PAAG_03832.2 | Hypothetical protein | 4.08e-22 | - | - | -0.845535 |
| PAAG_04090.2 | Hypothetical protein | 4.94e-22 | - | - | -0.883 |
| PAAG_06453.2 | Hypothetical protein | 2.14e-21 | - | - | -0.684881 |
| PAAG_01702.2 | Hypothetical protein | 1.50e-20 | - | - | -0.726145 |
| PAAG_06883.2 | Hypothetical protein | 2.55e-20 | - | - | -0.682225 |
| PAAG_01155.2 | Hypothetical protein | 6.35e-20 | - | - | -0.716412 |
| PAAG_05190.2 | Hypothetical protein | 1.15e-19 | - | - | -0.820872 |
| PAAG_04680.2 | Hypothetical protein | 3.10e-19 | - | - | -1.29504 |
| PAAG_02242.2 | Hypothetical protein | 6.02e-19 | - | - | -0.63624 |
| PAAG_03720.2 | Hypothetical protein | 1.88e-18 | - | - | -1.22087 |
| PAAG_00421.2 | Hypothetical protein | 6.37e-17 | - | - | -0.699619 |
| PAAG_03179.2 | Hypothetical protein | 9.11e-17 | - | - | -0.99227 |
| PAAG_04439.2 | Hypothetical protein | 1.88e-16 | - | - | -1.28051 |
| PAAG_01458.2 | Hypothetical protein | 2.00e-16 | - | - | -0.99034 |
| PAAG_07134.2 | Hypothetical protein | 2.99e-16 | - | - | -0.802962 |
| PAAG_05973.2 | Hypothetical protein | 6.37e-16 | - | - | -1.03374 |
| PAAG_04115.2 | Hypothetical protein | 6.96e-16 | - | - | -0.875888 |
| PAAG_05477.2 | Hypothetical protein | 2.89e-15 | - | - | -0.736952 |
| PAAG_02955.2 | Hypothetical protein | 1.24e-14 | - | - | -0.809558 |
| PAAG_05742.2 | Hypothetical protein | 2.79e-14 | - | - | -0.663898 |
| PAAG_05314.2 | Hypothetical protein | 4.07e-14 | - | - | -0.742716 |
| PAAG_04067.2 | Hypothetical protein | 4.99e-14 | - | - | -0.645171 |
| PAAG_08791.2 | Hypothetical protein | 5.99e-14 | - | - | -0.867958 |
| PAAG_07096.2 | Hypothetical protein | 7.30e-14 | - | - | -0.709018 |
| PAAG_07149.2 | Hypothetical protein | 7.87e-14 | - | - | -0.626919 |
| PAAG_05658.2 | Hypothetical protein | 1.82e-13 | - | - | -0.980648 |
| PAAG_00129.2 | Hypothetical protein | 1.99e-13 | - | - | -1.03717 |
| PAAG_01006.2 | Hypothetical protein | 3.10e-13 | - | - | -0.851483 |
| PAAG_09087.2 | Hypothetical protein | 7.22e-13 | - | - | -0.685813 |
| PAAG_04683.2 | Hypothetical protein | 9.53e-13 | - | - | -0.81379 |
| PAAG_00134.2 | Hypothetical protein | 1.36e-12 | - | - | -0.794292 |
| PAAG_03361.2 | Hypothetical protein | 1.37e-12 | - | - | -0.819154 |
| PAAG_00341.2 | Hypothetical protein | 1.51e-12 | - | - | -0.598428 |
| PAAG_02167.2 | Hypothetical protein | 1.75e-12 | - | - | -0.751526 |
| PAAG_00254.2 | Hypothetical protein | 2.51e-12 | - | - | -1.17329 |
| PAAG_01901.2 | Hypothetical protein | 3.24e-12 | - | - | -0.822836 |
| PAAG_02062.2 | Hypothetical protein | 3.27e-12 | - | - | -1.74843 |
| PAAG_05540.2 | Hypothetical protein | 4.06e-12 | - | - | -1.12737 |
| PAAG_08778.2 | Hypothetical protein | 4.51e-12 | - | - | -1.49317 |
| PAAG_03362.2 | Hypothetical protein | 4.73e-12 | - | - | -1.08981 |
| PAAG_07926.2 | Hypothetical protein | 5.11e-12 | - | - | -2.72763 |
| PAAG_07442.2 | Hypothetical protein | 5.79e-12 | - | - | -0.605733 |
| PAAG_08877.2 | Hypothetical protein | 7.33e-12 | - | - | -1.09234 |
| PAAG_00311.2 | Hypothetical protein | 1.09e-11 | - | - | -0.999474 |
| PAAG_05364.2 | Hypothetical protein | 1.12e-11 | - | - | -0.588436 |
| PAAG_02947.2 | Hypothetical protein | 3.30e-11 | - | - | -0.92085 |
| PAAG_00465.2 | Hypothetical protein | 4.53e-11 | - | - | -1.21594 |
| PAAG_07097.2 | Hypothetical protein | 9.30e-11 | - | - | -0.778754 |
| PAAG_07898.2 | Hypothetical protein | 1.27e-10 | - | - | -1.1641 |
| PAAG_07454.2 | Hypothetical protein | 1.78e-10 | - | - | -1.00639 |
| PAAG_05177.2 | Hypothetical protein | 1.91e-10 | - | - | -0.900343 |
| PAAG_03360.2 | Hypothetical protein | 2.77e-10 | - | - | -0.866457 |
| PAAG_01180.2 | Hypothetical protein | 2.94e-10 | - | - | -0.943395 |
| PAAG_01636.2 | Hypothetical protein | 3.16e-10 | - | - | -0.894982 |
| PAAG_04797.2 | Hypothetical protein | 4.47e-10 | - | - | -1.33023 |
| PAAG_01392.2 | Hypothetical protein | 5.23e-10 | - | - | -1.31629 |
| PAAG_07095.2 | Hypothetical protein | 5.34e-10 | - | - | -0.845153 |
| PAAG_08235.2 | Hypothetical protein | 6.28e-10 | - | - | -0.699619 |
| PAAG_02849.2 | Hypothetical protein | 8.95e-10 | - | - | -1.40675 |
| PAAG_04768.2 | Hypothetical protein | 1.27e-09 | - | - | -0.676173 |
| PAAG_07758.2 | Hypothetical protein | 1.94e-09 | - | - | -1.11367 |
| PAAG_04694.2 | Hypothetical protein | 1.95e-09 | - | - | -0.640874 |
| PAAG_08544.2 | Hypothetical protein | 3.30e-09 | - | - | -0.847031 |
| PAAG_05223.2 | Hypothetical protein | 4.88e-09 | - | - | -0.790465 |
| PAAG_08654.2 | Hypothetical protein | 8.31e-09 | - | - | -2.17906 |
| PAAG_03653.2 | Hypothetical protein | 8.40e-09 | - | - | -1.26122 |
| PAAG_05104.2 | Hypothetical protein | 9.81e-09 | - | - | -0.592704 |
| PAAG_01049.2 | Hypothetical protein | 1.06e-08 | - | - | -0.807059 |
| PAAG_08290.2 | Hypothetical protein | 1.24e-08 | - | - | -0.766651 |
| PAAG_04924.2 | Hypothetical protein | 2.23e-08 | - | - | -0.918775 |
| PAAG_06025.2 | Hypothetical protein | 2.47e-08 | - | - | -1.1113 |
| PAAG_06514.2 | Hypothetical protein | 2.51e-08 | - | - | -0.635953 |
| PAAG_04900.2 | Hypothetical protein | 2.88e-08 | - | - | -0.849689 |
| PAAG_01839.2 | Hypothetical protein | 3.80e-08 | - | - | -0.897713 |
| PAAG_00818.2 | Hypothetical protein | 4.23e-08 | - | - | -0.612882 |
| PAAG_06648.2 | Hypothetical protein | 4.24e-08 | - | - | -0.857131 |
| PAAG_05263.2 | Hypothetical protein | 4.52e-08 | - | - | -0.754635 |
| PAAG_07914.2 | Hypothetical protein | 1.05e-07 | - | - | -0.686809 |
| PAAG_06711.2 | Hypothetical protein | 1.18e-07 | - | - | -1.20537 |
| PAAG_08779.2 | Hypothetical protein | 1.38e-07 | - | - | -1.2537 |
| PAAG_04234.2 | Hypothetical protein | 1.57e-07 | - | - | -1.39853 |
| PAAG_05512.2 | Hypothetical protein | 2.03e-07 | - | - | -1.22356 |
| PAAG_04405.2 | Hypothetical protein | 2.05e-07 | - | - | -0.625441 |
| PAAG_00222.2 | Hypothetical protein | 2.69e-07 | - | - | -0.839164 |
| PAAG_05298.2 | Hypothetical protein | 3.33e-07 | - | - | -1.46569 |
| PAAG_06729.2 | Hypothetical protein | 4.18e-07 | - | - | -0.72265 |
| PAAG_01582.2 | Hypothetical protein | 4.57e-07 | - | - | -0.899938 |
| PAAG_05525.2 | Hypothetical protein | 4.67e-07 | - | - | -1.92201 |
| PAAG_08443.2 | Hypothetical protein | 5.81e-07 | - | - | -0.758102 |
| PAAG_03218.2 | Hypothetical protein | 5.84e-07 | - | - | -1.10085 |
| PAAG_02956.2 | Hypothetical protein | 5.87e-07 | - | - | -0.829035 |
| PAAG_03035.2 | Hypothetical protein | 7.58e-07 | - | - | -0.743146 |
| PAAG_06106.2 | Hypothetical protein | 9.55e-07 | - | - | -1.29153 |
| PAAG_04322.2 | Hypothetical protein | 1.05e-06 | - | - | -0.817895 |
| PAAG_01404.2 | Hypothetical protein | 1.13e-06 | - | - | -0.607261 |
| PAAG_03327.2 | Hypothetical protein | 1.58e-06 | - | - | -0.705144 |
| PAAG_08390.2 | Hypothetical protein | 1.63e-06 | - | - | -1.23408 |
| PAAG_03679.2 | Hypothetical protein | 1.71e-06 | - | - | -1.36072 |
| PAAG_06481.2 | Hypothetical protein | 1.87e-06 | - | - | -0.750076 |
| PAAG_05600.2 | Hypothetical protein | 2.11e-06 | - | - | -1.46146 |
| PAAG_01386.2 | Hypothetical protein | 2.30e-06 | - | - | -1.13769 |
| PAAG_05755.2 | Hypothetical protein | 2.33e-06 | - | - | -1.11466 |
| PAAG_01079.2 | Hypothetical protein | 2.50e-06 | - | - | -0.711637 |
| PAAG_01562.2 | Hypothetical protein | 2.53e-06 | - | - | -0.727056 |
| PAAG_01820.2 | Hypothetical protein | 2.78e-06 | - | - | -0.714764 |
| PAAG_00853.2 | Hypothetical protein | 2.86e-06 | - | - | -1.46075 |
| PAAG_05728.2 | Hypothetical protein | 2.90e-06 | - | - | -0.893992 |
| PAAG_08514.2 | Hypothetical protein | 3.13e-06 | - | - | -1.10984 |
| PAAG_02032.2 | Hypothetical protein | 3.40e-06 | - | - | -1.56717 |
| PAAG_06308.2 | Hypothetical protein | 3.63e-06 | - | - | -1.25216 |
| PAAG_06787.2 | Hypothetical protein | 3.75e-06 | - | - | -0.651672 |
| PAAG_06267.2 | Hypothetical protein | 3.98e-06 | - | - | -0.735592 |
| PAAG_00784.2 | Hypothetical protein | 4.13e-06 | - | - | -1.04441 |
| PAAG_05035.2 | Hypothetical protein | 4.55e-06 | - | - | -1.00253 |
| PAAG_08195.2 | Hypothetical protein | 5.28e-06 | - | - | -0.749508 |
| PAAG_01742.2 | Hypothetical protein | 6.17e-06 | - | - | -0.586277 |
| PAAG_01619.2 | Hypothetical protein | 6.56e-06 | - | - | -1.30052 |
| PAAG_04122.2 | Hypothetical protein | 7.06e-06 | - | - | -0.763257 |
| PAAG_07596.2 | Hypothetical protein | 7.29e-06 | - | - | -1.67958 |
| PAAG_00737.2 | Hypothetical protein | 8.26e-06 | - | - | -0.629414 |
| PAAG_03071.2 | Hypothetical protein | 8.67e-06 | - | - | -1.02478 |
| PAAG_01313.2 | Hypothetical protein | 8.70e-06 | - | - | -1.82759 |
| PAAG_03862.2 | Hypothetical protein | 8.71e-06 | - | - | -0.80837 |
| PAAG_06190.2 | Hypothetical protein | 9.36e-06 | - | - | -1.01057 |
| PAAG_00055.2 | Hypothetical protein | 9.42e-06 | - | - | -1.42446 |
| PAAG_06992.2 | Hypothetical protein | 1.04e-05 | - | - | -0.996492 |
| PAAG_07862.2 | Hypothetical protein | 1.11e-05 | - | - | -0.925693 |
| PAAG_06534.2 | Hypothetical protein | 1.19e-05 | - | - | -0.753759 |
| PAAG_07166.2 | Hypothetical protein | 1.28e-05 | - | - | -1.42278 |
| PAAG_00701.2 | Hypothetical protein | 1.28e-05 | - | - | -1.02254 |
| PAAG_04066.2 | Hypothetical protein | 1.33e-05 | - | - | -0.796844 |
| PAAG_08373.2 | Hypothetical protein | 1.57e-05 | - | - | -0.595048 |
| PAAG_04992.2 | Hypothetical protein | 1.67e-05 | - | - | -0.953802 |
| PAAG_00499.2 | Hypothetical protein | 1.67e-05 | - | - | -0.80129 |
| PAAG_02863.2 | Hypothetical protein | 1.77e-05 | - | - | -1.42102 |
| PAAG_08609.2 | Hypothetical protein | 1.78e-05 | - | - | -0.589523 |
| PAAG_07790.2 | Hypothetical protein | 1.90e-05 | - | - | -0.591232 |
| PAAG_00554.2 | Hypothetical protein | 1.93e-05 | - | - | -0.729774 |
| PAAG_04267.2 | Hypothetical protein | 2.19e-05 | - | - | -0.993094 |
| PAAG_03487.2 | Hypothetical protein | 2.36e-05 | - | - | -0.769877 |
| PAAG_03424.2 | Hypothetical protein | 2.56e-05 | - | - | -0.588516 |
| PAAG_08496.2 | Hypothetical protein | 2.83e-05 | - | - | -0.721792 |
| PAAG_07627.2 | Hypothetical protein | 2.88e-05 | - | - | -0.663093 |
| PAAG_08470.2 | Hypothetical protein | 2.98e-05 | - | - | -1.03797 |
| PAAG_00541.2 | Hypothetical protein | 3.23e-05 | - | - | -1.72449 |
| PAAG_05111.2 | Hypothetical protein | 3.44e-05 | - | - | -1.06575 |
| PAAG_02165.2 | Hypothetical protein | 3.56e-05 | - | - | -0.690768 |
| PAAG_02741.2 | Hypothetical protein | 3.69e-05 | - | - | -1.53897 |
| PAAG_00677.2 | Hypothetical protein | 4.13e-05 | - | - | -0.764714 |
| PAAG_04316.2 | Hypothetical protein | 4.67e-05 | - | - | -1.11256 |
| PAAG_03797.2 | Hypothetical protein | 4.92e-05 | - | - | -0.623229 |
| PAAG_05801.2 | Hypothetical protein | 5.63e-05 | - | - | -0.75915 |
| PAAG_05821.2 | Hypothetical protein | 6.39e-05 | - | - | -1.67005 |
| PAAG_06799.2 | Hypothetical protein | 6.59e-05 | - | - | -1.49317 |
| PAAG_02519.2 | Hypothetical protein | 7.36e-05 | - | - | -0.768403 |
| PAAG_08474.2 | Hypothetical protein | 8.15e-05 | - | - | -0.785349 |
| PAAG_02450.2 | Hypothetical protein | 8.74e-05 | - | - | -1.7562 |
| PAAG_03580.2 | Hypothetical protein | 9.11e-05 | - | - | -0.756202 |
| PAAG_07029.2 | Hypothetical protein | 9.80e-05 | - | - | -0.691497 |
| PAAG_07585.2 | Hypothetical protein | 9.81e-05 | - | - | -2.07813 |
| PAAG_02535.2 | Hypothetical protein | 9.85e-05 | - | - | -2.40006 |
| PAAG_01815.2 | Hypothetical protein | 0 | - | - | -0.689565 |
| PAAG_00922.2 | Hypothetical protein | 0 | - | - | -1.20686 |
| PAAG_03800.2 | Hypothetical protein | 0 | - | - | -1.16455 |
| PAAG_07287.2 | Hypothetical protein | 0 | - | - | -0.874731 |
| PAAG_06480.2 | Hypothetical protein | 0 | - | - | -0.699619 |
| PAAG_06302.2 | Hypothetical protein | 0 | - | - | -0.746925 |
| PAAG_06901.2 | Hypothetical protein | 0 | - | - | -0.721645 |
| PAAG_03270.2 | Hypothetical protein | 0 | - | - | -2.60865 |
| PAAG_05409.2 | Hypothetical protein | 0 | - | - | -2.60865 |
| PAAG_00075.2 | Hypothetical protein | 0 | - | - | -0.634783 |
| PAAG_02957.2 | Hypothetical protein | 0 | - | - | -0.949546 |
| PAAG_08213.2 | Hypothetical protein | 0 | - | - | -0.842914 |
| PAAG_04713.2 | Hypothetical protein | 0 | - | - | -2.7562 |
| PAAG_09120.2 | Hypothetical protein | 0 | - | - | -1.93374 |
| PAAG_08240.2 | Hypothetical protein | 0 | - | - | -1.22398 |
| PAAG_07877.2 | Hypothetical protein | 0 | - | - | -0.932052 |
| PAAG_05565.2 | Hypothetical protein | 0 | - | - | -3.23013 |
| PAAG_02587.2 | Hypothetical protein | 0 | - | - | -0.860225 |
| PAAG_05544.2 | Hypothetical protein | 0 | - | - | -1.19361 |
| PAAG_06905.2 | Hypothetical protein | 0 | - | - | -0.893255 |
| PAAG_01166.2 | Hypothetical protein | 0 | - | - | -0.865137 |
| PAAG_06457.2 | Hypothetical protein | 0 | - | - | -1.15028 |
| PAAG_08554.2 | Hypothetical protein | 0 | - | - | -0.749508 |
| PAAG_01537.2 | Hypothetical protein | 0 | - | - | -1.44254 |
| PAAG_06232.2 | Hypothetical protein | 0 | - | - | -0.643728 |
| PAAG_03248.2 | Hypothetical protein | 0 | - | - | -1.28963 |
| PAAG_03293.2 | Hypothetical protein | 0 | - | - | -0.660519 |
| PAAG_09084.2 | Hypothetical protein | 0 | - | - | -0.679581 |
| PAAG_07630.2 | Hypothetical protein | 0 | - | - | -0.857324 |
| PAAG_04579.2 | Hypothetical protein | 0 | - | - | -0.667564 |
| PAAG_00757.2 | Hypothetical protein | 0 | - | - | -2.38625 |
| PAAG_05217.2 | Hypothetical protein | 0 | - | - | -1.63067 |
| PAAG_05276.2 | Hypothetical protein | 0 | - | - | -1.49317 |
| PAAG_07747.2 | Hypothetical protein | 0 | - | - | -0.633346 |
| PAAG_02832.2 | Hypothetical protein | 0 | - | - | -1.13471 |
| PAAG_07783.2 | Hypothetical protein | 0 | - | - | -0.712734 |
| PAAG_02104.2 | Hypothetical protein | 0 | - | - | -1.14803 |
| PAAG_07179.2 | Hypothetical protein | 0 | - | - | -0.784934 |
| PAAG_03699.2 | Hypothetical protein | 0 | - | - | -0.586277 |
| PAAG_07403.2 | Hypothetical protein | 0 | - | - | -1.5573 |
| PAAG_02321.2 | Hypothetical protein | 0 | - | - | -1.3126 |
| PAAG_07439.2 | Hypothetical protein | 0 | - | - | -4.30052 |
| PAAG_08660.2 | Hypothetical protein | 0 | - | - | -1.7562 |
| PAAG_08143.2 | Hypothetical protein | 0 | - | - | -0.627818 |
| PAAG_05456.2 | Hypothetical protein | 0 | - | - | -0.832655 |
| PAAG_02514.2 | Hypothetical protein | 0 | - | - | -0.676475 |
| PAAG_00862.2 | Hypothetical protein | 0 | - | - | -0.729868 |
| PAAG_05430.2 | Hypothetical protein | 0 | - | - | -0.988695 |
| PAAG_07797.2 | Hypothetical protein | 0 | - | - | -0.792728 |
| PAAG_01635.2 | Hypothetical protein | 0 | - | - | -1.56717 |
| PAAG_04247.2 | Hypothetical protein | 0 | - | - | -0.821222 |
| PAAG_07137.2 | Hypothetical protein | 0 | - | - | -0.889827 |
| PAAG_03677.2 | Hypothetical protein | 0 | - | - | -0.939232 |
| PAAG_09093.2 | Hypothetical protein | 6.66e-06 | - | - | -0.612263 |
| PAAG_05470.2 | Hypothetical protein | 9.41e-07 | - | - | -0.668002 |
| PAAG_02827.2 | Hypothetical protein | 5.71e-35 | - | - | -0.932944 |
| PAAG_03518.2 | Hypothetical protein | 9.77e-24 | - | - | -0.844445 |
| PAAG_08307.2 | Hypothetical protein | 1.44e-12 | - | - | -0.841608 |
| PAAG_01225.2 | Hypothetical protein | 3.22e-09 | - | - | -1.80804 |
| PAAG_01742.2 | Hypothetical protein | 6.17e-06 | - | - | 0.586277 |
| PAAG_01522.2 | Hypothetical protein | 1.44e-07 | - | - | -1.12502 |
| PAAG_01507.2 | Hypothetical protein | 1.35e-11 | - | - | -0.957913 |
| PAAG_08515.2 | Hypothetical protein | 0 | - | - | -3.46023 |
| PAAG_06305.2 | Hypothetical protein | 2.87e-233 | - | - | -1.62733 |
| PAAG_04708.2 | Hypothetical protein | 3.01e-171 | - | - | -1.4875 |
| PAAG_08440.2 | Hypothetical protein | 2.29e-08 | - | - | -0.792728 |
